# Distinct hormonal and morphological control of dormancy and germination in *Chenopodium album* dimorphic seeds

**DOI:** 10.3389/fpls.2023.1156794

**Published:** 2023-03-30

**Authors:** Eddison Loades, Marta Pérez, Veronika Turečková, Danuše Tarkowská, Miroslav Strnad, Anne Seville, Kazumi Nakabayashi, Gerhard Leubner-Metzger

**Affiliations:** ^1^ Department of Biological Sciences, Royal Holloway University of London, Egham, United Kingdom; ^2^ Laboratory of Growth Regulators, Faculty of Science, Palacký University and Institute of Experimental Botany, Czech Academy of Sciences, Olomouc, Czechia; ^3^ Crop Protection Research, Syngenta, Jealott’s Hill International Research Centre, Bracknell, United Kingdom

**Keywords:** coat-imposed dormancy, gibberellins, hormone metabolism, seed coat properties, seed heteromorphism, thermal time modelling, abscisic acid, weed seed bank

## Abstract

Dormancy and heteromorphism are innate seed properties that control germination timing through adaptation to the prevailing environment. The degree of variation in dormancy depth within a seed population differs considerably depending on the genotype and maternal environment. Dormancy is therefore a key trait of annual weeds to time seedling emergence across seasons. Seed heteromorphism, the production of distinct seed morphs (in color, mass or other morphological characteristics) on the same individual plant, is considered to be a bet-hedging strategy in unpredictable environments. Heteromorphic species evolved independently in several plant families and the distinct seed morphs provide an additional degree of variation. Here we conducted a comparative morphological and molecular analysis of the dimorphic seeds (black and brown) of the Amaranthaceae weed *Chenopodium album*. Freshly harvested black and brown seeds differed in their dormancy and germination responses to ambient temperature. The black seed morph of seedlot #1 was dormant and 2/3^rd^ of the seed population had non-deep physiological dormancy which was released by after-ripening (AR) or gibberellin (GA) treatment. The deeper dormancy of the remaining 1/3^rd^ non-germinating seeds required in addition ethylene and nitrate for its release. The black seeds of seedlot #2 and the brown seed morphs of both seedlots were non-dormant with 2/3^rd^ of the seeds germinating in the fresh mature state. The dimorphic seeds and seedlots differed in testa (outer seed coat) thickness in that thick testas of black seeds of seedlot #1 conferred coat-imposed dormancy. The dimorphic seeds and seedlots differed in their abscisic acid (ABA) and GA contents in the dry state and during imbibition in that GA biosynthesis was highest in brown seeds and ABA degradation was faster in seedlot #2. *Chenopodium* genes for GA and ABA metabolism were identified and their distinct transcript expression patterns were quantified in dry and imbibed *C. album* seeds. Phylogenetic analyses of the Amaranthaceae sequences revealed a high proportion of expanded gene families within the *Chenopodium* genus. The identified hormonal, molecular and morphological mechanisms and dormancy variation of the dimorphic seeds of *C. album* and other Amaranthaceae are compared and discussed as adaptations to variable and stressful environments.

## 1 Introduction

Predicting weed emergence in crop production systems is a global challenge that requires understanding mechanisms of weed ecology and trait evolution in response to climate change and altered agricultural practices (Neve et al., 2009; Westwood et al., 2018; Nakabayashi and Leubner-Metzger, 2021). The life-history strategy of weeds seems to be a blend of phenotypic plasticity and local adaptation for which within-species and within-seedlot variation in seed dormancy depth, as well as variation in dormancy release requirements are key to weed emergence timing (Baskin and Baskin, 2006; Walck et al., 2011; Batlla et al., 2020). The primary dormancy of freshly harvested mature seeds is established during seed maturation and its depth depends on the environmental conditions during the mother plant’s reproductive growth (Karssen, 1970; Penfield and MacGregor, 2017; Fernández Farnocchia et al., 2021). The control of germination by dormancy can be considered as block(s) to the completion of germination of an intact viable seed under otherwise favorable conditions, namely when the seed becomes non-dormant (Finch-Savage and Leubner-Metzger, 2006). The regulation of dormancy and germination in response to ambient environmental factors such as temperature is achieved, at least in part, by the balance and sensitivity to abscisic acid (ABA) and gibberellins (GA). ABA biosynthesis and signaling dominate the dormant state, whereas GA biosynthesis and signaling dominate the transition to germination. Morphological aspects of seed dormancy and germination include embryo shape and size, and the biophysical properties of the seed coat, endosperm and other tissues which may confer coat dormancy (Chahtane et al., 2017; Steinbrecher and Leubner-Metzger, 2017; Baskin and Baskin, 2019; Holloway et al., 2021; Walker et al., 2021).

Most plants produce one kind of seeds and fruits (diaspore monomorphism) which may exhibit variation in dormancy depth within the seedlot. Interestingly, some species evolved a heteromorphism strategy in which an individual plant produces two (dimorphism) or more (heteromorphism) kinds of diaspores (Imbert, 2002; Matilla et al., 2005; Baskin et al., 2014; Liu et al., 2018; Gianella et al., 2021). The heteromorphic seeds may differ in characteristics such as shape, color, size or mass, and this is often accompanied by different dispersal mechanisms, germination characteristics and dormancy levels. Plants with seed heteromorphism are commonly annuals of disturbed sites and stressful environments. Diaspore heteromorphism is considered a bet-hedging strategy in adaptation to unpredictable environments. Examples for dimorphic annuals include the Brassicaceae *Aethionema arabicum* and *Diptychocarpus strictus* (Baskin et al., 2014; Lenser et al., 2016; Arshad et al., 2021) and the Amaranthaceae *Suaeda salsa* (Wang et al., 2015; Li et al., 2016; Xu et al., 2017), other *Suaeda* and *Atriplex* species (Baskin et al., 2014; Liu et al., 2018), and *Chenopodium album* (Yao et al., 2010). The seed morphs of these Amaranthaceae species differ in size and seed coat color (black versus brown) and their distinct adaptive roles has been investigated in saline habitats (Yao et al., 2010; Baskin et al., 2014; Liu et al., 2018). In general, the brown seeds were found to be non-dormant and more salt-tolerant compared to the dormant black seeds which form a persistent soil seed bank. Amaranthaceae seed internal structure is characterized by a peripheral embryo and by variation of seed coat thickness which determines the coat-imposed physiological dormancy (Karssen, 1970; Baskin and Baskin, 2019; Fernández Farnocchia et al., 2021; Nakabayashi and Leubner-Metzger, 2021).

*Chenopodium album* L. (common lambsquarter, fat-hen) is a cosmopolitan, annual weed species of notable economic importance (Bajwa et al., 2019; Krak et al., 2019). It is among the most competitive and difficult-to-control weeds in several cropping systems and has developed resistance to multiple herbicide mode of actions. Using the *C. album* aggregate as a model, Krak et al. (2019) revealed the importance of Asia as one of the main centers of diversity for this weedy species. This work also showed that human-mediated dispersal of ruderal and weed plant species accompanied the westward spread of agriculture in the Neolithic period. The successful competitiveness of *C. album* as a weed derived predominantly from the ability to persist in the soil bank in the seed stage through complex regulation of physiological seed dormancy (Bouwmeester and Karssen, 1989; Bouwmeester and Karssen, 1993; Yao et al., 2010). Yao et al. (2010) comparatively investigated the seed morphology, dormancy and germination physiology of black and brown seeds of two *C. album* populations from semi-arid and light-saline environments in China which produced a similar proportion of the two seed morphs. In contrast to this, *C. album* weed populations from arable land in Europe produce only a small proportion (approx. 3%) of brown seeds. Most previous research on *C. album* was therefore focussed on the germination strategy of the black seeds to a better control of this harmful weed (Karssen, 1968; Karssen, 1976a; Karssen, 1976b; Saini et al., 1985; Bouwmeester and Karssen, 1993; Murdoch and Roberts, 1997; Bajwa et al., 2019). The underpinning molecular mechanisms of the *C. album* dimorphic seed’s dormancy and germination are largely unknown.

In the present study, we comparatively investigate the brown and black seed morphs of two *C. album* populations with different origin in Great Britain, seedlot #1 from an arable field followed by glasshouse propagation, and seedlot #2 from direct collection in an urban environment. The hormonal, molecular and morphological mechanisms of the seed morphs and seedlots differed considerably. This provided insight into the complex regulation of their dormancies and its variation in depth between and within seed morphs and seedlots. These findings are compared with results from other Amaranthaceae species and discussed in the wider context of seed dimorphism and dormancy mechanisms in weed seed biology.

## 2 Results

### 2.1 Dimorphic seeds of *C. album* differ in dormancy, seed coat morphology and germination responses to ambient temperatures

**Figure 1:**
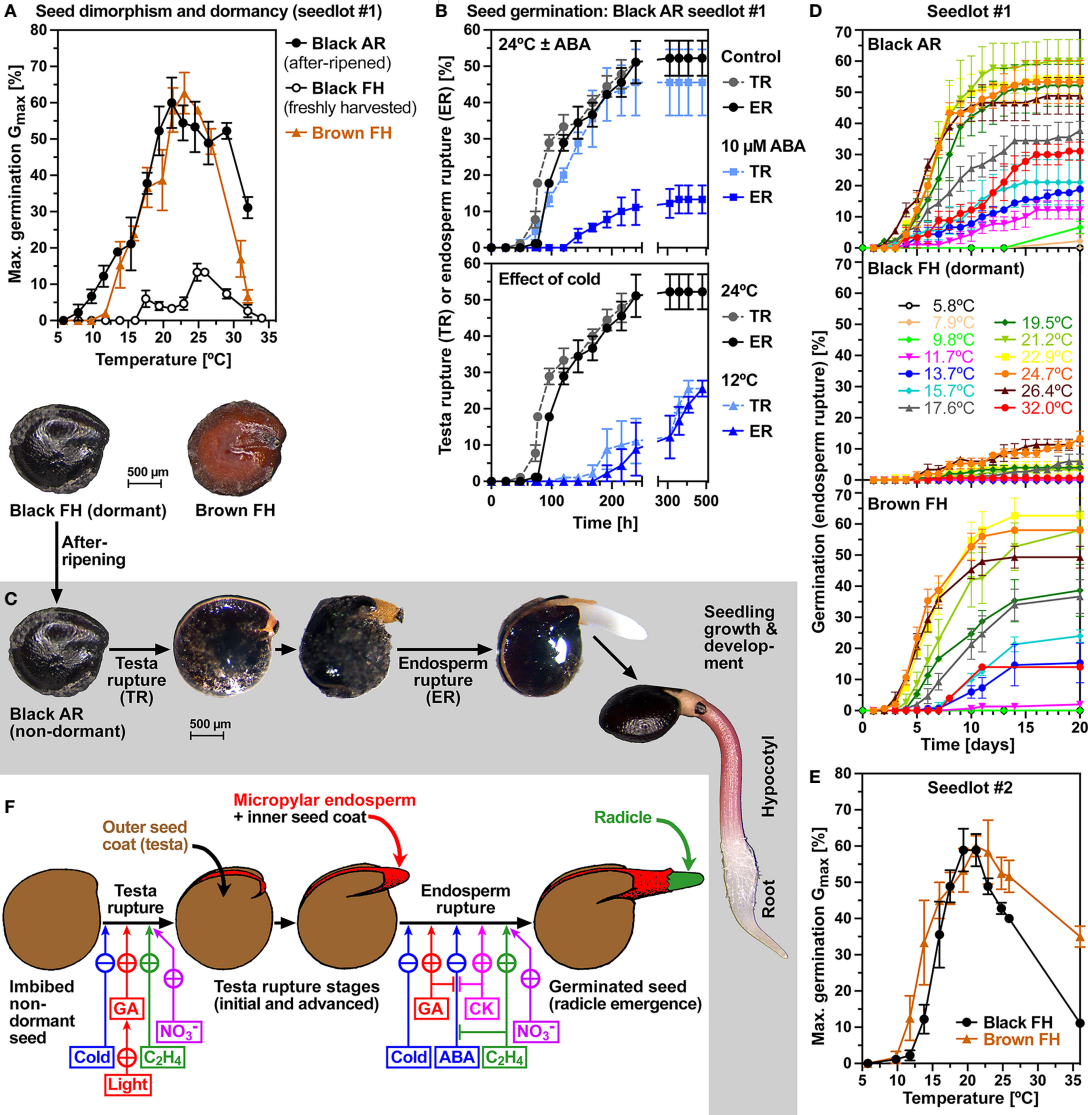
Germination physiology and temperature responses of *Chenopodium album* dimorphic seeds. **(A)** Seed dimorphism and temperature profile of *C. album* seedlot #1 maximum germination percentages (G_max_). The G_max_ comparison at the temperatures indicated of mature freshly harvested (FH) black and brown seeds and after-ripened (AR) seeds imbibed at 24°C. **(B)** The effect of *cis-S*(+)-abscisic acid (ABA) at 24°C and of cold temperature (12°C) on the time courses of testa rupture (TR) and endosperm rupture (ER). **(C)** Physiological stages and visible events during the germination and subsequent seedling growth of *C. album* dimorphic seeds. **(D)** The kinetics of germination (scored as ER over time) of seedlot #1 seed morphs at different temperatures. **(E)** Seed dimorphism and temperature profile of *C. album* seedlot #2 G_max_. **(F)** Control of visible events (TR, ER) during *C. album* germination and the effects of light, cold, nitrate and hormonal (ABA; GA, gibberellins; C_2_H_4_, ethylene; CK, cytokinins) treatments (this work and Karssen, 1976a; Karssen, 1976b); drawing of seed stages from (Karssen, 1976b) reproduced with permission. **(A-F)** Mean ± SEM values are presented of at least 3 petri dishes each with 30 seeds imbibed in continuous white light.

**Figure 1** shows that the dimorphic seeds of *C. album* seedlots #1 and #2 differ in color (seed morphs: black and brown), dormancy and germination responses to ambient temperatures. Freshly harvested (FH) mature seeds of seedlot #1 (obtained by propagating accession #1 in the glasshouse) contained ca. 95% black seeds which were dormant and did not germinate over a wide range of temperatures (**Figures 1A, D**). The maximum germination percentage (G_max_) of these dormant populations of black seeds was ca. 10% at 25°C. Three months of seed after-ripening (AR, i.e. dry storage at room temperature) caused dormancy release resulting in ca. 60% G_max_ and in widening of the permissive temperature window for germination of the black AR seeds (**Figures 1A, D**). In contrast to black seeds of seedlot #1 which were dormant in the FH state, black seeds of seedlot #2 (obtained by collecting seeds of accession #2 in the wild) were non-dormant upon harvest and germinated readily with ca. 60% G_max_ (**Figure 1E**). In contrast to black seeds, brown seeds of accessions #1 and #2 were non-dormant in the FH state and germinated readily with ca. 60% G_max_ without an AR storage requirement (**Figures 1A, D, E**). The distinct dimorphic seeds (black and brown) of these two distinct accessions (#1 and #2) were therefore highly suited to conduct a comparative analysis of the underpinning mechanisms.

Population-based thermal-time threshold modeling revealed that the non-dormant states of the black and brown seed morphs of seedlot #1 differed in their germination responses to ambient temperature (**Supplementary Figures 1A, B**). While the optimal (T_opt_) and base (T_base_) temperatures of black AR #1 seeds were 24°C and 8°C, respectively, the corresponding T_opt_ and T_base_ of brown seeds were ca. 2°C higher. In the sub-optimal (cold) temperature range the two morphs differed slightly in their thermal time constants (Θ_cold(50%)_) which exhibited broad distributions (large SD) around their 50% values (**Supplementary Figures 1A, B**). In the supra-optimal (warm) temperature range the ceiling temperature (T_c(50%)_) of the black seed population #1 was higher and more broadly distributed (35°C, large SD) compared to of the brown population (31°C, small SD) (**Supplementary Figures 1E, F**). In contrast to seedlot #1, the black and brown seed morphs of seedlot #2 were very similar in their T_opt_ (ca. 22°C) and T_base_ (ca. 10.5°C) temperatures, and their Θ_cold(50%)_ exhibited sharper distributions (small SDs) (**Supplementary Figures 1C, D**). The obtained T_opt_ (22-24°C) were very similar to published values (22-25°C), while the obtained T_base_ (8-10.5°C) temperatures were higher compared to the published values (3-6°C) for black *C. album* seeds (Karssen, 1976b; Murdoch et al., 1989; Roman et al., 1999; Guillemin et al., 2013). In these publications G_max_ values of ca. 90% were observed, but others obtained much lower G_max_ values of 50-60% for black *C. album* seeds even after 6 months of AR storage (Karssen, 1976a; Yao et al., 2010). The observed G_max_ of ca. 60% in our *C. album* seedlots #1 and #2 (**Figure 1**) was associated, even after 3 months of AR storage, with considerable fraction populations of non-germinating seeds (**Supplementary Figure 1**).

**Figure 2:**
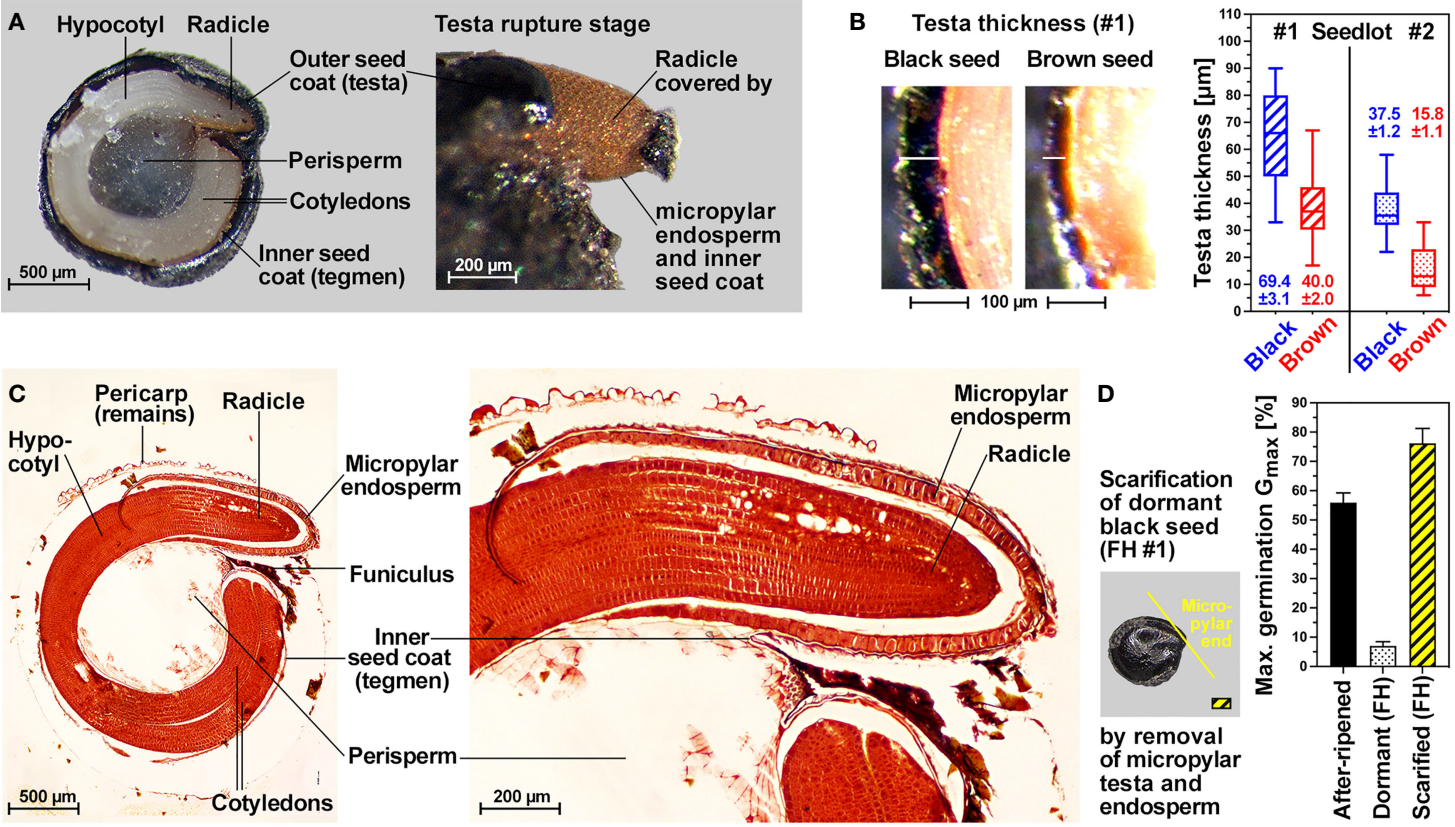
Internal morphology, seed coat scarification and testa thickness of *C. album* dimorphic seeds. **(A)** Cross-section of dry black seed and visible endosperm protrusion after TR. **(B)** Images and box-and-whisker plot (min-max) of testa thickness of black and brown seed. Mean ± SEM values are presented of at least 50 seeds of each seed type. **(C)** Microscopic cross-section highlighting tissue features of *C. album* seeds after having undergone methyl methylacrylate Technovit fixation embedding protocol stained in safranin and counter stained in methylene blue. **(D)** Maximum germination (mean ± SEM values) of seedlot #1 dormant black, after-ripened black and mechanically scarified dormant black seeds.

The internal morphology of *C. album* seeds is characterized by a peripheral embryo which is curled around the perisperm (**Figure 2**). The radicle and lower hypocotyl are covered with a living endosperm layer which is confined to the embryo’s radicle end and does not fully surround the entire embryo (**Figure 2C**). The embryo is however fully surrounded by a very thin inner seed coat (tegmen) and thicker hard outer seed coat (testa). Testa rupture (TR) and subsequent endosperm rupture (ER) are two visible events during the germination of black and brown *C. album* seeds (**Figure 1C**). At the TR stage with ruptured testa the expanding radicle remains covered by exposed micropylar endosperm and inner seed coat (**Figure 2A**). Subsequent ER rupture precedes the completion of germination by radicle emergence (**Figure 1C**). Light is known to be required for inducing TR (Karssen, 1976a) and abscisic acid (ABA) known to inhibit ER (Karssen, 1968) of black *C. album* seeds (**Figure 1F**). We demonstrate here that light is also required for the germination of brown *C. album* seeds (**Supplementary Figure 2A**), and that ABA delays germination by inhibiting ER without affecting the timing of TR of black (**Figure 1B**) and brown (**Supplementary Figure 2B**) seeds. Kinetin was shown to promote ER of black *C. album* seeds (Karssen, 1976a), but did not affect the seed germination of seedlots #1 and #2 (**Supplementary Figure 2C**). In contrast to light and ABA targeting either TR or ER, we found that cold temperature targets both processes and delayed *C. album* TR and ER (**Figure 1B**). The working model presented in **Figure 1F** summarizes how hormonal and environmental cues target *C. album* TR and ER.

Consistent with a role of testa thickness in *C. album* dormancy, black seeds of seedlot #1 (dormant) have a ca. 2-fold thicker testa compared to non-dormant brown seeds of seedlot #1 (**Figure 2B**). The testa thickness of black seeds of seedlot #2 (non-dormant) and brown seeds of seedlot #1 are roughly equal, and the testa of brown seeds of seedlot #2 is ca. 2-fold thinner (**Figure 2B**). It therefore seems that, at least for these two seed batches, that the dimorphic seeds differ in that the testas of brown seeds are thinner, and that the observed dormancy of black #1 seeds is associated with the thickest testa. Consistent with a role of the thick testa and the presence of coat dormancy in FH black #1 seeds, scarification of the dormant seed by seed coat removal at the micropylar end caused germination (**Figure 2D**). A scarification-induced G_max_ of ca. 75% was observed for black #1 seeds which exceeds the dormancy-releasing effect of after-ripening (G_max_ ca. 60% in AR black seeds, **Figure 1**). Together with the distinct responses to temperature and ABA (**Figure 1B**), the observed release of the coat-imposed dormancy suggests that it is controlled by the seed’s hormonal network.

### 2.2 Distinct hormonal regulation of dormancy and germination in dimorphic *C. album* seeds

**Figure 3:**
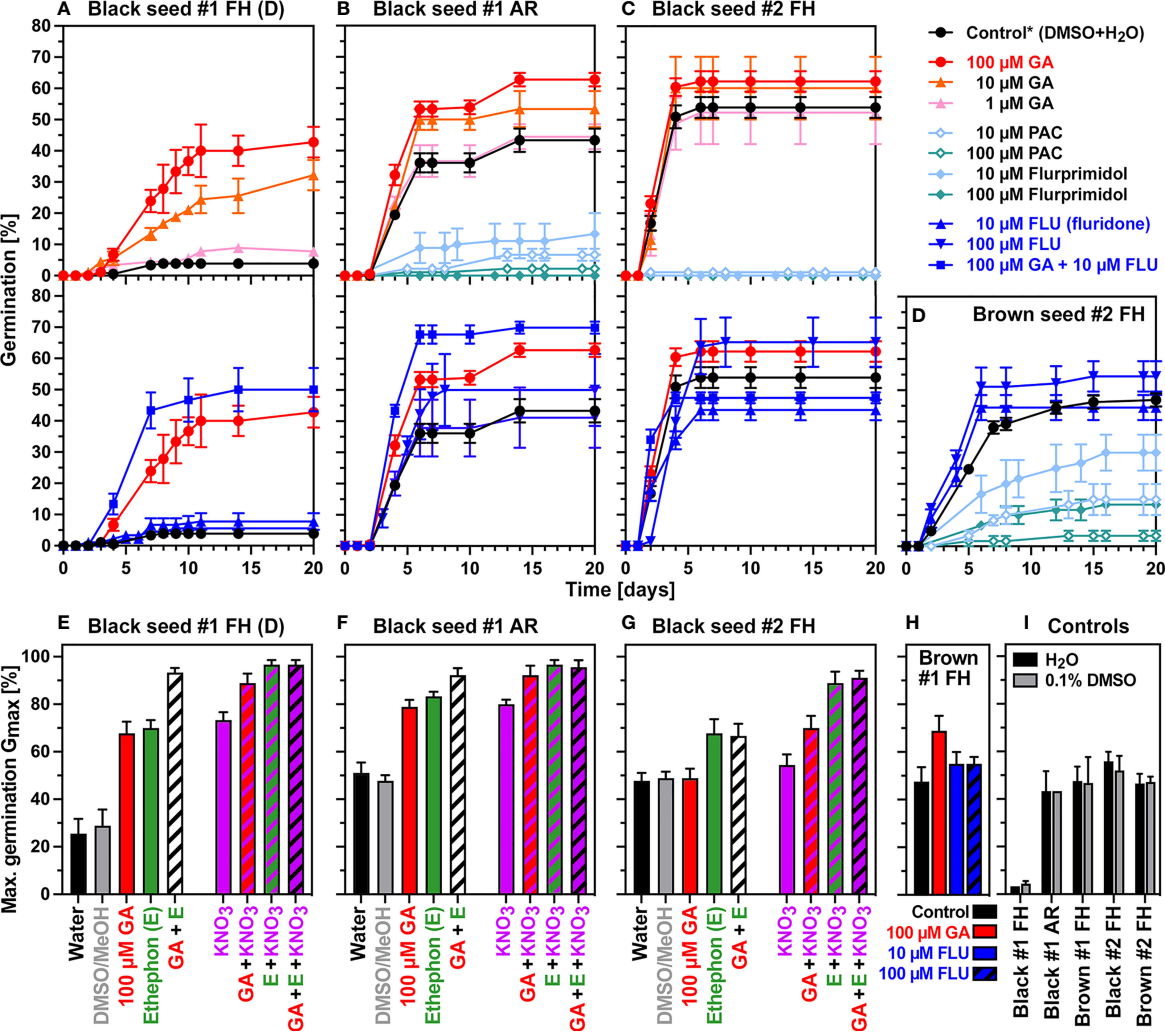
Germination responses of *C. album* dimorphic seeds to treatment with phytohormones and biosynthesis inhibitors. **(A-D)** Effects of gibberellins (GA_4+7_), flurprimidol, PAC, and fluridone (FLU), on the germination time courses of dormant black #1 **(A)**, AR black #1 **(B)**, black #2 **(C)**, and brown #2 **(D)** seeds. **(E-G)** Effects of 100 µM GA_4+7_ (GA), 3.5 mM ethephon (E, ethylene release compound), and 10 mM KNO_3_ on the maximal germination percentages of dormant black #1 **(E)**, AR black #1 **(F)**, and black #2 **(G)** seed populations. **(H)** Effects of GA and FLU on brown #1 seeds. **(I)** Maximal germination percentages of water (H_2_O) and 0.1% (v/v) dimethyl sulfoxide (DMSO) controls. Mean ± SEM values are presented. AR, after-ripened; D, dormant; FLU, fluridone; MeOH, methanol; PAC, paclobutrazol; * indicates mean value between DMSO and H2O controls.

**Figure 3** shows that gibberellins (GA) and ABA are key regulators of *C. album* seed dormancy and germination. Treatment experiments of FH black seeds of seedlot #1 with GA_4+7_ released their dormancy in a concentration-dependent manner with the 100 µM GA_4+7_ concentration exhibiting the highest dormancy-breaking activity (**Figure 3A**). While the carotenoid biosynthesis inhibitor fluridone (FLU, inhibits ABA biosynthesis) alone had no dormancy-releasing effect, the combined treatment of 100 µM GA_4+7_ plus FLU released the dormancy of FH black #1 seeds to the ca. 50-70% G_max_ values observed for AR black #1 seeds (**Figures 3A, B**). Treatment combinations of GA, ethephon (E, an ethylene-releasing compound), and KNO_3_ resulted in G_max_ values of >90% (**Figure 3E**). These results showed that GA is the predominant factor controlling dormancy release for the majority of dormant seeds, and that another fraction population of deeper dormant seeds require a more complex hormonal regulation which in addition to GA and ABA involves ethylene and nitrate signaling for the full dormancy release (**Figures 3E-G**). The black FH black seeds of seedlot #1 therefore exhibits two layers of physiological dormancy (PD): 2/3^rd^ of the population has nondeep PD (nPD) which can be released by GA and AR, and 1/3^rd^ of the population has a deeper PD (dPD) which cannot be released by GA and AR alone. This finding sheds new light on seed hetermorphism as a bet-hedging strategy to establish persistent seeds banks for this species.

Pharmacological experiments with AR black seeds of seedlot #1 (**Figure 3B**) and FH black seeds of seedlot #2 (**Figure 3C**) demonstrated that GA_4+7_ promoted the germination of non-dormant seeds. The use of the GA biosynthesis inhibitors paclobutrazol (PAC) and flurprimidol further demonstrated that GA biosynthesis is essentially required for the germination of black seeds. Treatment with the carotenoid/ABA biosynthesis inhibitor fluridone (FLU) alone did not appreciably affect the germination of black seeds, but when combined with 100 µM GA_4+7_ it enhanced the GA effect (**Figures 3B, C**). As for the dormancy release, treatment combinations of GA, E and KNO_3_ caused maximum promotion and resulted in G_max_ values of >90% (**Figures 3F, G**). The germination of FH brown seeds of seedlot #1 (**Figure 3H**) and seedlot #2 (**Supplementary Figure 2D**) was also promoted by GA_4+7_ treatment. Results with the GA biosynthesis inhibitors PAC and flurprimidol demonstrated that GA biosynthesis is also required for the germination of brown seeds (**Figure 3D**). From the different dose responses for PAC and flurprimidol we conclude that the germination of brown seeds may differ in their GA requirement or contents compared to black seeds (**Figure 3**). These findings highlight the importance of GA biosynthesis and GA-ABA interactions for *C. album* seed germination and suggests that the two accessions (#1 and #2) and the seed morphs (black and brown) differ in their hormone metabolism and sensitivities.

### 2.3 Complex GA and ABA metabolism during dimorphic seed dormancy and germination

**Figure 4:**
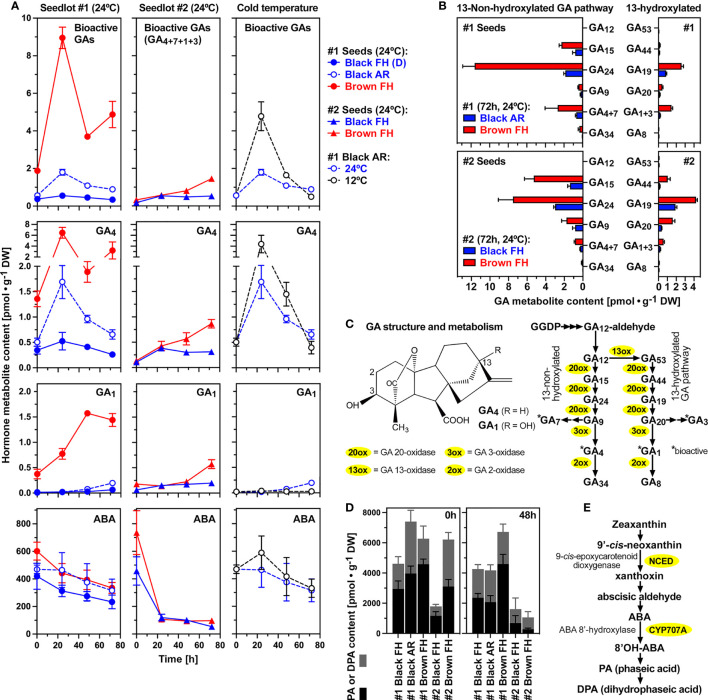
Gibberellin (GA) and abscisic acid (ABA) metabolite profiling during early imbibition of (*C*) *album* dimorphic seeds. **(A)** Temporal hormone metabolite content comparison of bioactive GAs and ABA in seedlots #1 and #2 at 24°C, and AR #1 black seeds at 12°C; Mean ± SEM values are presented. **(B)** GA biosynthesis pathway metabolite contents after 72h imbibition of dimorphic seeds from seedlots #1 and #2. **(C)** Molecular structure of bioactive C_19_-gibberellins GA_4_ and GA_1_ (*left*), GA metabolism and enzymes of the 13-non-hydroxylated and the 13-hydroxylated pathways (*right*). **(D)** Phaseic acid (PA) and dihydrophaseic acid (DPA) contents in dimorphic seeds of seedlots #1 and #2 in dry (*left*) and 48h imbibed (*right*) seeds. **(E)** ABA biosynthesis and catabolic pathway with key enzymes indicated.

**Figure 4** shows that the seed morphs (black and brown) and their dormancy state, as well as the accessions (#1 and #2) differ considerably in their GA and ABA metabolism. Bioactive GA (GA_4_, GA_7_, GA_1_, GA_3_) accumulated ca. 5-fold during the early imbibition of FH brown #1 seeds at 24°C (**Figure 4A**). A ca. 5-fold accumulation during the early imbibition was also observed in AR #1 black seeds, but the overall contents of bioactive GA accumulation in brown seeds were ca. 5-fold higher compared to AR black seeds. In contrast to brown and AR black seeds, no appreciable accumulation of bioactive GAs was observed in FH (D) black #1 seeds (**Figure 4A**). The contents of bioactive GAs in dry seeds of seedlot #1 were higher compared to dry seeds of seedlot #2. Starting from very low contents of bioactive GAs, accumulation during imbibition was also observed in brown #2 seeds, but the overall contents of bioactive GA accumulation remained much lower compared to brown #1 seeds (**Figure 4A**). Bioactive GA accumulation also occurred during the early imbibition of black #2 seeds, and remained lower compared to brown #2 seeds as it was the case for the #1 seed morphs (**Figure 4A**). These results highlight the importance of bioactive GA production in non-dormant seeds which is consistent with the results obtained with the GA biosynthesis inhibitors demonstrating that *de novo* GA biosynthesis was required for the completion of seed germination (**Figure 3**). Brown seeds of seedlot #2 were more tolerant to GA biosynthesis inhibitors compared to black seeds (**Figure 3D**), which could be due to the observed increased *de novo* GA biosynthesis and bioactive GA accumulation in brown seeds compared to black seeds (**Figure 4**).

The primary bioactive C_19_-GA accumulating during *C. album* seed germination was GA_4_ (**Figure 4A**), a product of the 13-non-hydroxylation GA biosynthesis pathway (**Figure 4C**). Accumulation of GA_1_, a C_19_-GA product of the 13-hydroxylation GA biosynthesis pathway, occurred to a lower extend, suggesting that the 13-non-hydroxylation GA biosynthesis pathway is dominant in *C. album* seeds (**Figure 4**). **Figure 4B** demonstrates that this is indeed the case in both seed morphs (black and brown) and both accessions (#1 and #2). Especially in brown seeds much higher contents of the 13-non-hydroxylated precursors GA_15_ and GA_24_ were observed as compared to the corresponding 13-hydroxylated GA_44_ and GA_19_ metabolites (**Figure 4B**). Detail time course analysis of all major GA metabolites demonstrates the dominance of the 13-non-hydroxylation pathway during imbibition (**Supplementary Figure 3**). In addition to GA_4_, also the 13-non-hydroxylated bioactive GA_7_ accumulated in seedlot #1, but not in seedlot #2. In contrast to this, the accumulation of 13-hydroxylated bioactive GA_1_ was not accompanied by appreciable GA_3_ accumulation (**Supplementary Figure 3**).

In contrast to the accumulation of bioactive GA, the ABA contents of seeds declined during imbibition (**Figure 4A**). This decline did not appreciably differ among seed states within each seedlot, but was more rapid in black and brown seeds of seedlot #2 compared to seedlot #1. This raised the question of whether biosynthesis or catabolism of ABA is regulated differently between the black and brown seed morphs or between dormant vs non-dormant seeds. ABA degradation by ABA 8’-hydroxylases (CYP707A) and the subsequent accumulation of phaseic acid (PA) and dihydrophaseic acid (DPA) metabolites (**Figure 4E**) was evident in dry and imbibed black and brown seeds of both accessions (**Figure 4D**). PA and DPA contents were significantly higher in the dry seeds of AR #1 black and #1 brown seeds compared to FH #1 black seeds (**Figure 4D**), and subsequently declined during imbibition consistent with ABA declining across these seed states (**Figure 4A**). Imbibition of AR black #1 seeds at cold temperature (12°C) enhanced the accumulation of bioactive GAs without appreciably affecting the decline in ABA levels (**Figure 4A**).

### 2.4 Identification and phylogeny of *Chenopodium* GA and ABA metabolism genes

Key enzymes in ABA and GA metabolism include 9-*cis*-epoxycarotenoid dioxygenases (NCED), ABA 8’-hydroxylases (CYP707A), and GA-oxidases for GA biosynthesis (GA20ox, GA3ox) and inactivation (GA2ox) (Nambara et al., 2010; Urbanova et al., 2011; Hedden, 2020). They are encoded by multigene families (Huang et al., 2015; Lange and Lange, 2020; Arshad et al., 2021; Li et al., 2021; Sabir et al., 2022) and for *Arabidopsis thaliana* the members expressed during seed dormancy and germination are well known (Ogawa et al., 2003; Kushiro et al., 2004; Lefebvre et al., 2006; Okamoto et al., 2006; Nambara et al., 2010). To identify *Chenopodium* sequences of GA and ABA metabolism genes expressed in seeds we mined the *Chenopodium quinoa* genome (Jarvis et al., 2017) and conducted BLAST analyses with the *A. thaliana* and *C. quinoa* sequences *via* TAIR and Phytozome (Goodstein et al., 2012) as presented in detail in **Supplementary Figure 4**. Using the obtained *C. quinoa* gene IDs we also identified differentially expressed genes (DEGs) during *C. quinoa* seed germination (**Supplementary Figure 5**) from published transcriptomes (Wu et al., 2020; Hao et al., 2022). The combined information was used for the successful PCR cloning of 14 C*. album* cDNA sequences for GA and ABA metabolism genes (**Supplementary Figure 6**).

**Figure 5:**
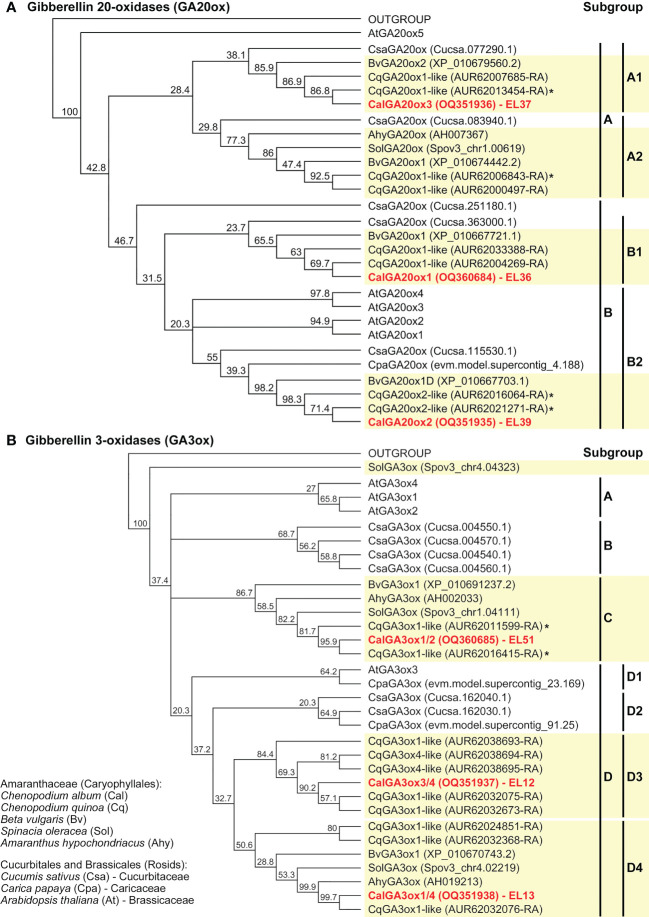
Phylogenetic tree of the predicted amino acid sequences of GA-oxidases. Known and putative amino acid GA20ox **(A)** and GA3ox **(B)** sequences of *Chenopodium quinoa*, *Chenopodium album* and other Amaranthaceae plus selected Brassicales and Cucurbitales species (as indicated) were aligned using ClustalW and Neighbor-Joining trees were built as described in methods. Note the expansion and diversification of the *Chenopodium* GA20ox and GA3ox gene subfamilies as contrasted to *Beta vulgaris, C. sativum* and *A. thaliana*. *Chenopodium quinoa* (**Supplementary Figure 4**) and *C. album* (**Supplementary Figure 6**) sequences (EL36, EL37, EL39, EL12, EL13, EL51) representing these subgroups were identified. An * indicates identified DEGs during *C. quinoa* seed germination (**Supplementary Figure 5**).

**Figure 6:**
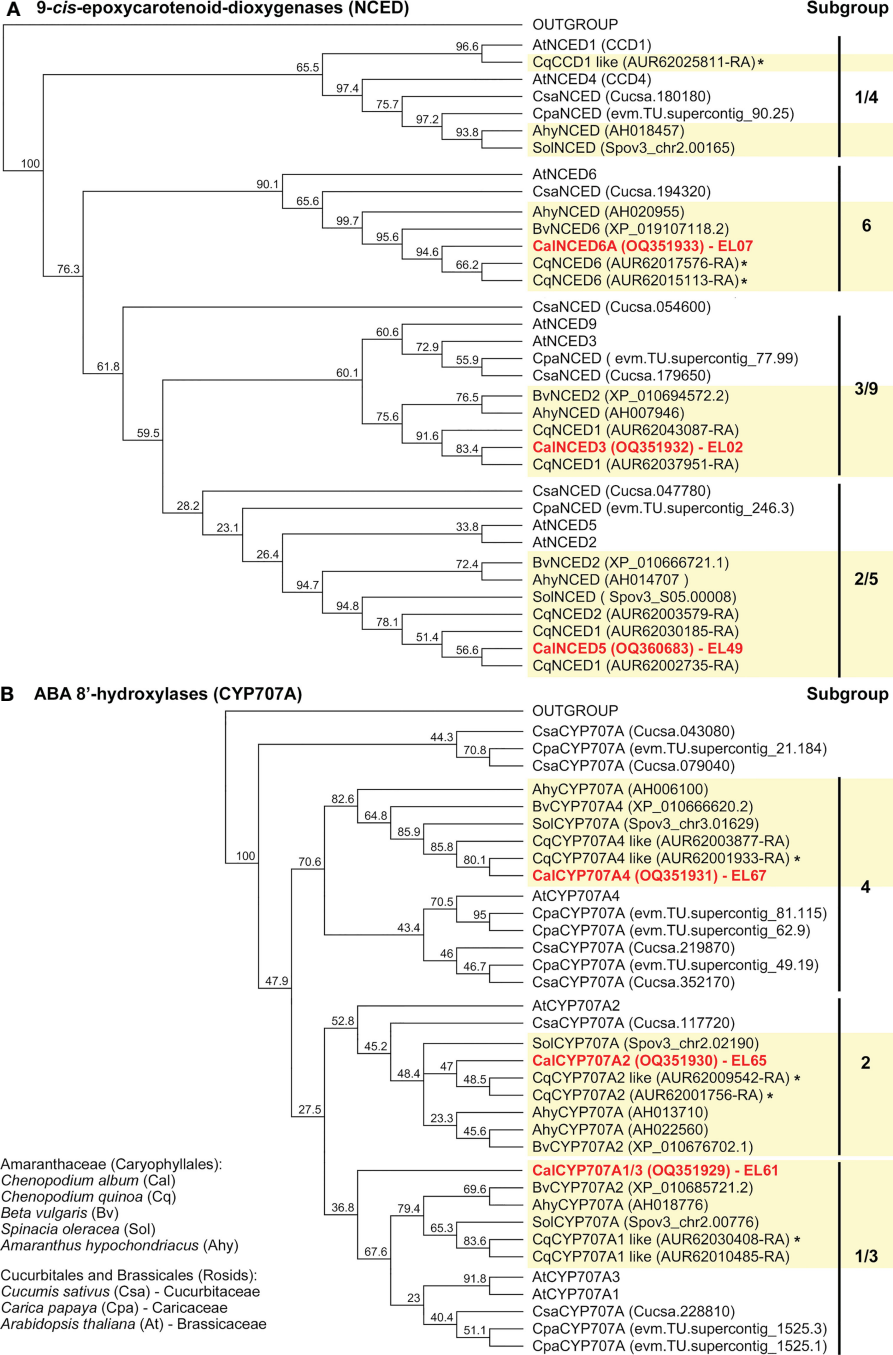
Phylogenetic tree of the predicted amino acid sequences of 9-*cis*-epoxycarotenoid dioxygenases (NCED) and ABA 8’-hydroxylases (CYP707A). Known and putative amino acid NCED **(A)** and CYP707A **(B)** sequences of *Chenopodium quinoa*, *Chenopodium album* and other Amaranthaceae plus selected Brassicales and Cucurbitales species (as indicated) were aligned using ClustalW and Neighbor-Joining trees were built as described in methods. *Chenopodium quinoa* (**Supplementary Figure 4**) and *C. album* (**Supplementary Figure 6**) sequences (EL02, EL07, EL49, EL61, EL65, EL67) representing the major subgroups were identified. An * indicates identified DEGs during *C.* *quinoa* seed germination (**Supplementary Figure 5**).

The phylogenetic trees for the GA (**Figure 5**) and ABA (**Figure 6** and **Supplementary Figure 7**) metabolism genes support the observed gene family expansion and diversification within the *Chenopodium* lineage of the Amaranthaceae (Ma et al., 2021). The identified *C. album* sequences cover different Amaranthaceae groups (**Figures 5**, **Figure 6**, and **Supplementary Figure 7**) and constitute putative orthologs to almost all the DEGs expressed in germinating *C. quinoa* seeds (**Supplementary Figure 5**). The economically important *C. quinoa* is an allotetraploid species with a sequenced genome (Jarvis et al., 2017). For the ABA metabolism genes (**Figure 6**) this expansion in *Chenopodium* was within each of the CYP707A1/3, CYP707A2, CYP707A4, NCED2/5, NCED3/9 and NCED6 subgroups known from *A. thaliana*. While each of these subgroups contained at least one *A. thaliana* type member, there was not in all subgroups contained a representative for *Beta vulgaris*, but each subgroup contained two to three *C. quinoa* representatives and *C. album* representative (**Figure 6**). For the GA oxidase genes, the BLAST analyses (**Supplementary Figure 4**) and phylogenetic trees suggest a more diversified gene expansion in the *Chenopodium* lineage. For the GA20ox (**Figure 5A**), GA3ox (**Figure 5B**) and GA2ox (**Supplementary Figure 7**) genes the Amaranthaceae formed subgroups which were in many cases distinct from the other species and often did not contain Arabidopsis genes. An example for this among the GA3ox genes are the D3 and D4 subgroups which contain only *Chenopodium* and Amaranthaceae sequences, respectively (**Figure 5B**). Corresponding Brassicales (*AtGA3ox3*, *Carica papaya*) and Cucurbitaceae (*Cucumis sativus*) sequences were in the D1 and D2 subgroups. Other *Arabidopsis* (*AtGA3ox1, AtGA3ox2, AtGA3ox4*), *Cucumis* and Amaranthaceae sequences cluster separately in the A, B and C subgroups. In contrast to GA20ox and GA3ox where an abundance of *Chenopodium* genes has evolved and forms multiple subgroups, for the GA2ox genes *A. thaliana* has a similar number and diversity (**Supplementary Figure 7**). The identified *C. album* GA2ox2 is a member of the C_19_-GA 2-oxidases which deactivate bioactive C_19_-GAs (Lange and Lange, 2020). The 14 identified *C. album* sequences (**Supplementary Figure 6**) cover almost each major *Chenopodium* subgroup of GAox20, GAox3, NCED and CYP707A (**Figures 5**, **Figure 6**) genes and were used to analyze their expression in imbibed seeds with RT-qPCR.

### 2.5 Transcript expression patterns of GA and ABA metabolism genes in *C. album* seeds

**Figure 7:**
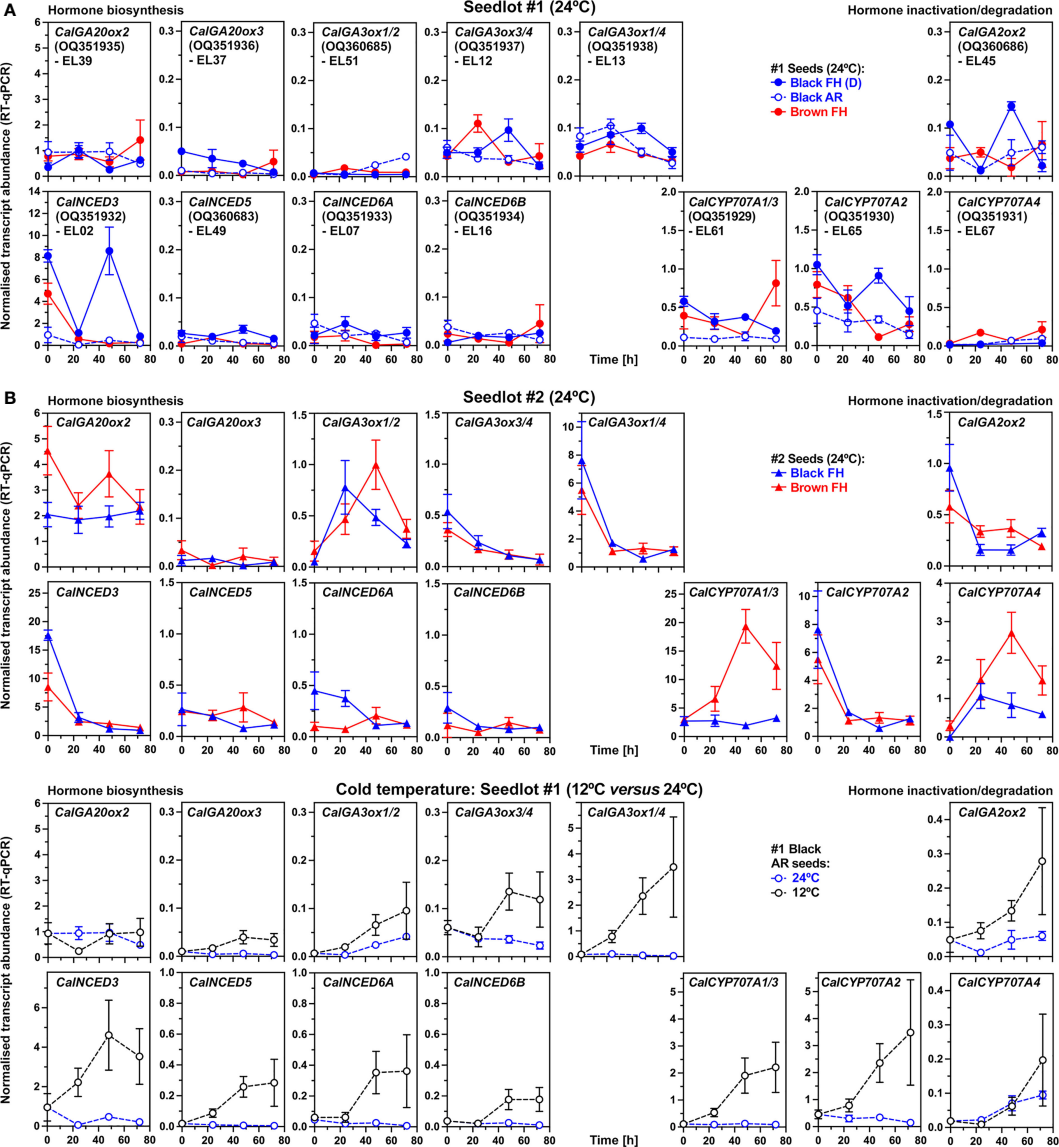
RT-qPCR analysis of transcript abundance patterns in *C. album* dimorphic seeds for key genes in gibberellin (GA) and abscisic acid (ABA) metabolism. **(A)** RT-qPCR analysis of seedlot #1 dimorphic seeds imbibed at 24°C. The normalized transcript abundances of *C. album* GA-oxidase (GA20ox, GA3ox, GA2ox), 9-*cis*-epoxycarotenoid dioxygenase (NCED) and ABA 8’-hydroxylase (CYP707A) genes. For details about identified *C. album* genes see main text and **Supplementary Figure 6**. **(B)** RT-qPCR analysis of seedlot #2 dimorphic seeds imbibed at 24°C. **(A)** RT-qPCR analysis of seedlot #1 AR black seeds imbibed at 12°C and 24°C. Mean ± SEM of at least 3 biological replicates.

Gibberellin 20-oxidases (GA20ox) catalyze the formation of the inactive C_19_-precursors GA_9_ and GA_20_ of bioactive GA_4_ and GA_1_, respectively (**Figure 4C**). Among the three identified *C. album* GA20-oxidases *CalGA20ox2* transcripts were most abundant in seeds, while *CalGA20ox3* was only weakly expressed (**Figure 7**). The *CalGA20ox2* transcript abundances in seeds of seedlot #2 were 2-4 fold higher compared to seedlot #1 at 24°C (**Figures 7A, B**), as were the GA_9_ and GA_20_ contents (**Figure 4B**). Gibberellin 3-oxidases (GA3ox) catalyze the 3-hydroxylation which converts the inactive direct precursors GA_9_ and GA_20_ into bioactive GA_4_ and GA_1_, respectively (**Figure 4C**). *CalGA3ox1/2* transcript abundances in seeds of seedlot #2 were higher compared to seedlot #1 at 24°C (**Figures 7A, B**). In seedlot #2 they also increased over time in parallel with bioactive GA accumulation, suggesting that the required GA3ox enzyme activity for the stronger accumulation of bioactive GA in seedlot #1 did not depend on additional transcript accumulation (**Figure 4A**). Transcript abundances of *CalGA2ox2*, encoding GA2ox which catalyzes GA inactivation by 2-hydroxylation (**Figure 4C**), were low and decreasing at 24°C except for dormant black #1 seeds (**Figure 7A**). Finally, the enhanced production of bioactive GA in cold-imbibed AR black #1 seeds (**Figure 4A**), was associated with the major accumulation of *CalGA3ox1/4*, and weak accumulation of *CalGA3ox1/2, CalGA3ox3/4* and *CalGA2ox2* transcripts (**Figure 7C**). Transcripts of their GA3ox and GA2ox orthologs accumulated during *C. quinoa* seed germination, while transcript abundances of GA20ox orthologs declined (**Supplementary Figure 5**).

**Figure 8:**
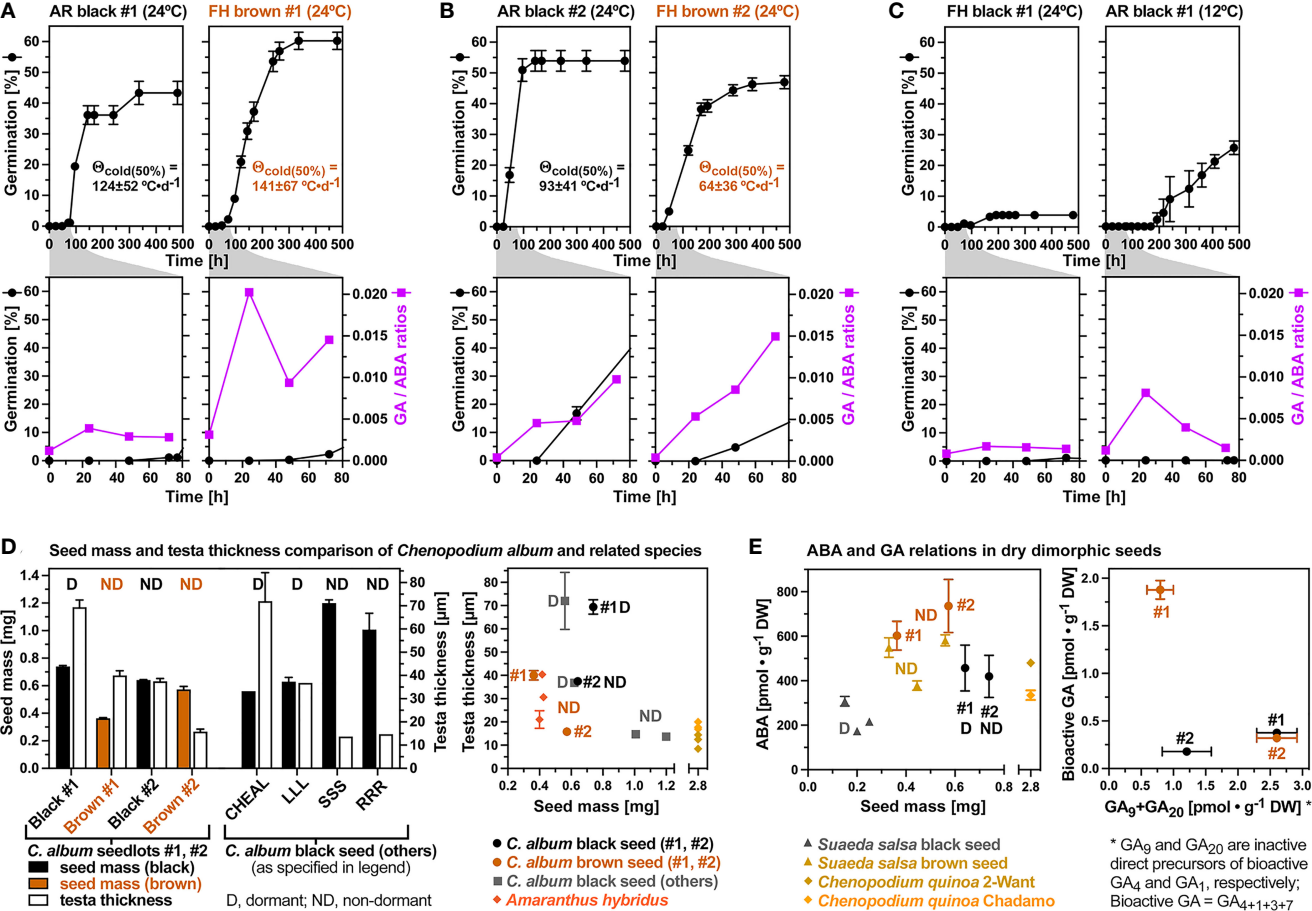
*Chenopodium album* dimorphic seed GA/ABA ratios and comparison of Amaranthaceae testa thicknesses and dry seed hormone contents. **(A)** Germination time courses and bioactive GA/ABA ratios of seedlot #1 black (after-ripened) and brown seeds imbibed at 24°C. **(B)** Germination time courses and bioactive GA/ABA ratios of seedlot #2 dimorphic seeds imbibed at 24°C. Mean ± SEM values are presented. The thermal-time constants Θ_cold(50%)_ ± SD from the population-based threshold modeling (**Supplementary Figure 1**) are indicated and those of seedlot #1 are similar to *Chenopodium quinoa* (141-152 °C•d-1 from Ceccato et al., 2015). **(C)** Germination time courses and bioactive GA/ABA ratios of seedlot #1 black (after-ripened) imbibed at 12°C and 24°C. **(D)** Comparisons of *C. album* seed mass and testa thickness of the dimorphic seeds of seedlots #1 and #2 (this work) with published work: CHEAL (Gardarin et al., 2010), and LLL, SSS, RRR (Karssen, 1970) (*left panel*). Comparisons of *C. album* seed mass and testa thickness with *Amaranthus hybridus* (Gardarin et al., 2010; Nakabayashi and Leubner-Metzger, 2021) and *C. quinoa* (Ceccato et al., 2015) (*right panel*). Note that for *C. album* seedlots #1 and #2 the D (dormant) refers to the freshly harvested mature seed with full physiological dormancy (non-deep and deep PD; nPD+dPD) for seedlot #1, whereas the ND refers to the 2/3^rd^ of non-dormant seed with nPD not induced during seed maturation. **(E)** Comparisons of ABA and GA relations in dry dimorphic seeds of *C. album* (this work), *Suaeda salsa* (Wang et al., 2015; Li et al., 2016; Xu et al., 2017), and *C. quinoa* (Ceccato et al., 2015).

In contrast to the accumulation of bioactive GA, the ABA contents of seeds declined during imbibition from contents in the dry state which were higher in brown as compared to black seeds (**Figure 4A**). This raised the question of whether biosynthesis or catabolism of ABA is regulated differently between the seed morphs and between different dormancy states. Four 9-*cis*-epoxycarotenoid dioxygenase (NCED) genes were expressed in *C. album* seeds, with transcript abundances of *CalNCED3* declining in non-dormant seeds imbibed at 24°C (**Figures 7A, B**). In FH black #1 seeds the *CalNCED3* transcript abundances increased in a transient manner (**Figure 7A**), suggesting a role in maintaining dormancy. In black and brown #2 seeds imbibed at 24°C the *CalNCED5*, *CalNCED6A* and *CalNCED6B* transcript abundances were low (**Figures 7A, B**). ABA degradation by ABA 8’-hydroxylases (CYP707A) and the PA/DPA pathway was more rapid in black and brown seeds of seedlot #2 compared to seedlot #1 (**Figure 4**). While *CalCYP707A2* transcript abundances in general declined upon imbibition at 24°C, those of *CalCYP707A1/3* and *CalCYP707A4* accumulated in the brown seeds of both seedlots (**Figures 7A, B**). Finally, the apparently similar pattern of ABA contents in cold-imbibed AR black #1 seeds (**Figure 4A**), was associated by the accumulation of transcripts of all four NCEDs and all three CYP707As (**Figure 7C**). A possible interpretation of these cold responses is that the *CalNCED* and *CalGA2ox2* transcripts accumulate in the 1/3^rd^ non-germinating seeds with deeper dormancy, while the *CalCYP707A* and *CalGA3ox* transcripts accumulate in the 2/3^rd^ of germinating (non-dormant) seeds in the population. These findings also support that a tight interaction between GA and ABA, leading to distinct GA/ABA ratios (**Figure 8**) are important which is referred to in the discussion.

## 3 Discussion

### 3.1 Roles for testa thickness and color in *C. album* dimorphic seed dormancy

Phylogenetic analyses of the internal seed morphology and associated ecophysiological seed traits led to the conclusion that heterodiaspory within the Amaranthaceae *sensu lato* evolved as a bet-hedging strategy in lineages with fast seed germination (Kadereit et al., 2017). Within the genus *Chenopodium* this however did not lead to seed heteromorphism for the very fast germinating *C. quinoa* (T_50%_ 0-2 days), but it did for the annual weed *C. album* (T_50%_ 2-7 days) with its black and brown seed morphs (this work and Yao et al., 2010). A peripheral embryo with annular shape surrounding the central perisperm (nutrient tissue) and with the outer embryo edge aligned against the inner surface of the bitegmic seed coat is considered as the ancestral morphological structure of Amaranthaceae seeds (Prego et al., 1998; Sukhorukov et al., 2015; Kadereit et al., 2017; Baskin and Baskin, 2019; Vandelook et al., 2021). We found in the two *C. album* seedlots (#1 and #2) the testa is much thicker compared to the very thin tegmen (**Figure 2**), a finding which was also made for other Amaranthaceae (Prego et al., 1998; Ceccato et al., 2015; Sukhorukov et al., 2015). The *C. album* seed morphs share this ancestral internal seed morphology trait with other *Chenopodium* species and other Amaranthaceae. Seed coat thickness of weed species is positively related to their soil bank persistence (Gardarin et al., 2010). Together with seed coat pigments (color) and other biophysical properties such as permeability the thickness determines dormancy and longevity as key seed traits (Finch-Savage and Leubner-Metzger, 2006; MacGregor et al., 2015; Song et al., 2017; Steinbrecher and Leubner-Metzger, 2017; Nakabayashi and Leubner-Metzger, 2021).

Although the brown seed morph was consistently associated with non-dormancy (ND) in *C. album* (this work and Yao et al., 2010), *Suaeda salsa* (Wang et al., 2015; Li et al., 2016; Xu et al., 2017) and other Amaranthaceae with brown and black seeds (Wang et al., 2008; Baskin et al., 2014; Bhatt and Santo, 2016; Liu et al., 2018), the black seed morph was not always dormant (**Figure 8D**). The black seed coat pigment *per se* is therefore not sufficient to confer the coat-imposed dormancy. Seed mass differences between the morphs were also not associated with this trait as the brown seed morph can be either smaller (**Figure 8D**) or larger (**Figure 8E** and Yao et al., 2010). Scarification of the dormant (D) black seeds of *C. album* seedlot #1 clearly demonstrated that this is indeed coat-imposed dormancy associated with distinct testa thickness of the two seedlots as well as the morphs within each seedlot (**Figure 8D**). A thin testa of < 30 µm was associated with the ND of black (SSS, RRR; i.e. two types of short-day conditions for mother plant growth used by Karssen, 1970) and brown (#2) *C. album* seeds, as well as with *C. quinoa* seeds which are larger compared to *C. album* (**Figure 8D**). A thick testa of up to ca. 70 µm was associated with dormant black *C. album* seeds (#1, CHEAL), but if the testa thickness is ca. 40 µm it was associated with either D black (LLL; long-day conditions for mother plant growth used by Karssen, 1970) or ND brown (#1) *C. album* seeds (**Figure 8D**). For *C. album* long-day conditions (LLL) during mother plant reproduction are known to lead to thicker testa and deeper dormancy, whereas short-day conditions (RRR) lead to thinner testa and low dormancy (Karssen, 1970). In agreement with this, the long-day conditions used for our glasshouse production of seedlot #1 led to D black seeds with thick testas of ca. 70 µm (**Figures 2B**, **8D**), while a testa thickness ca. 40 µm was observed for the ND brown #1 seeds. In addition to day length also temperature during mother plant reproduction is known to affect coat-imposed dormancy for example of *Amaranthus hybridus* (**Figure 8D** and Fernández Farnocchia et al., 2021) and of *A. thaliana* (MacGregor et al., 2015).

### 3.2 Complex hormonal regulation of Amaranthaceae seed dormancy and germination

Dormancy differences associated with distinct physicochemical seed and fruit coat properties are known to affect the flux of hormones and oxygen required for the control of germination (Hermann et al., 2007; Steinbrecher and Leubner-Metzger, 2017; Nakabayashi and Leubner-Metzger, 2021). Two *C. quinoa* accessions which differed in their dry seed ABA contents (**Figure 8E**) also differed in the amount of ABA leaching into the medium upon seed imbibition (Ceccato et al., 2015). Their dry seed ABA contents were very similar to those of black and brown *C. album* and *S. salsa* seeds (**Figure 8E**). In agreement with differences in testa permeability of the *C. album* seed morphs, imbibed black and brown seeds of seedlot #2 differed in the dose responses for GA biosynthesis inhibitors (**Figure 3A**). The dry black (ND) seeds of seedlot #2 also contained lower ABA degradation metabolites compared to the brown #2 (ND), the brown #1 (ND) and black #1 (D, AR) seeds (**Figure 4C**), suggesting that distinct oxygen permeability of the testa may have caused a distinct ABA metabolism during seed maturation. Interestingly, ABA contents in dry states were higher for the brown seed morphs compared to the black seed morphs of *C. album* and *S. salsa* (**Figure 8E** and Wang et al., 2015; Li et al., 2016; Xu et al., 2017). Dormancy in these species was therefore also not associated with higher dry seed ABA contents, and ABA degradation occurred during seed imbibition in ND and D seeds of both morphs. The rate of ABA degradation during imbibition was higher in brown compared to black seeds of *S. salsa* (Li et al., 2016), but in *C. album* the rate did not appreciably differ between the black and brown seed morphs (**Figure 4A**). The rate of ABA degradation during imbibition was however higher in *C. album* seedlot #2 as compared to seedlot #1. This suggests that ratios and interaction of ABA with dormancy-releasing hormones and distinct hormone sensitivities may be more important than ABA contents itself for the seed responses to environmental cues such as ambient temperature.

Seedlots of *C. album* (black seeds) and *C. quinoa* are known to differ in their responses to ambient temperatures and water potentials (Roman et al., 1999; Soureshjani et al., 2022). In agreement with this, *C. album* seedlots #1 and #2 differed in their temperature responses and this was mainly obvious in the sub-optimal (colder) temperature range (**Figure 1** and **Supplementary Figure S1**). The release of the nondeep physiological dormancy (increase in G_max_) during the after-ripening storage of black #1 seeds was associated with widening of the permissive window mainly towards colder temperatures. Within a seedlot the dimorphic seeds (AR black #1 seeds) differed less in their responses to cold temperatures, but they differed more in the supra-optimal (warmer) temperature range. *Chenopodium album* seeds germinate with TR and ER as two subsequent visible events which allows to assign targets for hormones and environmental cues (**Figure 1** and Karssen, 1968; Karssen, 1976a; Karssen, 1976b). We found that while ABA has ER as its sole target, cold temperatures (12°C) close to the T_base_ (8°C) delayed both TR and ER (**Figure 1B**). Light, GA, cytokinins, ethylene and nitrate were shown to act as ABA antagonists to fully release the dormancy and increase germination speed by targeting TR and ER, respectively (**Figure 1F**). Earlier work demonstrated that black seeds even in the after-ripened state required light for germination, light increased the G_max_ in a seedlot-specific manner typically to 55-85% (Karssen, 1970; Karssen, 1976a; Machabée and Saini, 1991; Altenhofen and Dekker, 2013). In agreement with this earlier work, we found that the G_max_ of AR black and brown seeds of seedlot #1 was very low in darkness and increased in the light ca. 60% (**Supplementary Figure S2**). Light is known to act *via* increasing GA biosynthesis and signaling to release dormancy and promote germination (Finch-Savage and Leubner-Metzger, 2006). Treatment of imbibed seeds with GA biosynthesis inhibitors in the light demonstrated that both morphs (black and brown) required GA biosynthesis also for germination (**Figure 3**). Furthermore, treatment with bioactive GA and fluridone caused dormancy release resulting in a G_max_ of up to ca. 70% seed germination. We conclude from these findings that 2/3^rd^ of the seed populations have either a layer of nondeep PD (nPD) which can be released by GA and AR in black seeds of seedlot #1, or have ND if this layer of nPD was not induced during the seed maturation as it was observed in brown seeds of both seedlots, and black seeds of seedlot #2. These triggered our detail investigation of the GA and ABA metabolism and raised the importance of the GA/ABA ratios in the dimorphic seeds.

### 3.3 Diversification and expression of *Chenopodium* GA and ABA metabolism genes

Comparative analysis of 5 sequenced Amaranthaceae and 9 other plant genomes (Ma et al., 2021) revealed a high proportion of expanded gene families associated with a recent whole genome duplication event in this lineage. In contrast to the gene family expansion and diversification observed in the *C. quinoa* genome, the genomes of *Beta vulgaris* and *Amaranthus cruentus* (Ma et al., 2021) were rather characterized by gene family contractions. The genus *Chenopodium* with its >150 species has a worldwide distribution. The economically important *C. quinoa* is an allotetraploid which resulted from hybridization of diploid ancestral lineages (Jarvis et al., 2017). For *C. album* the allohexaploid origin resulting from hybridization between diploid and tetraploid ancestral lineages was confirmed (Krak et al., 2016) and the evolutionary histories of Eurasian representatives reconstructed (Mandak et al., 2018). Our detailed sequence analyses of the identified *C. album* genes revealed their putative *C. quinoa* orthologs with numerous sequences (**Figures 5**, **Figure 6**, and **Supplementary Figure 7**). The phylogenetic analyses of GA and ABA metabolism genes support an extended diversification of the *Chenopodium GA20ox*, *GA3ox*, *NCED*, and *CYP707A* genes within the Amaranthaceae in a manner distinct from the *A. thaliana*. Biochemical analyses not only with the *Arabidopsis* proteins, but also with *Cucumis* proteins from other subgroups (**Figure 5**) demonstrated that they have GA-oxidase enzyme activity (Lange and Lange, 2020), suggesting that this is also the case for related diversified *Chenopodium* subgroup members. Divergence and subgroups without *Arabidopsis* genes were also observed for the GA-oxidase subfamilies in other species in phylogenetic analyses (Giacomelli et al., 2013; Huang et al., 2015; Sabir et al., 2022). The situation is similar for NCED (Arshad et al., 2021; Li et al., 2021) and CYP707A (Zheng et al., 2012). However, even the comprehensive work of Huang et al. (2015) into the divergence and adaptive evolution of the GA-oxidase genes in plants did not include any Amaranthaceae sequences. Inclusion of Amaranthaceae sequences in phylogenies is however relevant to also cover the Caryophyllales which is distinct from the Rosids and Asterids. Transcriptome analyses of *C. quinoa* seed germination (Wu et al., 2020; Hao et al., 2022) demonstrated that for several cases the corresponding orthologs were expressed during *C. quinoa* and *C. album* imbibition as detailed in **Supplementary Figure 5**.

Taken together, distinct patterns of gene expression and ABA and GA metabolites were observed for *C. album* seeds. They differed between the dimorphic seeds (black versus brown) as well as between the two accessions (#1 versus #2). We found that the 13-non-hydroxylated GA biosynthesis pathway dominates in both dimorphs and both seedlots, with GA_4_ as the major bioactive GA (**Figure 4**). The 13-non-hydroxylated GA biosynthesis pathway and GA_4_ as bioactive GA was also evident in dimorphic *Sueada salsa* seeds (Li et al., 2016), but the 13-hydroxylated pathway was not studied in this species. When dry *C. album* seeds were compared for their contents in bioactive GAs and their direct precursors (GA_9_+GA_20_) distinct strategies became evident (**Figure 8E**). Dry brown seeds of seedlot #1 had mainly bioactive GAs stored, whereas dry seeds of seedlot #2 mainly the direct precursors. Dry black seeds of seedlot #1 also mainly had the direct precursors stored, whereas dry black seeds of seedlot #2 were low in both. Together with ABA degradation this led to the observed differences in the GA/ABA ratios (**Figure 8**). For seedlot #1 the GA/ABA ratio remained very low in FH (dormant) black seeds, increased >3-fold in AR black and >22-fold in brown seeds (**Figures 8A, C**).

For seedlot #1 this change in GA/ABA ratio was not associated without major changes in the transcript abundance patterns for the key GA biosynthesis enzymes during imbibition, which is consistent with stored bioactive GAs or direct precursors in the dry seeds (**Figure 8E**). In dormant black #1 seeds the ABA biosynthesis gene *CalNCED3* was induced during imbibition suggesting a role in dormancy maintenance (**Figure 7A**). In agreement with this, for the *B. vulgaris* corresponding ABA metabolism gene upregulation in imbibed seeds during dormancy (NCEDs) and germination (CYP707As) has been reported (Hermann et al., 2007; Hourston et al., 2022). In contrast to *C. album* seedlot #1, the morphs of seedlot #2 did not appreciably differ in their GA/ABA ratio patterns which increased 5-10 fold (**Figure 8B**). This was associated with higher transcript abundance for *CalGA20ox2* and up-regulation of the *CalGA3ox1/2, CalCYP707A1/3* and *CalCYP707A4* genes especially in brown seeds of seedlot #2 (**Figure 7B**). The up-regulation of the *CalCYP707A1/3* and *CalCYP707A4* genes explains the rapid ABA degradation and steep increase in the GA/ABA ratios in the dimorphic seeds of seedlot #2 (**Figure 8B**). The importance of GA-ABA interaction was also evident upon ABA treatment of imbibed *C. quinoa* seeds which inhibited the decline in the transcript abundances observed in the control for *CqGA20ox2-like*, *CqNCED6*, *CqCYP707A2* and *CqCYP707A2-like* (**Supplementary Figure 5**).

### 3.4 Hierachic layering of dormancy release and seed dimorphism as bet-hedging strategies

Interestingly, when AR black #1 seeds were imbibed at cold temperature (12°C) the GA/ABA ratios transiently increased (**Figure 8C**) and both GA and ABA biosynthesis and degradation genes were up-regulated (**Figure 7C**). A possible interpretation for this is that the GA biosynthesis (*CalGA3ox1/2*, *CalGA3ox3/4*, *CalGA3ox1/4*) and ABA degradation (*CalCYP707A1/3*, *CalCYP707A2*, *CalCYP707A4*) genes are induced in the germinating seeds of the population as a cold stratification response. These non-dormant seeds constitute 2/3^rd^ of the population for which nondeep PD (nPD) was released by the AR storage. In contrast to this, the induction of the GA inactivation (*CalGA2ox2*) and ABA biosynthesis (*CalNCED3*, *CalNCED5*, *CalNCED6A*, *CalNCED6B*) genes could be associated with the non-germinating 1/3^rd^ of the population which has a distinct layer of deeper PD (dPD). This dPD can be released by a combination of ethylene and nitrate (**Figures 3E-G**). Machabée and Saini (1991) discovered that imbibed *C. album* seeds produce ethylene, that dormancy release of some seedlots require ethylene action, and that this occurs in a temperature-dependent manner and is further enhanced by nitrate.

Our analysis sheds new light on seed hetermorhism as a bet-hedging strategy to establish persistent seeds banks for this species. The observation that the population has a fraction with nondeep physiological dormancy (nPD) and the other deep physiological dormancy (dPD) suggest a distinct bet-hedging strategy *via* the hierarchical layering of sensitivity to environmental signals. Similar findings were made for the dormancy release in *A. thaliana* Cvi (Finch-Savage et al., 2007) in that the light requirement persisted also in the AR state and that the sensitivities to cold and nitrate differed between seedlots. The results in both species are consistent with a role for the GA/ABA balance in integrating dormancy release signals. *Arabidopsis thaliana* seeds continually adjust their dormancy status by sensing a range of environmental signals related to temporal (e.g. temperature) and spatial (e.g. light and nitrate) change that indicate conditions suitable for germination (Finch-Savage and Footitt, 2017). This apparent hierarchy and layering of dormancy depth in the dimorphic seeds of *C. album* therefore provides a bet-hedging strategy in which a fraction of the population forms a persistent seed bank. Future research will reveal how the *C. album* seed morphs differ in dormancy cycling behaviour.

## 4 Conclusion

Using *C. album* as weed model system, we conducted a comparative morphological and molecular analysis of its dimorphic seeds (black and brown) with two seedlots (#1 and #2) of distinct origin. This analysis revealed distinct patterns of GA and ABA metabolism and related gene expression between the seedlots and morphs. In non-dormant brown seeds the testa thickness was half of the corresponding black seeds. Freshly harvested black seeds of seedlot #1 had a thick testa and testa-imposed dormancy which can be released by scarification at the micropylar end. After-ripening or GA treatment caused dormancy release in black #1 seeds and released the nondeep PD (nPD) of 2/3^rd^ of the seeds in the population. Black seeds of seedlot #2 had thinner testas, their thickness was half of black #1 and equal to brown #2, and brown seeds of seedlot #2 had an even thinner testa. The 2/3^rd^ of the seedlot #2 black seeds were non-dormant in the FH state, suggesting that nPD was not induced in this population. Earlier work with black *C. album* seeds concluded that depending on the photoperiod during reproduction two distinct dormancy layers can be induced (Karssen, 1970). We speculate that the first one is nPD, which is not induced in black seeds of seedlot #2, but is induced in black seed of seedlot #1 in associated with a thicker testa and AR and GA responsiveness to release the nPD. This first dormancy layer is mechanistically associated with the investigated GA and ABA as summarized in **Figure 8**. It explains the germination behavior of 2/3^rd^ of the seeds in the populations. We propose that the 1/3^rd^ non-germinating black seeds in the populations have a second deeper dormancy layer (dPD) imposed which can be released by a combination of ethylene and nitrate (**Figures 3E-G** and Machabée and Saini, 1991). Our future work with the dimorphic seeds of *C. album* seedlots will address this second layer of seed dormancy including with transcriptome analysis. Understanding and integrating complex mechanisms of seed dormancy dynamics with realistic ecophysiological simulations are essential for more sustainable weed management strategies (Baskin and Baskin, 2006; Batlla et al., 2020; Nakabayashi and Leubner-Metzger, 2021).

## 5 Materials and methods

### 5.1 Plant material

Seeds of *Chenopodium album* L. accession #1 were initially obtained from Syngenta Ltd. in 2015 (field sample PS-9522, Brechin, Angus, UK, 56.73 N -2.66 W) and stored in hermetically sealed containers containing silica gel at room temperature. These seeds were re-propagated at Royal Holloway’s in-house glasshouse and harvested at maturity (seedlot #1). Seeds were sown in 2/3^rd^ Levington F2+S seed compost and 1/3^rd^ perlite (Evergreen Garden Care Ltd., Frimley, UK) containing 0.4 g/L of Exemptor^®^ insecticide (Bayer CropScience Limited, Cambridge, UK) and watered daily. Soil and plants were sprayed with Provado^®^ Ultimate Bug Killer (Bayer) biweekly to limit pest growth. Growth conditions set in the glasshouse were a 16-h photoperiod, and day/night temperature 20°C/18°C ± 2°C. *Chenopodium album* accession #2 was obtained from direct collection of seeds during autumn 2020 outside Egham’s leisure center (51.43 N 0.54 W), Surrey, UK (seedlot #2). Freshly harvested (FH) seeds were dried for 1 week at 14% relative humidity (RH) and seedlots cleaned using a gravity separator. Brown seeds were manually separated from the mainly black seeds of the populations. After-ripened (AR) seeds were obtained from FH seeds by after-ripening dry storage (20°C, 33% RH) for 11 weeks. This duration of AR storage is known to be sufficient to fully release a first layer of *C. album* primary seed dormancy; a second dormancy layer (if present) is not released by the AR storage (Karssen, 1970; Machabée and Saini, 1991).

### 5.2 Seed germination assays

Seed germination assays were performed using 3 replicates of 30 seeds in 60-mm Petri dishes. Seeds were imbibed with 2 ml of autoclaved deionized water on two layers of filter papers (MN713; Macherey-Nagel, Dueren, Germany) and sealed using parafilm. The assays were performed in MLR-352 Versatile Environmental Test Chambers (Panasonic, Bracknell, UK) under constant white light (100 µmol m^-2^ s ^-1^) at 24°C. Temperature response assays were performed on a GRD1-LH temperature gradient plate device (Grant Instruments Ltd., Cambridge, UK). Dose-response germination assays were performed using gibberellin A_4+7_ (GA_4+7_; Duchefa Biochemie, Haarlem, The Netherlands), *cis,trans*-S(+)-ABA (ABA; Duchefa), flurprimidol (Sigma, St Louis, MO, USA), fluridone (FLU; Duchefa). Compounds were supplied from liquid stocks dissolved in dimethyl sulfoxide (DMSO) stored at -20°C. All dilutions were adjusted to the same DMSO concentration of 0.1% (v/v) and all experiments included a 0.1% (v/v) DMSO control as well as a water control. Germination was recorded daily using a stereomicroscope and scored as completed upon visible radicle emergence through the testa and endosperm. In experiments where testa rupture (TR) and endosperm rupture (ER) were scored separately, multiple daily recordings were taken.

### 5.3 Microscopy and morphological analyses

Images of dry seed cross sections were taken using a DCF480 digital camera attached to a MZ 12.5 stereomicroscope (Leica, Wetzlar, Germany). Seed coat thickness represents testa thickness (the tegmen is very thin) and was measured using IMAGEJ (version 1.52i; National Institute of Health, Bethesda, MD, USA). For higher-resolution microscopy images, five seeds were punctured at the micropylar end using a sterile needle and 5 were left as controls. These seeds were imbibed for 24 hrs inside a 60-mm Ppetri dish. The seeds were then fixed in 4% paraformaldehyde in PEM buffer (0.1 M 1, 4-piperazinediethanesulfonic acid (pH 6.9), 2 mM triethylene glycol diamine tetraacetic acid and 1 mM magnesium sulfate) (Zeller, 2001) before being vacuumed for 5 min, sealed with vacuum off for 15 min, then slowly release before repeating the vacuum process 3 times. Seeds were then washed with PBS buffer (138 mM NaCl; 2.7 mM KCl, pH 7.4) for two 30 min periods before being subject to a dehydration through an ethanol gradient. Then the seeds undergo imbedding with methyl methylacrylate resin from the Technovit 7100 protocol (Kulzer Technik, Wehrheim, Germany), modified by adding an ethanol: resin gradient to improve uptake into starchy tissues. The polymerization blocking was next performed following the Technovit histology protocol (Matsushima, 2014). Sections of 5 µm thickness cuts were sliced from the blocks using a HM 355S microtome (Thermo Scientific, Loughborough, UK), mounted on slides, stained in 1% safranin solution for 5 minutes, counterstained in 0.1% methylene blue 15 minutes and analyzed using a Nikon ECLIPSE Ni-E microscope (Nikon, Amstelveen, The Netherlands).

### 5.4 Hormone extraction and quantification

Five biological replicates of 10 mg black and brown *C. album* seeds in the dry state (0h) and imbibed for 24h, 48h and 72h at either 24°C or 12°C were used. Seeds were then snap frozen in liquid nitrogen and lyophilized for 2 days using a freeze dryer LYOVAC GT 2 (SEIB Industrie, Gothenburg, Sweden) and metabolites were extracted. The levels of GA and ABA pathway metabolites, physiologically active or non-active, were quantified by UHPLC-MS/MS as described previously (Turečková et al., 2009; Urbanova et al., 2013; Walker et al., 2021; Hourston et al., 2022).

### 5.5 PCR cloning of *C. album* sequences and phylogenetic analyses

To identify *Chenopodium* sequences of GA and ABA metabolism genes expressed in seeds we mined the *Chenopodium quinoa* genome (Jarvis et al., 2017) and conducted BLAST analyses with the *A. thaliana* and *C. quinoa* sequences *via* TAIR and Phytozome (Goodstein et al., 2012) as presented in detail in **Supplementary Figure 4**. The combined information of the BLAST analyses and subgroups in phylogenetic trees was used to design primers based on *C. quinoa* sequences (**Supplementary Table 1**) targeting the sub-groups for the PCR cloning of *C. album* sequences expressed in seeds. PCR products were sent to Eurofins Genomics (Ebersberg, Germany) for Sanger sequencing. A total of 17 sequences of various lengths were obtained (**Supplementary Table 1**), analyzed by BLAST (**Supplementary Figure 6**) and deposited in the GenBank as BankIt submissions 2668556 and 2664064 which provided the allocated accession numbers (**Supplementary Table 1**). Phylogenetic analysis of Amaranthaceae, Brassicales and Cucurbitales GA oxidases (**Figure 5**, **Supplementary Figure 7**), NCEDs and CYP707As (**Figure 6**) with known and putative amino acid sequences aligned using ClustalW (BLOSUM cost matrix, Gap open cost 10, Gap extend cost 0.1) and Neighbor-Joining trees (Saitou and Nei, 1987) were built using Geneious 8.1.9 Tree Builder (Geneious, San Diego, CA, USA) using Jukes-Cantor distance. Consensus support (minimum 20%) was determined using bootstrap (1000).

### 5.6 Gene expression analysis *via* RT-qPCR

Four replicate samples of 20 mg black and brown *C. album* seeds were taken from seedlots #1 or #2 in the dry state (0h) or imbibed for 24h, 48h and 72h at either 24°C or 12°C, as indicated. Samples were homogenized in liquid nitrogen using a pestle and mortar, before addition of 1 ml extraction buffer containing 2% (w/v) hexadecyltrimethyl-ammonium bromide (CTAB), 2% (w/v) polyvinyl pyrrolidone MW 4000 (PVP), 100 mM Tris-HCl (pH 8), 25 mM ethylenediaminetetraacetic acid (EDTA, pH 8), 2 M NaCl and 2% (w/v) β-mercaptoethanol. Using this, the RNA extraction was performed as described in Graeber et al. (2011) with their listed modifications. RNA quantification was performed using a NanoDrop™ spectrophotometer (ND-1000, Thermo Scientific™, Delaware, USA) and agarose gel electrophoresis was used to confirm the presence of RNA. RNA integrity was assessed by using Aligent Bioanalyzer™ (Aligent Technologies), the three replicates with the highest RIN values for each treatment were selected for cDNA synthesis. One µg of RNA was used for cDNA synthesis using the Invitrogen™ SuperScript™ III First-Strand synthesis kit (Thermo Scientific, Loughborough, UK) following the manufacturer’s protocol.

The *C. album* GAox, NCED and CYP707A sequences plus reference genes (*ACT7-like*, *CDC27B*, *PUB33-like*) were used to design specific primers for the RT-qPCR analysis (**Supplementary Table 2**). A cDNA pool template (combination of all samples, black, brown, dormant, non-dormant, dry and imbibed) was used to verify the primers (**Supplementary Table 2**). Subsequently, cDNAs from each of the three replicates per sampling point were used to conduct the qPCR analysis. RT-qPCR reactions were performed using a CFX96 Real-Time PCR Detection System (Bio-Rad, Hercules, CA, USA) and was conducted as detailed in Graeber et al. (2011). After baseline correction was removed, C_q_ values and PCR efficiencies were calculated using PCR miner of the raw fluorescence data values (Zhao and Fernald, 2005). Reference gene suitability was assessed by the geNorm software (https://genorm.cmgg.be/), which identified two reference genes (*ACT7-like*, *CDC27B*) for seedlot #1 samples and two reference genes (*PUB33-like*, *CDC27B*) for seedlot #2 samples as the most stable and uniformly expressed across all treatments. Candidate genes were normalized against the geometric mean of the expression levels of the two reference genes as described (Graeber et al., 2011). C_t_ and normalized relative gene expression levels were plotted on a logarithmic scale using a non-linear fit on GraphPad Prism v7 (GraphPad Software, San Diego, CA, USA).

### 5.7 Thermal-time modeling and statistical analysis

The cardinal temperatures permissible for germination including base temperature (T_base_), optimal temperature (T_opt_) and ceiling temperature (T_c_), and the thermal time constants Θ were identified by population-based threshold modelling for thermal time (“heat sums”) (Roman et al., 1999; Bradford, 2002; Steckel et al., 2004; Finch-Savage and Leubner-Metzger, 2006; Batlla and Benech-Arnold, 2015; Soureshjani et al., 2022). To achieve this, the germination rates GR, i.e. the inverse of time to germination for a given percentage of the population (1/t_g_), this was plotted against temperature (**Supplementary Figure 1**). Linear regression analysis with GraphPad Prism v7 was used to calculate regression lines in the sub-optimal (colder) and supra-optimal (warmer) temperature regions. Their intercepts were used to estimate T_b_, T_o_ and T_c_, respectively (**Supplementary Figure 1**). Their slopes provide the thermal time constants to germination (Θ_cold(g)_ and Θ_warm(g)_). At sub-optimal temperatures, Θ_cold(g)_ varies among individual seed fractions in the population and is normal distributed around Θ_cold(50%)_. At supra-optimal temperatures, T_c(g)_ is normal distributed around T_c(50%)_ (**Supplementary Figure 1**). Statistical analyses of obtained germination curves were by using a non-linear fit on GraphPad Prism v7 (GraphPad Software, San Diego, CA, USA). This program was also used to calculate mean ± SD and SEM values.

## Data availability statement

The datasets presented in this study can be found on Royal Holloways figshare: https://doi.org/10.17637/rh.21829842.v1.

## Author contributions

EL, AS, KN, MS and GL-M conceptualized and designed the research. EL, MP, VT, and DT performed experiments. EL, MP, VT, DT, KN and GL-M analyzed and interpreted data. EL and GL-M wrote the original manuscript draft. All authors complemented on the draft and were thereby involved in shaping the manuscript. All authors contributed to the article and approved the submitted version.
