# Supplementary material for: Distinct hormonal and morphological control of dormancy and germination in Chenopodium album dimorphic seeds
Source: Front Plant Sci. 2023 Mar 30;14:1156794. doi: 10.3389/fpls.2023.1156794 (PMC10098365; doi:10.3389/fpls.2023.1156794)
Supplement: Supplementary file 1 [file DataSheet_1.pdf]

Supplementary Material

Distinct Hormonal and Morphological Control of Dormancy and Germination in *Chenopodium album* Dimorphic Seeds

Eddison Loades, Marta Pérez, Veronika Turečková, Danuše Tarkowská, Miroslav Strnad, Anne Seville, Kazumi Nakabayashi, Gerhard Leubner-Metzger

Frontiers in Plant Science 2023

Research Topic "Molecular Basis of Seed Germination and Dormancy"

Supplementary Figure 1 | Population-based thermal-time threshold modelling of *C. album* dimorphic seeds.

Supplementary Figure 2 | Effect of light and hormones on the germination of *C. album* dimorphic seeds.

Supplementary Figure 3 | Gibberellin metabolite contents in dry and imbibed *C. album* dimorphic seeds.

Supplementary Figure 4 | BLAST analysis of *C. quinoa* GA and ABA metabolism gene transcripts.

Supplementary Figure 5 | Transcript expression patterns of GA and ABA metabolism genes during *C. quinoa* seed germination.

Supplementary Figure 6 | BLAST analysis of identified sequences of *C. album* GA and ABA metabolism genes.

Supplementary Table 1 | Primer sequences used for PCR cloning of *C. album* sequences.

Supplementary Table 2 | Primer sequences used for the *C. album* RT-qPCR analysis.

Copyright © 2023 Loades, Pérez, Turečková, Tarkowská, Strnad, Seville, Nakabayashi, Leubner-Metzger. This is an open-access article distributed under the terms of the Creative Commons Attribution License (CC BY). The use, distribution or reproduction in other forums is permitted, provided the original author(s) and the copyright owner(s) are credited and that the original publication in this journal is cited, in accordance with accepted academic practice. No use, distribution or reproduction is permitted which does not comply with these terms.

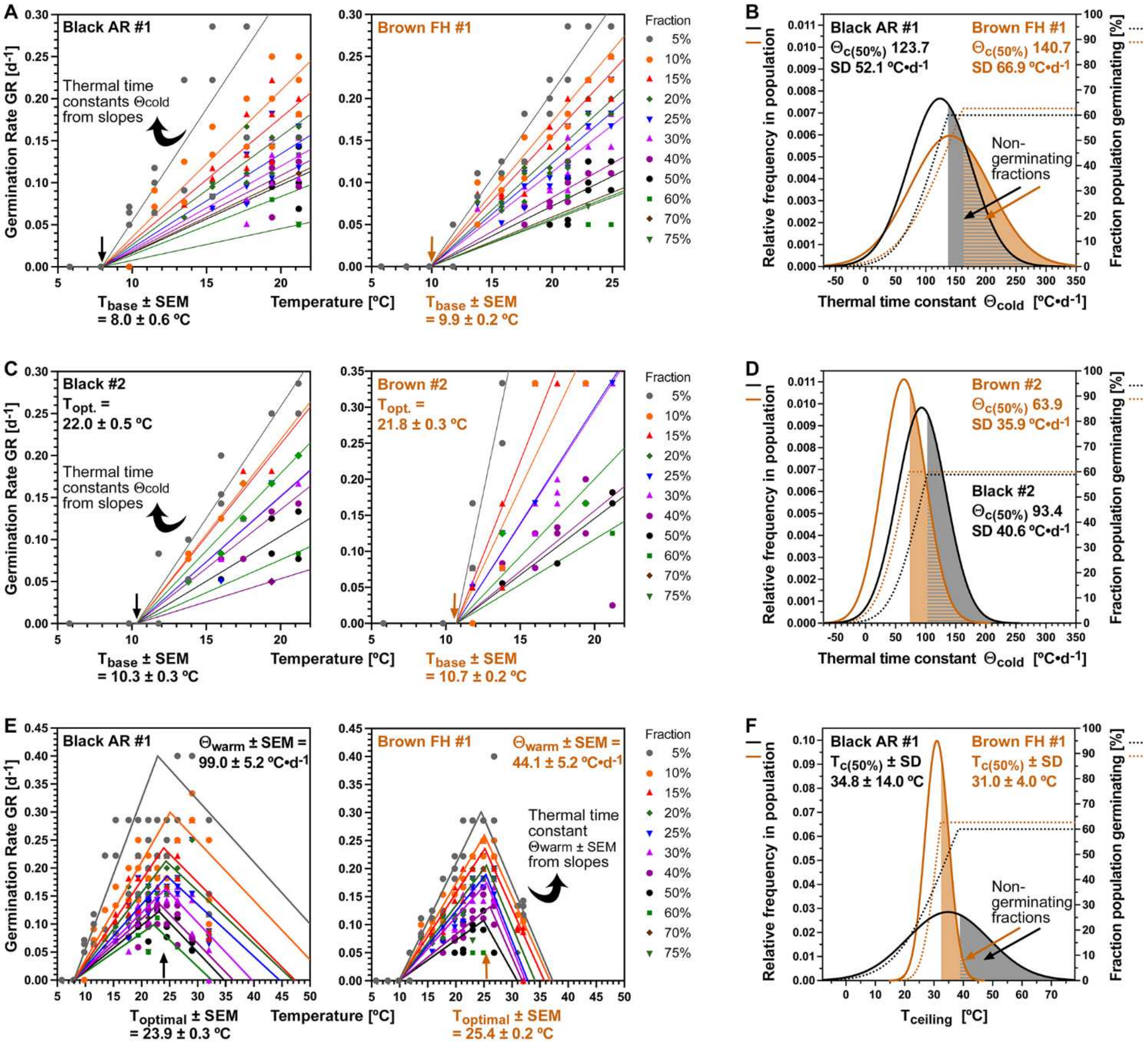

Supplementary Figure 1 | Population-based thermal-time threshold modeling of *Chenopodium album* dimorphic seeds. (A) Germination rates (GR_g), i.e. the inverse of time to germination for a given percentage of the population ($1/t_g$), of after-ripened (AR) black and freshly harvested (FH) brown seeds of seedlot #1 were calculated from the germination kinetics (Figure 1D), and plotted against the temperatures. Single-plate values for the sub-optimal (colder) temperature range for the different percentage fractions are presented and were used in linear regression analysis with GraphPad Prism v7 to calculate regression lines and to estimate base temperatures (T_{base}, derived from intercepts with x-axis) and thermal-time constants ($\Theta_{cold(g)}$; derived from different slopes). **(B)** Frequency distribution of $\Theta_{cold(g)}$ and in the seed populations calculated from the standard deviations (SD) of the thermal-time constant at 50% germination ($\Theta_{cold(50\%)}$); the non-germinating seed fraction populations are indicated. **(C,D)** The corresponding analysis for seedlot #2. **(E)** Full thermal-time analysis of seedlot #1 including in the supra-optimal (warmer) temperature range, estimated optimal temperatures ($T_{opt.}$) and thermal-time constants Θ_{warm} ± SEM (slopes are similar in the supra-optimal temperature range). **(F)** Estimated ceiling temperatures (T_c) which are normal distributed around $T_{c(50\%)}$.

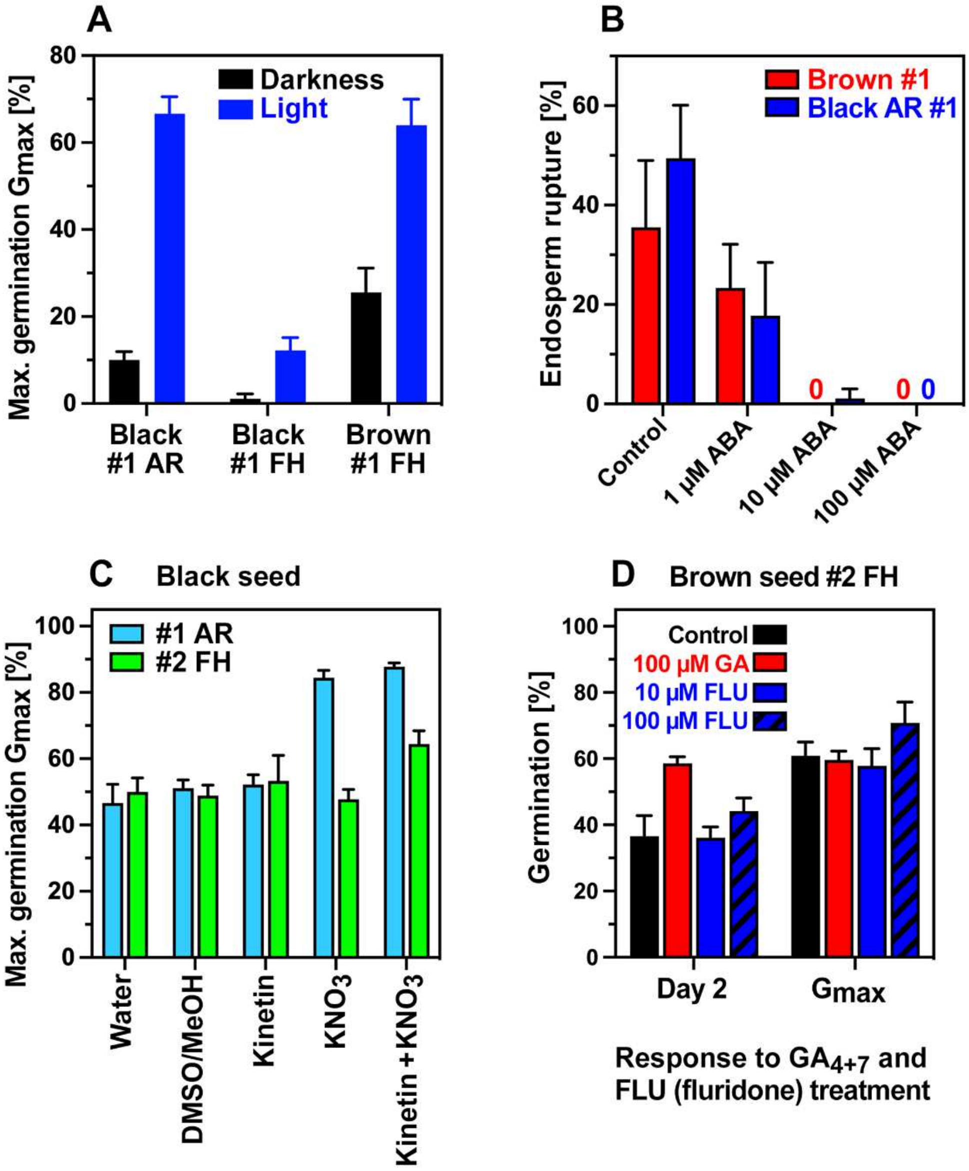

Supplementary Figure 2 | Effect of light and hormones on the germination of *Chenopodium album* dimorphic seeds. **(A)** Effects of continuous darkness and white light (100 µmol m^{-2} s^{-1}) on the maximal germination percentages (G_{max}) of after-ripened (AR) and freshly harvested (FH) mature seeds of seedlot #1 imbibed at 24°C for 20 days. **(B)** Endosperm rupture of FH brown and AR black seeds imbibed for 10 days in the absence or presence of abscisic acid (ABA) at the concentrations indicated. **(C)** The effect of kinetin (40 µM), KNO_3 (10 mM), DMSO (0.1%) and MeOH (0.1%) on the G_{max} of imbibed black seeds at 24°C for 20 days. **(D)** The effect of GA_{4+7} (GA) and fluridone on the germination of brown seeds of seedlot #2. Mean ± SEM values are presented.

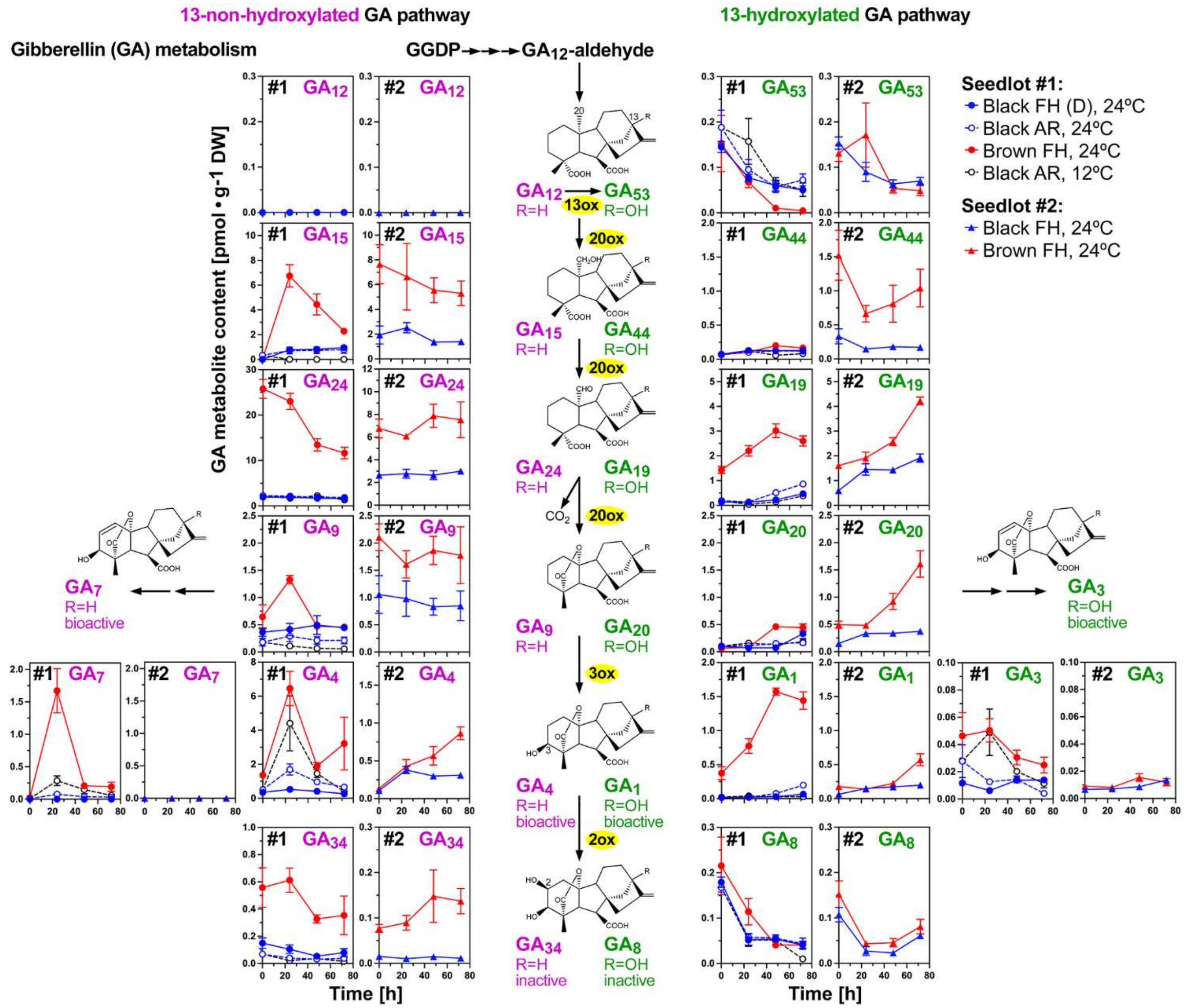

Supplementary Figure 3 | Gibberellin metabolite contents in dry and imbibed *Chenopodium album* dimorphic seeds. Temporal GA metabolite content comparison in seedlots #1 and #2 at 24ºC, and AR #1 black seeds at 12°C; Mean ± SEM values are presented. The 13-non-hydroxylated and the 13-hydroxylated GA biosynthesis and catabolic pathways with key enzymes (GA 20- oxidases, GA 3-oxidases, GA2-oxidases) are indicated. For further details, methods and references see the main text.

A BLAST analysis of GA20ox genes to identify *Chenopodium quinoa* sequences as templates for primer design

Arabidopsis thaliana gene identifier	Gene	Protein length (aa)	BlastN vs predicted (Phytozome website)	Score	E-value	BlastX vs Chenopodium quinoa transcripts (Phytozome website)	Score	E-value
AT4G25420	GA20ox1	377	jgi:C_Quinoa_Scaffold_1783_392 Cquinoa|392|v1.0	207	1.0E-50	jgi:36311734 C_quinoa_v1_0|392|AUR62033388-RA|AUR62033388-RA|GA20...	494	9.0E-174
			jgi:C_Quinoa_Scaffold_4250_392 Cquinoa|392|v1.0	204	1.0E-49	jgi:36288619 C_quinoa_v1_0|392|AUR62016064-RA|AUR62016064-RA|GA20...	491	1.0E-172
			jgi:C_Quinoa_Scaffold_2608_392 Cquinoa|392|v1.0	204	1.0E-49	jgi:36318333 C_quinoa_v1_0|392|AUR62021271-RA|AUR62021271-RA|GA20...	489	6.0E-172
			jgi:C_Quinoa_Scaffold_3256_392 Cquinoa|392|v1.0	194	7.0E-47	jgi:36292046 C_quinoa_v1_0|392|AUR62013454-RA|AUR62013454-RA|GA20...	373	7.0E-127
			jgi:C_Quinoa_Scaffold_3429_392 Cquinoa|392|v1.0	105	3.0E-20	jgi:36319174 C_quinoa_v1_0|392|AUR62004269-RA|AUR62004269-RA|GA20...	369	3.0E-126
						jgi:36285550 C_quinoa_v1_0|392|AUR62007685-RA|AUR62007685-RA|GA20...	333	1.0E-111
						jgi:36298246 C_quinoa_v1_0|392|AUR62000497-RA|AUR62000497-RA|GA20...	292	7.0E-96
						jgi:36302756 C_quinoa_v1_0|392|AUR62006843-RA|AUR62006843-RA|GA20...	292	1.0E-95
						jgi:36308110 C_quinoa_v1_0|392|AUR62011417-RA|AUR62011417-RA|SRG1...	196	2.0E-58
AT5G51810	GA20ox2	378	jgi:C_Quinoa_Scaffold_4250_392 Cquinoa|392|v1.0	199	2.0E-48	jgi:36318333 C_quinoa_v1_0|392|AUR62021271-RA|AUR62021271-RA|GA20...	473	2.0E-165
			jgi:C_Quinoa_Scaffold_2608_392 Cquinoa|392|v1.0	199	2.0E-48	jgi:36288619 C_quinoa_v1_0|392|AUR62016064-RA|AUR62016064-RA|GA20...	471	9.0E-165
			jgi:C_Quinoa_Scaffold_1783_392 Cquinoa|392|v1.0	184	1.0E-43	jgi:36311734 C_quinoa_v1_0|392|AUR62033388-RA|AUR62033388-RA|GA20...	461	8.0E-161
			jgi:C_Quinoa_Scaffold_3256_392 Cquinoa|392|v1.0	160	1.0E-36	jgi:36319174 C_quinoa_v1_0|392|AUR62004269-RA|AUR62004269-RA|GA20...	358	6.0E-122
			jgi:C_Quinoa_Scaffold_2088_392 Cquinoa|392|v1.0	123	3.0E-25	jgi:36292046 C_quinoa_v1_0|392|AUR62013454-RA|AUR62013454-RA|GA20...	356	6.0E-120
			jgi:C_Quinoa_Scaffold_3429_392 Cquinoa|392|v1.0	118	4.0E-24	jgi:36285550 C_quinoa_v1_0|392|AUR62007685-RA|AUR62007685-RA|GA20...	317	5.0E-105
			jgi:C_Quinoa_Scaffold_1611_392 Cquinoa|392|v1.0	109	2.0E-21	jgi:36302756 C_quinoa_v1_0|392|AUR62006843-RA|AUR62006843-RA|GA20...	274	2.0E-88
						jgi:36298246 C_quinoa_v1_0|392|AUR62000497-RA|AUR62000497-RA|GA20...	269	2.0E-86
						jgi:36308110 C_quinoa_v1_0|392|AUR62011417-RA|AUR62011417-RA|SRG1...	184	1.0E-53
AT5G07200	GA20ox3	380	jgi:C_Quinoa_Scaffold_1783_392 Cquinoa|392|v1.0	157	2.0E-35	jgi:36311734 C_quinoa_v1_0|392|AUR62033388-RA|AUR62033388-RA|GA20...	489	2.0E-171
			jgi:C_Quinoa_Scaffold_4250_392 Cquinoa|392|v1.0	149	3.0E-33	jgi:36318333 C_quinoa_v1_0|392|AUR62021271-RA|AUR62021271-RA|GA20...	488	8.0E-171
			jgi:C_Quinoa_Scaffold_2608_392 Cquinoa|392|v1.0	145	3.0E-32	jgi:36288619 C_quinoa_v1_0|392|AUR62016064-RA|AUR62016064-RA|GA20...	487	2.0E-170
			jgi:C_Quinoa_Scaffold_3256_392 Cquinoa|392|v1.0	143	1.0E-31	jgi:36292046 C_quinoa_v1_0|392|AUR62013454-RA|AUR62013454-RA|GA20...	357	1.0E-119
			jgi:C_Quinoa_Scaffold_2088_392 Cquinoa|392|v1.0	115	6.0E-23	jgi:36319174 C_quinoa_v1_0|392|AUR62004269-RA|AUR62004269-RA|GA20...	347	7.0E-117
			jgi:C_Quinoa_Scaffold_1611_392 Cquinoa|392|v1.0	100	4.0E-18	jgi:36285550 C_quinoa_v1_0|392|AUR62007685-RA|AUR62007685-RA|GA20...	316	3.0E-104
						jgi:36302756 C_quinoa_v1_0|392|AUR62006843-RA|AUR62006843-RA|GA20...	293	1.0E-95
						jgi:36298246 C_quinoa_v1_0|392|AUR62000497-RA|AUR62000497-RA|GA20...	293	2.0E-95
						jgi:36280783 C_quinoa_v1_0|392|AUR62012258-RA|AUR62012258-RA|ANS:...	180	7.0E-52
AT1G60980	GA20ox4	385	jgi:C_Quinoa_Scaffold_1783_392 Cquinoa|392|v1.0	154	4.0E-35	jgi:36311734 C_quinoa_v1_0|392|AUR62033388-RA|AUR62033388-RA|GA20...	468	4.0E-165
			jgi:C_Quinoa_Scaffold_2608_392 Cquinoa|392|v1.0	148	7.0E-33	jgi:36288619 C_quinoa_v1_0|392|AUR62016064-RA|AUR62016064-RA|GA20...	462	7.0E-163
			jgi:C_Quinoa_Scaffold_3256_392 Cquinoa|392|v1.0	145	2.0E-32	jgi:36318333 C_quinoa_v1_0|392|AUR62021271-RA|AUR62021271-RA|GA20...	459	1.0E-161
						jgi:36292046 C_quinoa_v1_0|392|AUR62013454-RA|AUR62013454-RA|GA20...	348	1.0E-118
						jgi:36319174 C_quinoa_v1_0|392|AUR62004269-RA|AUR62004269-RA|GA20...	338	9.0E-116
						jgi:36285550 C_quinoa_v1_0|392|AUR62007685-RA|AUR62007685-RA|GA20...	313	4.0E-105
						jgi:36298246 C_quinoa_v1_0|392|AUR62000497-RA|AUR62000497-RA|GA20...	276	4.0E-91

plus direct search for *C. quinoa* sequences (Phytozome)

Identified potential *C. quinoa* GA20ox sequences → **Verification of *C. quinoa* GA20ox genes and transcript sequences**

Phytozome Chenopodium quinoa gene ID	Phytozome Chenopodium quinoa transcript	BLAST back to TAIR (BlastX using Chenopodium quinoa phytozome cds)	Score	E-value	DEG in Chenopodium quinoa seed transcriptome	Subgroup and name in phylogeny (Figure 6A)
AUR62007685	AUR62007685-RA	AT4G25420.1 | Symbols: ATGA20OX1, AT2301, GA5, GA20OX1 | ARABIDOP...	333	5.0E-113	no	A1 - CqGA20ox1-like
		AT5G51810.1 | Symbols: AT2353, ATGA20OX2, GA20OX2 | gibberellin 2...	332	1.0E-112		
		AT5G07200.1 | Symbols: ATGA20OX3, YAP169, GA20OX3 | gibberellin 2...	317	2.0E-106		
		AT1G60980.1 | Symbols: GA20OX4, ATGA20OX4 | gibberellin 20-oxidas...	313	4.0E-105		
		AT1G44090.1 | Symbols: GA20OX5, ATGA20OX5 | ARABIDOPSIS THALIANA ...	297	9.0E-99		
		AT4G25420.2 | Symbols: ATGA20OX1, AT2301, GA5, GA20OX1 | ARABIDOP...	226	3.0E-72		
		AT5G51810.2 | Symbols: AT2353, ATGA20OX2, GA20OX2 | gibberellin 2...	218	7.0E-69		
AUR62013454	AUR62013454-RA	AT4G25420.1 | Symbols: ATGA20OX1, AT2301, GA5, GA20OX1 | ARABIDOP...	372	4.0E-128	yes (Figure S5)	A1 - CqGA20ox1-like
		AT5G51810.1 | Symbols: AT2353, ATGA20OX2, GA20OX2 | gibberellin 2...	371	1.0E-127		
		AT5G07200.1 | Symbols: ATGA20OX3, YAP169, GA20OX3 | gibberellin 2...	356	2.0E-121		
		AT1G60980.1 | Symbols: GA20OX4, ATGA20OX4 | gibberellin 20-oxidas...	348	2.0E-118		
		AT1G44090.1 | Symbols: GA20OX5, ATGA20OX5 | ARABIDOPSIS THALIANA ...	331	1.0E-111		
		AT4G25420.2 | Symbols: ATGA20OX1, AT2301, GA5, GA20OX1 | ARABIDOP...	261	1.0E-85		
		AT5G51810.2 | Symbols: AT2353, ATGA20OX2, GA20OX2 | gibberellin 2...	251	1.0E-81		
AUR62006843	AUR62006843-RA	AT5G07200.1 | Symbols: ATGA20OX3, YAP169, GA20OX3 | gibberellin 2...	293	1.0E-97	yes (Figure S5)	A2 - CqGA20ox1-like
		AT4G25420.1 | Symbols: ATGA20OX1, AT2301, GA5, GA20OX1 | ARABIDOP...	292	2.0E-97		
		AT5G51810.1 | Symbols: AT2353, ATGA20OX2, GA20OX2 | gibberellin 2...	290	1.0E-96		
		AT1G60980.1 | Symbols: GA20OX4, ATGA20OX4 | gibberellin 20-oxidas...	277	3.0E-91		
		AT1G44090.1 | Symbols: GA20OX5, ATGA20OX5 | ARABIDOPSIS THALIANA ...	275	2.0E-90		
		AT5G51810.2 | Symbols: AT2353, ATGA20OX2, GA20OX2 | gibberellin 2...	186	5.0E-57		
		AT4G25420.2 | Symbols: ATGA20OX1, AT2301, GA5, GA20OX1 | ARABIDOP...	182	2.0E-55		
AUR62000497	AUR62000497-RA	AT5G07200.1 | Symbols: ATGA20OX3, YAP169, GA20OX3 | gibberellin 2...	293	1.0E-97	no	A2 - CqGA20ox1-like
		AT4G25420.1 | Symbols: ATGA20OX1, AT2301, GA5, GA20OX1 | ARABIDOP...	292	2.0E-97		
		AT5G51810.1 | Symbols: AT2353, ATGA20OX2, GA20OX2 | gibberellin 2...	290	1.0E-96		
		AT1G60980.1 | Symbols: GA20OX4, ATGA20OX4 | gibberellin 20-oxidas...	277	3.0E-91		
		AT1G44090.1 | Symbols: GA20OX5, ATGA20OX5 | ARABIDOPSIS THALIANA ...	275	2.0E-90		
		AT5G51810.2 | Symbols: AT2353, ATGA20OX2, GA20OX2 | gibberellin 2...	186	5.0E-57		
AUR62033388	AUR62033388-RA	AT4G25420.1 | Symbols: ATGA20OX1, AT2301, GA5, GA20OX1 | ARABIDOP...	493	4.0E-175	no	B1 - CqGA20ox1-like
		AT5G07200.1 | Symbols: ATGA20OX3, YAP169, GA20OX3 | gibberellin 2...	487	8.0E-173		
		AT5G51810.1 | Symbols: AT2353, ATGA20OX2, GA20OX2 | gibberellin 2...	480	6.0E-170		
		AT1G60980.1 | Symbols: GA20OX4, ATGA20OX4 | gibberellin 20-oxidas...	467	7.0E-165		
		AT4G25420.2 | Symbols: ATGA20OX1, AT2301, GA5, GA20OX1 | ARABIDOP...	369	2.0E-127		
		AT5G51810.2 | Symbols: AT2353, ATGA20OX2, GA20OX2 | gibberellin 2...	362	6.0E-125		
		AT1G44090.1 | Symbols: GA20OX5, ATGA20OX5 | ARABIDOPSIS THALIANA ...	342	2.0E-115		
AUR62004269	AUR62004269-RA	AT4G25420.2 | Symbols: ATGA20OX1, AT2301, GA5, GA20OX1 | ARABIDOP...	367	1.0E-128	no	B1 - CqGA20ox1-like
		AT4G25420.1 | Symbols: ATGA20OX1, AT2301, GA5, GA20OX1 | ARABIDOP...	369	7.0E-128		
		AT5G51810.2 | Symbols: AT2353, ATGA20OX2, GA20OX2 | gibberellin 2...	358	1.0E-124		
		AT5G51810.1 | Symbols: AT2353, ATGA20OX2, GA20OX2 | gibberellin 2...	357	2.0E-123		
		AT5G07200.1 | Symbols: ATGA20OX3, YAP169, GA20OX3 | gibberellin 2...	347	3.0E-119		
		AT1G60980.1 | Symbols: GA20OX4, ATGA20OX4 | gibberellin 20-oxidas...	338	4.0E-116		
		AT1G44090.1 | Symbols: GA20OX5, ATGA20OX5 | ARABIDOPSIS THALIANA ...	239	4.0E-77		
AUR62016064	AUR62016064-RA	AT5G51810.1 | Symbols: AT2353, ATGA20OX2, GA20OX2 | gibberellin 2...	493	3.0E-175	yes (Figure S5)	B2 - CqGA20ox2-like
		AT4G25420.1 | Symbols: ATGA20OX1, AT2301, GA5, GA20OX1 | ARABIDOP...	490	5.0E-174		
		AT5G07200.1 | Symbols: ATGA20OX3, YAP169, GA20OX3 | gibberellin 2...	487	8.0E-173		
		AT1G60980.1 | Symbols: GA20OX4, ATGA20OX4 | gibberellin 20-oxidas...	462	8.0E-163		
		AT5G51810.2 | Symbols: AT2353, ATGA20OX2, GA20OX2 | gibberellin 2...	370	4.0E-128		
		AT4G25420.2 | Symbols: ATGA20OX1, AT2301, GA5, GA20OX1 | ARABIDOP...	363	3.0E-125		
		AT1G44090.1 | Symbols: GA20OX5, ATGA20OX5 | ARABIDOPSIS THALIANA ...	344	2.0E-116		
AUR62021271	AUR62021271-RA	AT5G51810.1 | Symbols: AT2353, ATGA20OX2, GA20OX2 | gibberellin 2...	494	2.0E-175	yes (Figure S5)	B2 - CqGA20ox2-like
		AT4G25420.1 | Symbols: ATGA20OX1, AT2301, GA5, GA20OX1 | ARABIDOP...	488	2.0E-173		
		AT5G07200.1 | Symbols: ATGA20OX3, YAP169, GA20OX3 | gibberellin 2...	483	4.0E-171		
		AT1G60980.1 | Symbols: GA20OX4, ATGA20OX4 | gibberellin 20-oxidas...	454	1.0E-159		
		AT5G51810.2 | Symbols: AT2353, ATGA20OX2, GA20OX2 | gibberellin 2...	370	4.0E-128		
		AT4G25420.2 | Symbols: ATGA20OX1, AT2301, GA5, GA20OX1 | ARABIDOP...	359	2.0E-123		
		AT1G44090.1 | Symbols: GA20OX5, ATGA20OX5 | ARABIDOPSIS THALIANA ...	340	1.0E-114		

Verification of *C. quinoa* GA20ox genes and transcript sequences

↓

Primer design for PCR cloning of *C. album* sequences (Supplementary Table 1)

↓

Sanger sequencing of *C. album* sequences and verification via BLAST analysis (Supplementary Figure 6) and phylogenetic trees (Figure 5A)

↓

Primer design (Supplementary Table 2) for *C. album* seed RT-qPCR

Figure S4 continued next page…

B BLAST analysis of GA3ox genes to identify *Chenopodium quinoa* sequences as templates for primer design

<table>
<tr><th>Arabidopsis thaliana gene identifier</th><th>Gene</th><th>Protein length (aa)</th><th>BlastN vs predicted (Phytozome website)</th><th>Score</th><th>E-value</th><th>BlastX vs Chenopodium quinoa transcripts (Phytozome website)</th><th>Score</th><th>E-value</th></tr>
<tr><td>AT1G15550</td><td>GA3ox1</td><td>358</td><td>jgi:C_Quinoa_Scaffold_3333_392 Cquinoa|392|v1.0</td><td>92.4</td><td>6.0E-16</td><td>jgi:36308453 C_quinoa_v1_0|392|AUR62011599-RA|AUR62011599-RA|LE: ...</td><td>395</td><td>1.0E-134</td></tr>
<tr><td></td><td></td><td></td><td>jgi:C_Quinoa_Scaffold_4087_392 Cquinoa|392|v1.0</td><td>50.9</td><td>2.0E-03</td><td>jgi:36305840 C_quinoa_v1_0|392|AUR62032368-RA|AUR62032368-RA|LE: ...</td><td>298</td><td>2.0E-97</td></tr>
<tr><td></td><td></td><td></td><td>jgi:C_Quinoa_Scaffold_3876_392 Cquinoa|392|v1.0</td><td>47.3</td><td>2.3E-02</td><td>jgi:36284702 C_quinoa_v1_0|392|AUR62024851-RA|AUR62024851-RA|LE: ...</td><td>290</td><td>3.0E-94</td></tr>
<tr><td></td><td></td><td></td><td></td><td></td><td></td><td>jgi:36294241 C_quinoa_v1_0|392|AUR62038693-RA|AUR62038693-RA|LE: ...</td><td>256</td><td>8.0E-81</td></tr>
<tr><td></td><td></td><td></td><td></td><td></td><td></td><td>jgi:36314863 C_quinoa_v1_0|392|AUR62015363-RA|AUR62015363-RA|SRG1...</td><td>183</td><td>6.0E-53</td></tr>
<tr><td>AT1G80340</td><td>GA3ox2</td><td>347</td><td>jgi:C_Quinoa_Scaffold_3333_392 Cquinoa|392|v1.0</td><td>68</td><td>7.0E-09</td><td>jgi:36308453 C_quinoa_v1_0|392|AUR62011599-RA|AUR62011599-RA|LE: ...</td><td>384</td><td>2.0E-130</td></tr>
<tr><td></td><td></td><td></td><td>jgi:C_Quinoa_Scaffold_3514_392 Cquinoa|392|v1.0</td><td>50.9</td><td>2.0E-03</td><td>jgi:36284702 C_quinoa_v1_0|392|AUR62024851-RA|AUR62024851-RA|LE: ...</td><td>299</td><td>7.0E-98</td></tr>
<tr><td></td><td></td><td></td><td>jgi:C_Quinoa_Scaffold_4383_392 Cquinoa|392|v1.0</td><td>46.4</td><td>2.2E-02</td><td>jgi:36305840 C_quinoa_v1_0|392|AUR62032368-RA|AUR62032368-RA|LE: ...</td><td>299</td><td>1.0E-97</td></tr>
<tr><td></td><td></td><td></td><td></td><td></td><td></td><td>jgi:36294241 C_quinoa_v1_0|392|AUR62038693-RA|AUR62038693-RA|LE: ...</td><td>253</td><td>6.0E-80</td></tr>
<tr><td>AT4G21690</td><td>GA30x3</td><td>349</td><td>jgi:C_Quinoa_Scaffold_1516_392 Cquinoa|392|v1.0</td><td>58.1</td><td>9.0E-06</td><td>jgi:36308453 C_quinoa_v1_0|392|AUR62011599-RA|AUR62011599-RA|LE: ...</td><td>320</td><td>2.0E-107</td></tr>
<tr><td></td><td></td><td></td><td></td><td></td><td></td><td>jgi:36284702 C_quinoa_v1_0|392|AUR62024851-RA|AUR62024851-RA|LE: ...</td><td>305</td><td>4.0E-102</td></tr>
<tr><td></td><td></td><td></td><td></td><td></td><td></td><td>jgi:36305840 C_quinoa_v1_0|392|AUR62032368-RA|AUR62032368-RA|LE: ...</td><td>298</td><td>2.0E-99</td></tr>
<tr><td></td><td></td><td></td><td></td><td></td><td></td><td>jgi:36294241 C_quinoa_v1_0|392|AUR62038693-RA|AUR62038693-RA|LE: ...</td><td>255</td><td>1.0E-82</td></tr>
<tr><td></td><td></td><td></td><td></td><td></td><td></td><td>jgi:36294257 C_quinoa_v1_0|392|AUR62038694-RA|AUR62038694-RA|ACO2...</td><td>194</td><td>1.0E-59</td></tr>
<tr><td>AT1G80330</td><td>GA3ox4</td><td>355</td><td>jgi:C_Quinoa_Scaffold_3333_392 Cquinoa|392|v1.0</td><td>129</td><td>2.0E-27</td><td>jgi:36308453 C_quinoa_v1_0|392|AUR62011599-RA|AUR62011599-RA|LE: ...</td><td>360</td><td>5.0E-123</td></tr>
<tr><td></td><td></td><td></td><td>jgi:C_Quinoa_Scaffold_3514_392 Cquinoa|392|v1.0</td><td>111</td><td>5.0E-22</td><td>jgi:36284702 C_quinoa_v1_0|392|AUR62024851-RA|AUR62024851-RA|LE: ...</td><td>291</td><td>1.0E-96</td></tr>
<tr><td></td><td></td><td></td><td>jgi:C_Quinoa_Scaffold_1516_392 Cquinoa|392|v1.0</td><td>66.2</td><td>2.0E-08</td><td>jgi:36305840 C_quinoa_v1_0|392|AUR62032368-RA|AUR62032368-RA|LE: ...</td><td>286</td><td>2.0E-94</td></tr>
<tr><td></td><td></td><td></td><td></td><td></td><td></td><td>jgi:36294241 C_quinoa_v1_0|392|AUR62038693-RA|AUR62038693-RA|LE: ...</td><td>256</td><td>4.0E-83</td></tr>
<tr><td></td><td></td><td></td><td></td><td></td><td></td><td>jgi:36294257 C_quinoa_v1_0|392|AUR62038694-RA|AUR62038694-RA|ACO2...</td><td>186</td><td>2.0E-56</td></tr>
</table>

plus direct search for *C. quinoa* sequences (Phytozome)

Identified potential *C. quinoa* GA3ox sequences

Verification of *C. quinoa* GA3ox genes and transcript sequences

<table>
<tr><th>Phytozome Chenopodium quinoia gene ID</th><th>Phytozome Chenopodium quinoia transcript</th><th>BLAST back to TAIR (BlastX using Chenopodium quinoa phytozome cds)</th><th>Score</th><th>E-value</th><th>DEG in Chenopodium quinoa seed transcriptome</th><th>Subgroup and name in phylogeny (Figure 6B)</th></tr>
<tr><td>AUR62011599</td><td>AUR62011599-RA</td><td>AT1G15550.1 | Symbols: GA4, ATGA3OX1, GA3OX1 | ARABIDOPSIS THALIA...</td><td>393</td><td>4.0E-136</td><td>yes (Figure S5)</td><td>C - CqGA3ox1-like</td></tr>
<tr><td></td><td></td><td>AT1G80340.1 | Symbols: GA4H, GA3OX2, ATGA3OX2 | ARABIDOPSIS THALI...</td><td>363</td><td>2.0E-124</td><td></td><td></td></tr>
<tr><td></td><td></td><td>AT1G80330.1 | Symbols: GA3OX4, ATGA3OX4 | gibberellin 3-oxidase 4...</td><td>360</td><td>6.0E-123</td><td></td><td></td></tr>
<tr><td></td><td></td><td>AT4G21690.1 | Symbols: ATGA3OX3, GA3OX3 | ARABIDOPSIS THALIANA GI...</td><td>298</td><td>3.0E-99</td><td></td><td></td></tr>
<tr><td></td><td></td><td>AT1G15550.1 | Symbols: GA4, ATGA3OX1, GA3OX1 | ARABIDOPSIS THALIA...</td><td>296</td><td>1.0E-98</td><td></td><td></td></tr>
<tr><td></td><td></td><td>AT1G80330.1 | Symbols: GA3OX4, ATGA3OX4 | gibberellin 3-oxidase 4...</td><td>286</td><td>2.0E-94</td><td></td><td></td></tr>
<tr><td></td><td></td><td>AT1G78240.1 | Symbols: OSU1, TSD2, QUA2 | TUMOROUS SHOOT DEVELOPM...</td><td>934</td><td>0.0E+00</td><td></td><td></td></tr>
<tr><td>AUR62016415</td><td>AUR62016415-RA</td><td>AT1G15550.1 | Symbols: GA4, ATGA3OX1, GA3OX1 | ARABIDOPSIS THALIA...</td><td>152</td><td>4.0E-45</td><td>yes (Figure S5)</td><td>C - CqGA3ox1-like</td></tr>
<tr><td></td><td></td><td>AT1G80340.1 | Symbols: GA4H, GA3OX2, ATGA3OX2 | ARABIDOPSIS THALI...</td><td>144</td><td>4.0E-42</td><td></td><td></td></tr>
<tr><td></td><td></td><td>AT1G80330.1 | Symbols: GA3OX4, ATGA3OX4 | gibberellin 3-oxidase 4...</td><td>127</td><td>7.0E-36</td><td></td><td></td></tr>
<tr><td></td><td></td><td>AT4G21690.1 | Symbols: ATGA3OX3, GA3OX3 | ARABIDOPSIS THALIANA GI...</td><td>127</td><td>1.0E-35</td><td></td><td></td></tr>
<tr><td></td><td></td><td>AT1G78550.1 | Symbols: no symbol available | no full name availab...</td><td>63</td><td>8.0E-12</td><td></td><td></td></tr>
<tr><td>AUR62038694</td><td>AUR62038694-RA</td><td>AT4G21690.1 | Symbols: ATGA3OX3, GA3OX3 | ARABIDOPSIS THALIANA GI...</td><td>193</td><td>2.0E-59</td><td>no</td><td>D3 - CqGA3ox4-like</td></tr>
<tr><td></td><td></td><td>AT1G80330.1 | Symbols: GA3OX4, ATGA3OX4 | gibberellin 3-oxidase 4...</td><td>186</td><td>1.0E-56</td><td></td><td></td></tr>
<tr><td></td><td></td><td>AT1G15550.1 | Symbols: GA4, ATGA3OX1, GA3OX1 | ARABIDOPSIS THALIA...</td><td>177</td><td>5.0E-53</td><td></td><td></td></tr>
<tr><td></td><td></td><td>AT1G80340.1 | Symbols: GA4H, GA3OX2, ATGA3OX2 | ARABIDOPSIS THALI...</td><td>159</td><td>3.0E-46</td><td></td><td></td></tr>
<tr><td></td><td></td><td>AT4G25420.2 | Symbols: ATGA20OX1, AT2301, GA5, GA20OX1 | ARABIDOP...</td><td>105</td><td>2.0E-26</td><td></td><td></td></tr>
<tr><td></td><td></td><td>AT4G25420.1 | Symbols: ATGA20OX1, AT2301, GA5, GA20OX1 | ARABIDOP...</td><td>106</td><td>6.0E-26</td><td></td><td></td></tr>
<tr><td></td><td></td><td>AT1G17020.1 | Symbols: ATSRG1, SRG1 | SENESCENCE-RELATED GENE 1, ...</td><td>104</td><td>1.0E-25</td><td></td><td></td></tr>
<tr><td>AUR62038695</td><td>AUR62038695-RA</td><td>AT4G21690.1 | Symbols: ATGA3OX3, GA3OX3 | ARABIDOPSIS THALIANA GI...</td><td>142</td><td>1.0E-41</td><td>no</td><td>D3 - CqGA3ox4-like</td></tr>
<tr><td></td><td></td><td>AT1G80330.1 | Symbols: GA3OX4, ATGA3OX4 | gibberellin 3-oxidase 4...</td><td>130</td><td>6.0E-37</td><td></td><td></td></tr>
<tr><td></td><td></td><td>AT1G80340.1 | Symbols: GA4H, GA3OX2, ATGA3OX2 | ARABIDOPSIS THALI...</td><td>127</td><td>1.0E-35</td><td></td><td></td></tr>
<tr><td></td><td></td><td>AT1G15550.1 | Symbols: GA4, ATGA3OX1, GA3OX1 | ARABIDOPSIS THALIA...</td><td>118</td><td>4.0E-32</td><td></td><td></td></tr>
<tr><td></td><td></td><td>AT2G38240.1 | Symbols: JOX4, JAO4 | JASMONATE-INDUCED OXYGENASE4,...</td><td>73</td><td>1.0E-15</td><td></td><td></td></tr>
<tr><td></td><td></td><td>AT4G25420.2 | Symbols: ATGA20OX1, AT2301, GA5, GA20OX1 | ARABIDOP...</td><td>69</td><td>5.0E-14</td><td></td><td></td></tr>
<tr><td></td><td></td><td>AT4G25420.1 | Symbols: ATGA20OX1, AT2301, GA5, GA20OX1 | ARABIDOP...</td><td>69</td><td>7.0E-14</td><td></td><td></td></tr>
<tr><td>AUR62038693</td><td>AUR62038693-RA</td><td>AT1G80330.1 | Symbols: GA3OX4, ATGA3OX4 | gibberellin 3-oxidase 4...</td><td>256</td><td>6.0E-83</td><td>no</td><td>D3 - CqGA3ox1-like</td></tr>
<tr><td></td><td></td><td>AT1G15550.1 | Symbols: GA4, ATGA3OX1, GA3OX1 | ARABIDOPSIS THALIA...</td><td>256</td><td>1.0E-82</td><td></td><td></td></tr>
<tr><td></td><td></td><td>AT4G21690.1 | Symbols: ATGA3OX3, GA3OX3 | ARABIDOPSIS THALIANA GI...</td><td>255</td><td>1.0E-82</td><td></td><td></td></tr>
<tr><td></td><td></td><td>AT1G80340.1 | Symbols: GA4H, GA3OX2, ATGA3OX2 | ARABIDOPSIS THALI...</td><td>236</td><td>3.0E-75</td><td></td><td></td></tr>
<tr><td></td><td></td><td>AT1G17020.1 | Symbols: ATSRG1, SRG1 | SENESCENCE-RELATED GENE 1, ...</td><td>152</td><td>7.0E-43</td><td></td><td></td></tr>
<tr><td></td><td></td><td>AT2G38240.1 | Symbols: JOX4, JAO4 | JASMONATE-INDUCED OXYGENASE4,...</td><td>152</td><td>1.0E-42</td><td></td><td></td></tr>
<tr><td></td><td></td><td>AT4G25300.1 | Symbols: no symbol available | no full name availab...</td><td>145</td><td>2.0E-40</td><td></td><td></td></tr>
<tr><td>AUR62032075</td><td>AUR62032075-RA</td><td>AT4G21690.1 | Symbols: ATGA3OX3, GA3OX3 | ARABIDOPSIS THALIANA GI...</td><td>137</td><td>2.0E-39</td><td>no</td><td>D3 - CqGA3ox1-like</td></tr>
<tr><td></td><td></td><td>AT1G80330.1 | Symbols: GA3OX4, ATGA3OX4 | gibberellin 3-oxidase 4...</td><td>125</td><td>6.0E-35</td><td></td><td></td></tr>
<tr><td></td><td></td><td>AT1G80340.1 | Symbols: GA4H, GA3OX2, ATGA3OX2 | ARABIDOPSIS THALI...</td><td>112</td><td>4.0E-30</td><td></td><td></td></tr>
<tr><td></td><td></td><td>AT1G15550.1 | Symbols: GA4, ATGA3OX1, GA3OX1 | ARABIDOPSIS THALIA...</td><td>105</td><td>3.0E-27</td><td></td><td></td></tr>
<tr><td></td><td></td><td>AT4G25420.2 | Symbols: ATGA20OX1, AT2301, GA5, GA20OX1 | ARABIDOP...</td><td>68</td><td>6.0E-14</td><td></td><td></td></tr>
<tr><td>AUR62032673</td><td>AUR62032673-RA</td><td>AT4G21690.1 | Symbols: ATGA3OX3, GA3OX3 | ARABIDOPSIS THALIANA GI...</td><td>142</td><td>2.0E-40</td><td>no</td><td>D3 - CqGA3ox1-like</td></tr>
<tr><td></td><td></td><td>AT1G80330.1 | Symbols: GA3OX4, ATGA3OX4 | gibberellin 3-oxidase 4...</td><td>134</td><td>3.0E-37</td><td></td><td></td></tr>
<tr><td></td><td></td><td>AT1G80340.1 | Symbols: GA4H, GA3OX2, ATGA3OX2 | ARABIDOPSIS THALI...</td><td>117</td><td>8.0E-31</td><td></td><td></td></tr>
<tr><td></td><td></td><td>AT1G15550.1 | Symbols: GA4, ATGA3OX1, GA3OX1 | ARABIDOPSIS THALIA...</td><td>108</td><td>1.0E-27</td><td></td><td></td></tr>
<tr><td></td><td></td><td>AT4G25420.2 | Symbols: ATGA20OX1, AT2301, GA5, GA20OX1 | ARABIDOP...</td><td>71</td><td>4.0E-14</td><td></td><td></td></tr>
<tr><td></td><td></td><td>AT4G25420.1 | Symbols: ATGA20OX1, AT2301, GA5, GA20OX1 | ARABIDOP...</td><td>71</td><td>6.0E-14</td><td></td><td></td></tr>
<tr><td></td><td></td><td>AT2G38240.1 | Symbols: JOX4, JAO4 | JASMONATE-INDUCED OXYGENASE4,...</td><td>69</td><td>2.0E-13</td><td></td><td></td></tr>
<tr><td>AUR62032368</td><td>AUR62032368-RA</td><td>AT4G21690.1 | Symbols: ATGA3OX3, GA3OX3 | ARABIDOPSIS THALIANA GI...</td><td>298</td><td>3.0E-99</td><td>no</td><td>D4 - CqGA3ox1-like</td></tr>
<tr><td></td><td></td><td>AT1G15550.1 | Symbols: GA4, ATGA3OX1, GA3OX1 | ARABIDOPSIS THALIA...</td><td>296</td><td>1.0E-98</td><td></td><td></td></tr>
<tr><td></td><td></td><td>AT1G80330.1 | Symbols: GA3OX4, ATGA3OX4 | gibberellin 3-oxidase 4...</td><td>286</td><td>2.0E-94</td><td></td><td></td></tr>
<tr><td></td><td></td><td>AT1G80340.1 | Symbols: GA4H, GA3OX2, ATGA3OX2 | ARABIDOPSIS THALI...</td><td>281</td><td>1.0E-92</td><td></td><td></td></tr>
<tr><td></td><td></td><td>AT1G78550.1 | Symbols: no symbol available | no full name availab...</td><td>176</td><td>8.0E-52</td><td></td><td></td></tr>
<tr><td>AUR62024851</td><td>AUR62024851-RA</td><td>AT4G21690.1 | Symbols: ATGA3OX3, GA3OX3 | ARABIDOPSIS THALIANA GI...</td><td>305</td><td>5.0E-102</td><td>no</td><td>D4 - CqGA3ox1-like</td></tr>
<tr><td></td><td></td><td>AT1G80330.1 | Symbols: GA3OX4, ATGA3OX4 | gibberellin 3-oxidase 4...</td><td>291</td><td>9.0E-97</td><td></td><td></td></tr>
<tr><td></td><td></td><td>AT1G15550.1 | Symbols: GA4, ATGA3OX1, GA3OX1 | ARABIDOPSIS THALIA...</td><td>291</td><td>2.0E-96</td><td></td><td></td></tr>
<tr><td></td><td></td><td>AT1G80340.1 | Symbols: GA4H, GA3OX2, ATGA3OX2 | ARABIDOPSIS THALI...</td><td>281</td><td>1.0E-92</td><td></td><td></td></tr>
<tr><td></td><td></td><td>AT1G78550.1 | Symbols: no symbol available | no full name availab...</td><td>173</td><td>1.0E-50</td><td></td><td></td></tr>
<tr><td>AUR62032076</td><td>AUR62032076-RA</td><td>AT1G15550.1 | Symbols: GA4, ATGA3OX1, GA3OX1 | ARABIDOPSIS THALIA...</td><td>137</td><td>5.0E-39</td><td>no</td><td>D4 - CqGA3ox1-like</td></tr>
<tr><td></td><td></td><td>AT1G80330.1 | Symbols: GA3OX4, ATGA3OX4 | gibberellin 3-oxidase 4...</td><td>133</td><td>7.0E-38</td><td></td><td></td></tr>
<tr><td></td><td></td><td>AT1G80340.1 | Symbols: GA4H, GA3OX2, ATGA3OX2 | ARABIDOPSIS THALI...</td><td>131</td><td>4.0E-37</td><td></td><td></td></tr>
<tr><td></td><td></td><td>AT4G21690.1 | Symbols: ATGA3OX3, GA3OX3 | ARABIDOPSIS THALIANA GI...</td><td>124</td><td>2.0E-34</td><td></td><td></td></tr>
<tr><td></td><td></td><td>AT4G25310.1 | Symbols: no symbol available | no full name availab...</td><td>114</td><td>2.0E-30</td><td></td><td></td></tr>
<tr><td></td><td></td><td>AT1G78550.2 | Symbols: no symbol available | no full name availab...</td><td>108</td><td>7.0E-29</td><td></td><td></td></tr>
<tr><td></td><td></td><td>AT4G25300.2 | Symbols: no symbol available | no full name availab...</td><td>107</td><td>1.0E-28</td><td></td><td></td></tr>
</table>

Primer design for PCR cloning of *C. album* sequences (Supplementary Table 1)

Sanger sequencing of *C. album* sequences and verification via BLAST analysis (Supplementary Figure 6) and phylogenetic trees (Figure 5B)

Primer design (Supplementary Table 2) for *C. album* seed RT-qPCR

Figure S4 continued next page…

C BLAST analysis of GA2ox genes to identify *Chenopodium quinoa* sequences as templates for primer design

<table>
<tr><th>Arabidopsis thaliana gene identifier</th><th>Gene</th><th>Protein length (aa)</th><th>BlastN vs predicted (Phytozome website)</th><th>Score</th><th>E-value</th><th>BlastX vs Chenopodium quinoa transcripts (Phytozome website)</th><th>Score</th><th>E-value</th></tr>
<tr><td>AT1G78440.1</td><td>GA2ox1</td><td>329</td><td>jgi:C_Quinoa_Scaffold_1747_392 Cquinoa|392|v1.0</td><td>125</td><td>3.0E-26</td><td>jgi:36301611 C_quinoa_v1_0|392|AUR62002752-RA|AUR62002752-RA|GA2O...</td><td>371</td><td>7.0E-127</td></tr>
<tr><td></td><td></td><td></td><td>jgi:C_Quinoa_Scaffold_1000_392 Cquinoa|392|v1.0</td><td>125</td><td>3.0E-26</td><td>jgi:36311284 C_quinoa_v1_0|392|AUR62011753-RA|AUR62011753-RA|GA2O...</td><td>280</td><td>2.0E-91</td></tr>
<tr><td></td><td></td><td></td><td>jgi:C_Quinoa_Scaffold_1611_392 Cquinoa|392|v1.0</td><td>49.1</td><td>6.0E-03</td><td>jgi:36310380 C_quinoa_v1_0|392|AUR62024597-RA|AUR62024597-RA|GA2O...</td><td>248</td><td>4.0E-78</td></tr>
<tr><td></td><td></td><td></td><td></td><td></td><td></td><td>jgi:36304895 C_quinoa_v1_0|392|AUR62030199-RA|AUR62030199-RA|GA2O...</td><td>169</td><td>6.0E-50</td></tr>
<tr><td></td><td></td><td></td><td></td><td></td><td></td><td>jgi:36308110 C_quinoa_v1_0|392|AUR62011417-RA|AUR62011417-RA|SRG1...</td><td>161</td><td>1.0E-45</td></tr>
<tr><td>AT1G30040</td><td>GA2ox2</td><td>341</td><td>jgi:C_Quinoa_Scaffold_1000_392 Cquinoa|392|v1.0</td><td>124</td><td>1.0E-25</td><td>jgi:36301611 C_quinoa_v1_0|392|AUR62002752-RA|AUR62002752-RA|GA2O...</td><td>379</td><td>1.0E-127</td></tr>
<tr><td></td><td></td><td></td><td>jgi:C_Quinoa_Scaffold_1747_392 Cquinoa|392|v1.0</td><td>117</td><td>2.0E-23</td><td>jgi:36311284 C_quinoa_v1_0|392|AUR62011753-RA|AUR62011753-RA|GA2O...</td><td>281</td><td>7.0E-90</td></tr>
<tr><td></td><td></td><td></td><td>jgi:C_Quinoa_Scaffold_3799_392 Cquinoa|392|v1.0</td><td>53.6</td><td>2.0E-04</td><td>jgi:36310380 C_quinoa_v1_0|392|AUR62024597-RA|AUR62024597-RA|GA2O...</td><td>250</td><td>3.0E-77</td></tr>
<tr><td></td><td></td><td></td><td></td><td></td><td></td><td>jgi:36304895 C_quinoa_v1_0|392|AUR62030199-RA|AUR62030199-RA|GA2O...</td><td>190</td><td>1.0E-56</td></tr>
<tr><td></td><td></td><td></td><td></td><td></td><td></td><td>jgi:36308453 C_quinoa_v1_0|392|AUR62011599-RA|AUR62011599-RA|LE: ...</td><td>161</td><td>1.0E-43</td></tr>
<tr><td>AT2G34555</td><td>GA2ox3</td><td>335</td><td>jgi:C_Quinoa_Scaffold_1000_392 Cquinoa|392|v1.0</td><td>107</td><td>7.0E-21</td><td>jgi:36301611 C_quinoa_v1_0|392|AUR62002752-RA|AUR62002752-RA|GA2O...</td><td>338</td><td>6.0E-114</td></tr>
<tr><td></td><td></td><td></td><td>jgi:C_Quinoa_Scaffold_1747_392 Cquinoa|392|v1.0</td><td>103</td><td>3.0E-19</td><td>jgi:36311284 C_quinoa_v1_0|392|AUR62011753-RA|AUR62011753-RA|GA2O...</td><td>266</td><td>3.0E-86</td></tr>
<tr><td></td><td></td><td></td><td>jgi:C_Quinoa_Scaffold_1611_392 Cquinoa|392|v1.0</td><td>52.7</td><td>5.0E-04</td><td>jgi:36310380 C_quinoa_v1_0|392|AUR62024597-RA|AUR62024597-RA|GA2O...</td><td>234</td><td>3.0E-73</td></tr>
<tr><td></td><td></td><td></td><td></td><td></td><td></td><td>jgi:36304895 C_quinoa_v1_0|392|AUR62030199-RA|AUR62030199-RA|GA2O...</td><td>167</td><td>2.0E-49</td></tr>
<tr><td></td><td></td><td></td><td></td><td></td><td></td><td>jgi:36305840 C_quinoa_v1_0|392|AUR62032368-RA|AUR62032368-RA|LE: ...</td><td>137</td><td>2.0E-36</td></tr>
<tr><td>AT1G47990</td><td>GA2ox4</td><td>321</td><td>jgi:C_Quinoa_Scaffold_1747_392 Cquinoa|392|v1.0</td><td>51.8</td><td>4.0E-04</td><td>jgi:36311284 C_quinoa_v1_0|392|AUR62011753-RA|AUR62011753-RA|GA2O...</td><td>304</td><td>4.0E-101</td></tr>
<tr><td></td><td></td><td></td><td>jgi:C_Quinoa_Scaffold_3670_392 Cquinoa|392|v1.0</td><td>46.4</td><td>1.8E-02</td><td>jgi:36310380 C_quinoa_v1_0|392|AUR62024597-RA|AUR62024597-RA|GA2O...</td><td>275</td><td>4.0E-89</td></tr>
<tr><td></td><td></td><td></td><td>jgi:C_Quinoa_Scaffold_3378_392 Cquinoa|392|v1.0</td><td>46.4</td><td>1.8E-02</td><td>jgi:36301611 C_quinoa_v1_0|392|AUR62002752-RA|AUR62002752-RA|GA2O...</td><td>268</td><td>9.0E-87</td></tr>
<tr><td></td><td></td><td></td><td></td><td></td><td></td><td>jgi:36308110 C_quinoa_v1_0|392|AUR62011417-RA|AUR62011417-RA|SRG1...</td><td>145</td><td>1.0E-39</td></tr>
</table>

plus direct search for *C. quinoa* sequences (Phytozome)

Verification of *C. quinoia* GA2ox genes and transcript sequences ← **Identified potential *C. quinoia* GA2ox sequences**

<table>
<tr><th>Phytozome Chenopodium quinoia gene ID</th><th>Phytozome Chenopodium quinoia transcript</th><th>BLAST back to TAIR (BlastX using Chenopodium quinoa phytozome cds)</th><th>Score</th><th>E-value</th><th>DEG in Chenopodium quinoa seed transcriptome</th><th>Subgroup and name in phylogeny (Suppl. Figure S7)</th></tr>
<tr><td>AUR62002752</td><td>AUR62002752-RA</td><td>AT1G30040.1 | Symbols: ATGA2OX2, GA2OX2 | gibberellin 2-oxidase, ...</td><td>384</td><td>1.0E-133</td><td>yes (Figure S5)</td><td>C19 - CqGA2ox-like</td></tr>
<tr><td></td><td></td><td>AT1G78440.1 | Symbols: GA2OX1, ATGA2OX1 | gibberellin 2-oxidase 1...</td><td>370</td><td>4.0E-128</td><td></td><td></td></tr>
<tr><td></td><td></td><td>AT2G34555.1 | Symbols: GA2OX3, ATGA2OX3 | gibberellin 2-oxidase 3...</td><td>354</td><td>1.0E-121</td><td></td><td></td></tr>
<tr><td></td><td></td><td>AT1G02400.1 | Symbols: ATGA2OX6, DTA1, ATGA2OX4, GA2OX6 | Arabido...</td><td>279</td><td>2.0E-92</td><td></td><td></td></tr>
<tr><td>AUR62030199</td><td>AUR62030199-RA</td><td>AT1G30040.2 | Symbols: ATGA2OX2, GA2OX2 | gibberellin 2-oxidase, ...</td><td>191</td><td>7.0E-61</td><td>no</td><td>C19 - CqGA2ox-like</td></tr>
<tr><td></td><td></td><td>AT1G30040.1 | Symbols: ATGA2OX2, GA2OX2 | gibberellin 2-oxidase, ...</td><td>191</td><td>5.0E-60</td><td></td><td></td></tr>
<tr><td></td><td></td><td>AT2G34555.1 | Symbols: GA2OX3, ATGA2OX3 | gibberellin 2-oxidase 3...</td><td>183</td><td>8.0E-57</td><td></td><td></td></tr>
<tr><td></td><td></td><td>AT1G78440.1 | Symbols: GA2OX1, ATGA2OX1 | gibberellin 2-oxidase 1...</td><td>168</td><td>3.0E-51</td><td></td><td></td></tr>
<tr><td></td><td></td><td>AT1G02400.2 | Symbols: ATGA2OX6, DTA1, ATGA2OX4, GA2OX6 | Arabido...</td><td>138</td><td>4.0E-40</td><td></td><td></td></tr>
<tr><td></td><td></td><td>AT1G02400.1 | Symbols: ATGA2OX6, DTA1, ATGA2OX4, GA2OX6 | Arabido...</td><td>137</td><td>2.0E-39</td><td></td><td></td></tr>
<tr><td></td><td></td><td>AT1G47990.1 | Symbols: ATGA2OX4, GA2OX4 | Arabidopsis thaliana gi...</td><td>113</td><td>4.0E-30</td><td></td><td></td></tr>
<tr><td>AUR62011753</td><td>AUR62011753-RA</td><td>AT1G02400.1 | Symbols: ATGA2OX6, DTA1, ATGA2OX4, GA2OX6 | Arabido...</td><td>337</td><td>2.0E-115</td><td>yes (Figure S5)</td><td>C19 - CqGA2ox2-like</td></tr>
<tr><td></td><td></td><td>AT1G47990.1 | Symbols: ATGA2OX4, GA2OX4 | Arabidopsis thaliana gi...</td><td>303</td><td>3.0E-102</td><td></td><td></td></tr>
<tr><td></td><td></td><td>AT1G30040.1 | Symbols: ATGA2OX2, GA2OX2 | gibberellin 2-oxidase, ...</td><td>284</td><td>2.0E-94</td><td></td><td></td></tr>
<tr><td></td><td></td><td>AT2G34555.1 | Symbols: GA2OX3, ATGA2OX3 | gibberellin 2-oxidase 3...</td><td>281</td><td>2.0E-93</td><td></td><td></td></tr>
<tr><td></td><td></td><td>AT1G78440.1 | Symbols: GA2OX1, ATGA2OX1 | gibberellin 2-oxidase 1...</td><td>280</td><td>8.0E-93</td><td></td><td></td></tr>
<tr><td>AUR62024597</td><td>AUR62024597-RA</td><td>AT1G02400.1 | Symbols: ATGA2OX6, DTA1, ATGA2OX4, GA2OX6 | Arabido...</td><td>313</td><td>5.0E-105</td><td>no</td><td>C19 - CqGA2ox2-like</td></tr>
<tr><td></td><td></td><td>AT1G47990.1 | Symbols: ATGA2OX4, GA2OX4 | Arabidopsis thaliana gi...</td><td>275</td><td>3.0E-90</td><td></td><td></td></tr>
<tr><td></td><td></td><td>AT1G30040.1 | Symbols: ATGA2OX2, GA2OX2 | gibberellin 2-oxidase, ...</td><td>253</td><td>2.0E-81</td><td></td><td></td></tr>
<tr><td></td><td></td><td>AT2G34555.1 | Symbols: GA2OX3, ATGA2OX3 | gibberellin 2-oxidase 3...</td><td>249</td><td>2.0E-80</td><td></td><td></td></tr>
<tr><td></td><td></td><td>AT1G78440.1 | Symbols: GA2OX1, ATGA2OX1 | gibberellin 2-oxidase 1...</td><td>246</td><td>3.0E-79</td><td></td><td></td></tr>
<tr><td></td><td></td><td>AT1G02400.2 | Symbols: ATGA2OX6, DTA1, ATGA2OX4, GA2OX6 | Arabido...</td><td>232</td><td>2.0E-74</td><td></td><td></td></tr>
<tr><td></td><td></td><td>AT1G30040.2 | Symbols: ATGA2OX2, GA2OX2 | gibberellin 2-oxidase, ...</td><td>189</td><td>8.0E-58</td><td></td><td></td></tr>
<tr><td>AUR62022014</td><td>AUR62022014-RA</td><td>AT4G21200.1 | Symbols: ATGA2OX8, GA2OX8 | gibberellin 2-oxidase 8...</td><td>236</td><td>1.0E-77</td><td>yes (Figure S5)</td><td>C20 - CqGA2ox8-like</td></tr>
<tr><td></td><td></td><td>AT1G50960.1 | Symbols: GA2OX7, ATGA2OX7 | ARABIDOPSIS THALIANA GI...</td><td>147</td><td>3.0E-43</td><td></td><td></td></tr>
<tr><td></td><td></td><td>AT4G10490.1 | Symbols: DLO2 | DMR6-LIKE OXYGENASE 2 | chr4:648390...</td><td>105</td><td>5.0E-27</td><td></td><td></td></tr>
<tr><td></td><td></td><td>AT5G58660.1 | Symbols: GA2ox9 | Gibberellin 2-oxidase 9 | chr5:23...</td><td>99</td><td>1.0E-24</td><td></td><td></td></tr>
<tr><td></td><td></td><td>AT5G07200.1 | Symbols: ATGA20OX3, YAP169, GA20OX3 | gibberellin 2...</td><td>99</td><td>2.0E-24</td><td></td><td></td></tr>
<tr><td></td><td></td><td>AT3G47190.1 | Symbols: GA2ox10 | Gibberellin 2-oxidase 10 | chr3:...</td><td>97</td><td>6.0E-24</td><td></td><td></td></tr>
<tr><td></td><td></td><td>AT5G51810.1 | Symbols: AT2353, ATGA20OX2, GA20OX2 | gibberellin 2...</td><td>94</td><td>2.0E-22</td><td></td><td></td></tr>
<tr><td>AUR62022015</td><td>AUR62022015-RA</td><td>AT4G21200.1 | Symbols: ATGA2OX8, GA2OX8 | gibberellin 2-oxidase 8...</td><td>402</td><td>3.0E-140</td><td>no</td><td>C20 - CqGA2ox8-like</td></tr>
<tr><td></td><td></td><td>AT1G50960.1 | Symbols: GA2OX7, ATGA2OX7 | ARABIDOPSIS THALIANA GI...</td><td>266</td><td>4.0E-87</td><td></td><td></td></tr>
<tr><td></td><td></td><td>AT5G58660.1 | Symbols: GA2ox9 | Gibberellin 2-oxidase 9 | chr5:23...</td><td>159</td><td>1.0E-45</td><td></td><td></td></tr>
<tr><td></td><td></td><td>AT4G21200.3 | Symbols: ATGA2OX8, GA2OX8 | gibberellin 2-oxidase 8...</td><td>150</td><td>4.0E-44</td><td></td><td></td></tr>
<tr><td></td><td></td><td>AT4G25420.1 | Symbols: ATGA20OX1, AT2301, GA5, GA20OX1 | ARABIDOP...</td><td>155</td><td>1.0E-43</td><td></td><td></td></tr>
<tr><td></td><td></td><td>AT5G05600.1 | Symbols: JAO2, JOX2 | JASMONATE-INDUCED OXYGENASE2,...</td><td>155</td><td>1.0E-43</td><td></td><td></td></tr>
<tr><td></td><td></td><td>AT3G19010.4 | Symbols: no symbol available | no full name availab...</td><td>154</td><td>2.0E-43</td><td></td><td></td></tr>
<tr><td>AUR62001627</td><td>AUR62001627-RA</td><td>AT5G58660.2 | Symbols: GA2ox9 | Gibberellin 2-oxidase 9 | chr5:23...</td><td>205</td><td>3.0E-65</td><td>no</td><td>C20 - CqGA2ox8-like</td></tr>
<tr><td></td><td></td><td>AT5G58660.1 | Symbols: GA2ox9 | Gibberellin 2-oxidase 9 | chr5:23...</td><td>205</td><td>3.0E-64</td><td></td><td></td></tr>
<tr><td></td><td></td><td>AT3G47190.1 | Symbols: GA2ox10 | Gibberellin 2-oxidase 10 | chr3:...</td><td>197</td><td>3.0E-61</td><td></td><td></td></tr>
<tr><td></td><td></td><td>AT4G21200.1 | Symbols: ATGA2OX8, GA2OX8 | gibberellin 2-oxidase 8...</td><td>113</td><td>3.0E-29</td><td></td><td></td></tr>
<tr><td></td><td></td><td>AT4G25300.1 | Symbols: no symbol available | no full name availab...</td><td>107</td><td>8.0E-27</td><td></td><td></td></tr>
<tr><td></td><td></td><td>AT1G50960.1 | Symbols: GA2OX7, ATGA2OX7 | ARABIDOPSIS THALIANA GI...</td><td>103</td><td>1.0E-25</td><td></td><td></td></tr>
<tr><td></td><td></td><td>AT1G17020.1 | Symbols: ATSRG1, SRG1 | SENESCENCE-RELATED GENE 1, ...</td><td>103</td><td>2.0E-25</td><td></td><td></td></tr>
<tr><td>AUR62020152</td><td>AUR62020152-RA</td><td>AT5G58660.2 | Symbols: GA2ox9 | Gibberellin 2-oxidase 9 | chr5:23...</td><td>204</td><td>2.0E-64</td><td>no</td><td>C20 - CqGA2ox8-like</td></tr>
<tr><td></td><td></td><td>AT5G58660.1 | Symbols: GA2ox9 | Gibberellin 2-oxidase 9 | chr5:23...</td><td>202</td><td>7.0E-63</td><td></td><td></td></tr>
<tr><td></td><td></td><td>AT3G47190.1 | Symbols: GA2ox10 | Gibberellin 2-oxidase 10 | chr3:...</td><td>185</td><td>2.0E-56</td><td></td><td></td></tr>
<tr><td></td><td></td><td>AT4G21200.1 | Symbols: ATGA2OX8, GA2OX8 | gibberellin 2-oxidase 8...</td><td>115</td><td>7.0E-30</td><td></td><td></td></tr>
<tr><td></td><td></td><td>AT1G50960.1 | Symbols: GA2OX7, ATGA2OX7 | ARABIDOPSIS THALIANA GI...</td><td>103</td><td>3.0E-25</td><td></td><td></td></tr>
<tr><td></td><td></td><td>AT1G17020.1 | Symbols: ATSRG1, SRG1 | SENESCENCE-RELATED GENE 1, ...</td><td>93</td><td>1.0E-21</td><td></td><td></td></tr>
<tr><td></td><td></td><td>AT4G25300.1 | Symbols: no symbol available | no full name availab...</td><td>93</td><td>2.0E-21</td><td></td><td></td></tr>
</table>

Primer design for PCR cloning of *C. album* sequences (Supplementary Table 1)

↓

Sanger sequencing of *C. album* sequences and verification via BLAST analysis (Supplementary Figure 6) and phylogenetic trees (Suppl. Figure 7)

↓

Primer design (Supplementary Table 2) for *C. album* seed RT-qPCR

Figure S4 continued next page…

D BLAST analysis of NCED genes to identify *Chenopodium quinoa* sequences as templates for primer design

<table>
<tr><th>Arabidopsis thaliana gene identifier</th><th>Gene</th><th>Protein length (aa)</th><th>BlastN vs predicted (Phytozome website)</th><th>Score</th><th>E-value</th><th>BlastX vs Chenopodium quinoa transcripts (Phytozome website)</th><th>Score</th><th>E-value</th></tr>
<tr><td>AT3G63520</td><td>NCED2</td><td>583</td><td>jgi:C_Quinoa_Scaffold_1747_392 Cquinoa|392|v1.0</td><td>547</td><td>4.0E-153</td><td>jgi:36303079 C_quinoa_v1_0|392|AUR62037951-RA|AUR62037951-RA|NCED...</td><td>682</td><td>0.0E+00</td></tr>
<tr><td></td><td></td><td></td><td>jgi:C_Quinoa_Scaffold_1000_392 Cquinoa|392|v1.0</td><td>497</td><td>7.0E-138</td><td>jgi:36306192 C_quinoa_v1_0|392|AUR62043087-RA|AUR62043087-RA|NCED...</td><td>679</td><td>0.0E+00</td></tr>
<tr><td></td><td></td><td></td><td>jgi:C_Quinoa_Scaffold_2370_392 Cquinoa|392|v1.0</td><td>489</td><td>1.0E-135</td><td>jgi:36301918 C_quinoa_v1_0|392|AUR62002735-RA|AUR62002735-RA|NCED...</td><td>655</td><td>0.0E+00</td></tr>
<tr><td></td><td></td><td></td><td></td><td></td><td></td><td>jgi:36304873 C_quinoa_v1_0|392|AUR62030185-RA|AUR62030185-RA|NCED...</td><td>624</td><td>0.0E+00</td></tr>
<tr><td></td><td></td><td></td><td></td><td></td><td></td><td>jgi:36292536 C_quinoa_v1_0|392|AUR62003579-RA|AUR62003579-RA|NCED...</td><td>572</td><td>0.0E+00</td></tr>
<tr><td></td><td></td><td></td><td></td><td></td><td></td><td>jgi:36318712 C_quinoa_v1_0|392|AUR62015113-RA|AUR62015113-RA|NCED...</td><td>479</td><td>3.0E-162</td></tr>
<tr><td></td><td></td><td></td><td></td><td></td><td></td><td>jgi:36291573 C_quinoa_v1_0|392|AUR62017576-RA|AUR62017576-RA|NCED...</td><td>414</td><td>2.0E-138</td></tr>
<tr><td></td><td></td><td></td><td></td><td></td><td></td><td>jgi:36310268 C_quinoa_v1_0|392|AUR62018623-RA|AUR62018623-RA|CCD4...</td><td>314</td><td>7.0E-97</td></tr>
<tr><td>AT3G14440</td><td>NCED3</td><td>599</td><td>jgi:C_Quinoa_Scaffold_1770_392 Cquinoa|392|v1.0</td><td>713</td><td>0.0E+00</td><td>jgi:36306192 C_quinoa_v1_0|392|AUR62043087-RA|AUR62043087-RA|NCED...</td><td>781</td><td>0.0E+00</td></tr>
<tr><td></td><td></td><td></td><td>jgi:C_Quinoa_Scaffold_2674_392 Cquinoa|392|v1.0</td><td>710</td><td>0.0E+00</td><td>jgi:36303079 C_quinoa_v1_0|392|AUR62037951-RA|AUR62037951-RA|NCED...</td><td>774</td><td>0.0E+00</td></tr>
<tr><td></td><td></td><td></td><td>jgi:C_Quinoa_Scaffold_1747_392 Cquinoa|392|v1.0</td><td>564</td><td>6.0E-158</td><td>jgi:36301918 C_quinoa_v1_0|392|AUR62002735-RA|AUR62002735-RA|NCED...</td><td>711</td><td>0.0E+00</td></tr>
<tr><td></td><td></td><td></td><td></td><td></td><td></td><td>jgi:36304873 C_quinoa_v1_0|392|AUR62030185-RA|AUR62030185-RA|NCED...</td><td>684</td><td>0.0E+00</td></tr>
<tr><td></td><td></td><td></td><td></td><td></td><td></td><td>jgi:36292536 C_quinoa_v1_0|392|AUR62003579-RA|AUR62003579-RA|NCED...</td><td>616</td><td>0.0E+00</td></tr>
<tr><td></td><td></td><td></td><td></td><td></td><td></td><td>jgi:36318712 C_quinoa_v1_0|392|AUR62015113-RA|AUR62015113-RA|NCED...</td><td>571</td><td>0.0E+00</td></tr>
<tr><td></td><td></td><td></td><td></td><td></td><td></td><td>jgi:36291573 C_quinoa_v1_0|392|AUR62017576-RA|AUR62017576-RA|NCED...</td><td>462</td><td>9.0E-156</td></tr>
<tr><td></td><td></td><td></td><td></td><td></td><td></td><td>jgi:36310268 C_quinoa_v1_0|392|AUR62018623-RA|AUR62018623-RA|CCD4...</td><td>343</td><td>1.0E-106</td></tr>
<tr><td>AT1G30100</td><td>NCED5</td><td>589</td><td>jgi:C_Quinoa_Scaffold_1747_392 Cquinoa|392|v1.0</td><td>579</td><td>2.0E-162</td><td>jgi:36303079 C_quinoa_v1_0|392|AUR62037951-RA|AUR62037951-RA|NCED...</td><td>803</td><td>0.0E+00</td></tr>
<tr><td></td><td></td><td></td><td>jgi:C_Quinoa_Scaffold_1000_392 Cquinoa|392|v1.0</td><td>521</td><td>5.0E-145</td><td>jgi:36306192 C_quinoa_v1_0|392|AUR62043087-RA|AUR62043087-RA|NCED...</td><td>801</td><td>0.0E+00</td></tr>
<tr><td></td><td></td><td></td><td>jgi:C_Quinoa_Scaffold_2674_392 Cquinoa|392|v1.0</td><td>519</td><td>2.0E-144</td><td>jgi:36301918 C_quinoa_v1_0|392|AUR62002735-RA|AUR62002735-RA|NCED...</td><td>773</td><td>0.0E+00</td></tr>
<tr><td></td><td></td><td></td><td></td><td></td><td></td><td>jgi:36304873 C_quinoa_v1_0|392|AUR62030185-RA|AUR62030185-RA|NCED...</td><td>725</td><td>0.0E+00</td></tr>
<tr><td></td><td></td><td></td><td></td><td></td><td></td><td>jgi:36292536 C_quinoa_v1_0|392|AUR62003579-RA|AUR62003579-RA|NCED...</td><td>670</td><td>0.0E+00</td></tr>
<tr><td></td><td></td><td></td><td></td><td></td><td></td><td>jgi:36318712 C_quinoa_v1_0|392|AUR62015113-RA|AUR62015113-RA|NCED...</td><td>557</td><td>0.0E+00</td></tr>
<tr><td></td><td></td><td></td><td></td><td></td><td></td><td>jgi:36291573 C_quinoa_v1_0|392|AUR62017576-RA|AUR62017576-RA|NCED...</td><td>437</td><td>1.0E-148</td></tr>
<tr><td></td><td></td><td></td><td></td><td></td><td></td><td>jgi:36310268 C_quinoa_v1_0|392|AUR62018623-RA|AUR62018623-RA|CCD4...</td><td>373</td><td>1.0E-120</td></tr>
<tr><td>AT3G24220</td><td>NCED6</td><td>577</td><td>jgi:C_Quinoa_Scaffold_2888_392 Cquinoa|392|v1.0</td><td>400</td><td>1.0E-108</td><td>jgi:36303079 C_quinoa_v1_0|392|AUR62037951-RA|AUR62037951-RA|NCED...</td><td>639</td><td>0.0E+00</td></tr>
<tr><td></td><td></td><td></td><td>jgi:C_Quinoa_Scaffold_3458_392 Cquinoa|392|v1.0</td><td>394</td><td>5.0E-107</td><td>jgi:36318712 C_quinoa_v1_0|392|AUR62015113-RA|AUR62015113-RA|NCED...</td><td>634</td><td>0.0E+00</td></tr>
<tr><td></td><td></td><td></td><td>jgi:C_Quinoa_Scaffold_1770_392 Cquinoa|392|v1.0</td><td>185</td><td>4.0E-44</td><td>jgi:36306192 C_quinoa_v1_0|392|AUR62043087-RA|AUR62043087-RA|NCED...</td><td>632</td><td>0.0E+00</td></tr>
<tr><td></td><td></td><td></td><td></td><td></td><td></td><td>jgi:36301918 C_quinoa_v1_0|392|AUR62002735-RA|AUR62002735-RA|NCED...</td><td>604</td><td>0.0E+00</td></tr>
<tr><td></td><td></td><td></td><td></td><td></td><td></td><td>jgi:36304873 C_quinoa_v1_0|392|AUR62030185-RA|AUR62030185-RA|NCED...</td><td>575</td><td>0.0E+00</td></tr>
<tr><td></td><td></td><td></td><td></td><td></td><td></td><td>jgi:36292536 C_quinoa_v1_0|392|AUR62003579-RA|AUR62003579-RA|NCED...</td><td>521</td><td>0.0E+00</td></tr>
<tr><td></td><td></td><td></td><td></td><td></td><td></td><td>jgi:36291573 C_quinoa_v1_0|392|AUR62017576-RA|AUR62017576-RA|NCED...</td><td>501</td><td>9.0E-175</td></tr>
<tr><td></td><td></td><td></td><td></td><td></td><td></td><td>jgi:36310268 C_quinoa_v1_0|392|AUR62018623-RA|AUR62018623-RA|CCD4...</td><td>336</td><td>5.0E-107</td></tr>
<tr><td>AT1G78390</td><td>NCED9</td><td>657</td><td>jgi:C_Quinoa_Scaffold_2674_392 Cquinoa|392|v1.0</td><td>505</td><td>4.0E-140</td><td>jgi:36306192 C_quinoa_v1_0|392|AUR62043087-RA|AUR62043087-RA|NCED...</td><td>754</td><td>0.0E+00</td></tr>
<tr><td></td><td></td><td></td><td>jgi:C_Quinoa_Scaffold_1770_392 Cquinoa|392|v1.0</td><td>477</td><td>6.0E-132</td><td>jgi:36303079 C_quinoa_v1_0|392|AUR62037951-RA|AUR62037951-RA|NCED...</td><td>753</td><td>0.0E+00</td></tr>
<tr><td></td><td></td><td></td><td>jgi:C_Quinoa_Scaffold_1747_392 Cquinoa|392|v1.0</td><td>453</td><td>2.0E-124</td><td>jgi:36301918 C_quinoa_v1_0|392|AUR62002735-RA|AUR62002735-RA|NCED...</td><td>724</td><td>0.0E+00</td></tr>
<tr><td></td><td></td><td></td><td></td><td></td><td></td><td>jgi:36304873 C_quinoa_v1_0|392|AUR62030185-RA|AUR62030185-RA|NCED...</td><td>681</td><td>0.0E+00</td></tr>
<tr><td></td><td></td><td></td><td></td><td></td><td></td><td>jgi:36292536 C_quinoa_v1_0|392|AUR62003579-RA|AUR62003579-RA|NCED...</td><td>613</td><td>0.0E+00</td></tr>
<tr><td></td><td></td><td></td><td></td><td></td><td></td><td>jgi:36318712 C_quinoa_v1_0|392|AUR62015113-RA|AUR62015113-RA|NCED...</td><td>564</td><td>0.0E+00</td></tr>
<tr><td></td><td></td><td></td><td></td><td></td><td></td><td>jgi:36291573 C_quinoa_v1_0|392|AUR62017576-RA|AUR62017576-RA|NCED...</td><td>459</td><td>6.0E-157</td></tr>
<tr><td></td><td></td><td></td><td></td><td></td><td></td><td>jgi:36310268 C_quinoa_v1_0|392|AUR62018623-RA|AUR62018623-RA|CCD4...</td><td>334</td><td>4.0E-105</td></tr>
</table>

plus direct search for *C. quinoa* sequences (Phytozome)

Identified potential *C. quinoa* NCED sequences

Verification of *C. quinoa* NCED genes and transcript sequences

<table>
<tr><th>Phytozome Chenopodium quinoia gene ID</th><th>Phytozome Chenopodium quinoia transcript</th><th>BLAST back to TAIR (BlastX using Chenopodium quinoa phytozome cds)</th><th>Score</th><th>E-value</th><th>DEG in Chenopodium quinoa seed transcriptome</th><th>Subgroup and name in phylogeny (Figure 7A)</th></tr>
<tr><td>AUR62002735</td><td>AUR62002735-RA</td><td>AT1G30100.1 | Symbols: NCED5, ATNCED5 | nine-cis-epoxycarotenoid ...</td><td>772</td><td>0.0E+00</td><td>no</td><td>2/5 - CqNCED1</td></tr>
<tr><td></td><td></td><td>AT1G78390.1 | Symbols: ATNCED9, NCED9 | NINE-CIS-EPOXYCAROTENOID ...</td><td>747</td><td>0.0E+00</td><td></td><td></td></tr>
<tr><td></td><td></td><td>AT4G18350.1 | Symbols: ATNCED2, NCED2 | NINE-CIS-EPOXYCAROTENOID ...</td><td>740</td><td>0.0E+00</td><td></td><td></td></tr>
<tr><td></td><td></td><td>AT3G14440.1 | Symbols: SIS7, ATNCED3, NCED3, STO1 | SALT TOLERANT...</td><td>729</td><td>0.0E+00</td><td></td><td></td></tr>
<tr><td></td><td></td><td>AT3G24220.1 | Symbols: NCED6, ATNCED6 | nine-cis-epoxycarotenoid ...</td><td>604</td><td>0.0E+00</td><td></td><td></td></tr>
<tr><td></td><td></td><td>AT4G19170.1 | Symbols: NCED4, CCD4 | carotenoid cleavage dioxygen...</td><td>329</td><td>2.0E-105</td><td></td><td></td></tr>
<tr><td>AUR62030185</td><td>AUR62030185-RA</td><td>AT1G30100.1 | Symbols: NCED5, ATNCED5 | nine-cis-epoxycarotenoid ...</td><td>744</td><td>0.0E+00</td><td>no</td><td>2/5 - CqNCED1</td></tr>
<tr><td></td><td></td><td>AT1G78390.1 | Symbols: ATNCED9, NCED9 | NINE-CIS-EPOXYCAROTENOID ...</td><td>726</td><td>0.0E+00</td><td></td><td></td></tr>
<tr><td></td><td></td><td>AT3G14440.1 | Symbols: SIS7, ATNCED3, NCED3, STO1 | SALT TOLERANT...</td><td>725</td><td>0.0E+00</td><td></td><td></td></tr>
<tr><td></td><td></td><td>AT4G18350.1 | Symbols: ATNCED2, NCED2 | NINE-CIS-EPOXYCAROTENOID ...</td><td>723</td><td>0.0E+00</td><td></td><td></td></tr>
<tr><td></td><td></td><td>AT3G24220.1 | Symbols: NCED6, ATNCED6 | nine-cis-epoxycarotenoid ...</td><td>586</td><td>0.0E+00</td><td></td><td></td></tr>
<tr><td>AUR62003579</td><td>AUR62003579-RA</td><td>AT1G30100.1 | Symbols: NCED5, ATNCED5 | nine-cis-epoxycarotenoid ...</td><td>670</td><td>0.0E+00</td><td>no</td><td>2/5 - CqNCED2</td></tr>
<tr><td></td><td></td><td>AT4G18350.1 | Symbols: ATNCED2, NCED2 | NINE-CIS-EPOXYCAROTENOID ...</td><td>645</td><td>0.0E+00</td><td></td><td></td></tr>
<tr><td></td><td></td><td>AT1G78390.1 | Symbols: ATNCED9, NCED9 | NINE-CIS-EPOXYCAROTENOID ...</td><td>635</td><td>0.0E+00</td><td></td><td></td></tr>
<tr><td></td><td></td><td>AT3G14440.1 | Symbols: SIS7, ATNCED3, NCED3, STO1 | SALT TOLERANT...</td><td>635</td><td>0.0E+00</td><td></td><td></td></tr>
<tr><td></td><td></td><td>AT3G24220.1 | Symbols: NCED6, ATNCED6 | nine-cis-epoxycarotenoid ...</td><td>520</td><td>0.0E+00</td><td></td><td></td></tr>
<tr><td></td><td></td><td>AT4G19170.1 | Symbols: NCED4, CCD4 | carotenoid cleavage dioxygen...</td><td>302</td><td>5.0E-96</td><td></td><td></td></tr>
<tr><td>AUR62037951</td><td>AUR62037951-RA</td><td>AT1G30100.1 | Symbols: NCED5, ATNCED5 | nine-cis-epoxycarotenoid ...</td><td>802</td><td>0.0E+00</td><td>no</td><td>3/9 - CqNCED1</td></tr>
<tr><td></td><td></td><td>AT3G14440.1 | Symbols: SIS7, ATNCED3, NCED3, STO1 | SALT TOLERANT...</td><td>795</td><td>0.0E+00</td><td></td><td></td></tr>
<tr><td></td><td></td><td>AT1G78390.1 | Symbols: ATNCED9, NCED9 | NINE-CIS-EPOXYCAROTENOID ...</td><td>778</td><td>0.0E+00</td><td></td><td></td></tr>
<tr><td></td><td></td><td>AT4G18350.1 | Symbols: ATNCED2, NCED2 | NINE-CIS-EPOXYCAROTENOID ...</td><td>758</td><td>0.0E+00</td><td></td><td></td></tr>
<tr><td></td><td></td><td>AT3G24220.1 | Symbols: NCED6, ATNCED6 | nine-cis-epoxycarotenoid ...</td><td>633</td><td>0.0E+00</td><td></td><td></td></tr>
<tr><td></td><td></td><td>AT4G19170.1 | Symbols: NCED4, CCD4 | carotenoid cleavage dioxygen...</td><td>381</td><td>8.0E-125</td><td></td><td></td></tr>
<tr><td>AUR62043087</td><td>AUR62043087-RA</td><td>AT3G14440.1 | Symbols: SIS7, ATNCED3, NCED3, STO1 | SALT TOLERANT...</td><td>801</td><td>0.0E+00</td><td>no</td><td>3/9 - CqNCED1</td></tr>
<tr><td></td><td></td><td>AT1G30100.1 | Symbols: NCED5, ATNCED5 | nine-cis-epoxycarotenoid ...</td><td>798</td><td>0.0E+00</td><td></td><td></td></tr>
<tr><td></td><td></td><td>AT1G78390.1 | Symbols: ATNCED9, NCED9 | NINE-CIS-EPOXYCAROTENOID ...</td><td>779</td><td>0.0E+00</td><td></td><td></td></tr>
<tr><td></td><td></td><td>AT4G18350.1 | Symbols: ATNCED2, NCED2 | NINE-CIS-EPOXYCAROTENOID ...</td><td>753</td><td>0.0E+00</td><td></td><td></td></tr>
<tr><td></td><td></td><td>AT3G24220.1 | Symbols: NCED6, ATNCED6 | nine-cis-epoxycarotenoid ...</td><td>629</td><td>0.0E+00</td><td></td><td></td></tr>
<tr><td></td><td></td><td>AT4G19170.1 | Symbols: NCED4, CCD4 | carotenoid cleavage dioxygen...</td><td>375</td><td>9.0E-123</td><td></td><td></td></tr>
<tr><td></td><td></td><td>AT3G63520.1 | Symbols: ATNCED1, ATCCD1, CCD1, NCED1 | carotenoid ...</td><td>346</td><td>3.0E-112</td><td></td><td></td></tr>
<tr><td>AUR62015113</td><td>AUR62015113-RA</td><td>AT3G24220.1 | Symbols: NCED6, ATNCED6 | nine-cis-epoxycarotenoid ...</td><td>634</td><td>0.0E+00</td><td>yes (Figure S5)</td><td>6 - CqNCED6</td></tr>
<tr><td></td><td></td><td>AT1G78390.1 | Symbols: ATNCED9, NCED9 | NINE-CIS-EPOXYCAROTENOID ...</td><td>588</td><td>0.0E+00</td><td></td><td></td></tr>
<tr><td></td><td></td><td>AT3G14440.1 | Symbols: SIS7, ATNCED3, NCED3, STO1 | SALT TOLERANT...</td><td>584</td><td>0.0E+00</td><td></td><td></td></tr>
<tr><td></td><td></td><td>AT1G30100.1 | Symbols: NCED5, ATNCED5 | nine-cis-epoxycarotenoid ...</td><td>557</td><td>0.0E+00</td><td></td><td></td></tr>
<tr><td></td><td></td><td>AT4G18350.1 | Symbols: ATNCED2, NCED2 | NINE-CIS-EPOXYCAROTENOID ...</td><td>553</td><td>0.0E+00</td><td></td><td></td></tr>
<tr><td></td><td></td><td>AT4G19170.1 | Symbols: NCED4, CCD4 | carotenoid cleavage dioxygen...</td><td>302</td><td>6.0E-96</td><td></td><td></td></tr>
<tr><td>AUR62017576</td><td>AUR62017576-RA</td><td>AT3G24220.1 | Symbols: NCED6, ATNCED6 | nine-cis-epoxycarotenoid ...</td><td>501</td><td>2.0E-175</td><td>yes (Figure S5)</td><td>6 - CqNCED6</td></tr>
<tr><td></td><td></td><td>AT3G14440.1 | Symbols: SIS7, ATNCED3, NCED3, STO1 | SALT TOLERANT...</td><td>461</td><td>1.0E-159</td><td></td><td></td></tr>
<tr><td></td><td></td><td>AT1G78390.1 | Symbols: ATNCED9, NCED9 | NINE-CIS-EPOXYCAROTENOID ...</td><td>459</td><td>3.0E-158</td><td></td><td></td></tr>
<tr><td></td><td></td><td>AT4G18350.1 | Symbols: ATNCED2, NCED2 | NINE-CIS-EPOXYCAROTENOID ...</td><td>438</td><td>6.0E-151</td><td></td><td></td></tr>
<tr><td></td><td></td><td>AT1G30100.1 | Symbols: NCED5, ATNCED5 | nine-cis-epoxycarotenoid ...</td><td>436</td><td>5.0E-150</td><td></td><td></td></tr>
<tr><td></td><td></td><td>AT4G19170.1 | Symbols: NCED4, CCD4 | carotenoid cleavage dioxygen...</td><td>253</td><td>1.0E-78</td><td></td><td></td></tr>
<tr><td>AUR62017575</td><td>AUR62017575-RA</td><td>AT3G24220.1 | Symbols: NCED6, ATNCED6 | nine-cis-epoxycarotenoid ...</td><td>121</td><td>9.0E-33</td><td>no</td><td>6*</td></tr>
<tr><td></td><td></td><td>AT4G18350.1 | Symbols: ATNCED2, NCED2 | NINE-CIS-EPOXYCAROTENOID ...</td><td>113</td><td>7.0E-30</td><td></td><td>*short sequence, not</td></tr>
<tr><td></td><td></td><td>AT1G78390.1 | Symbols: ATNCED9, NCED9 | NINE-CIS-EPOXYCAROTENOID ...</td><td>110</td><td>1.0E-28</td><td></td><td>included in phylogeny</td></tr>
<tr><td></td><td></td><td>AT1G30100.1 | Symbols: NCED5, ATNCED5 | nine-cis-epoxycarotenoid ...</td><td>103</td><td>2.0E-26</td><td></td><td></td></tr>
<tr><td></td><td></td><td>AT3G14440.1 | Symbols: SIS7, ATNCED3, NCED3, STO1 | SALT TOLERANT...</td><td>96</td><td>1.0E-23</td><td></td><td></td></tr>
<tr><td></td><td></td><td>AT4G19170.1 | Symbols: NCED4, CCD4 | carotenoid cleavage dioxygen...</td><td>79</td><td>7.0E-18</td><td></td><td></td></tr>
<tr><td></td><td></td><td>AT3G63520.1 | Symbols: ATNCED1, ATCCD1, CCD1, NCED1 | carotenoid ...</td><td>60</td><td>4.0E-11</td><td></td><td></td></tr>
<tr><td>AUR62025811</td><td>AUR62025811-RA</td><td>AT3G63520.1 | Symbols: ATNCED1, ATCCD1, CCD1, NCED1 | carotenoid ...</td><td>877</td><td>0.0E+00</td><td>yes (Figure S5)</td><td>1/4 - CqCCD1</td></tr>
<tr><td></td><td></td><td>AT4G19170.1 | Symbols: NCED4, CCD4 | carotenoid cleavage dioxygen...</td><td>330</td><td>6.0E-106</td><td></td><td></td></tr>
<tr><td></td><td></td><td>AT1G30100.1 | Symbols: NCED5, ATNCED5 | nine-cis-epoxycarotenoid ...</td><td>323</td><td>4.0E-103</td><td></td><td></td></tr>
<tr><td></td><td></td><td>AT3G24220.1 | Symbols: NCED6, ATNCED6 | nine-cis-epoxycarotenoid ...</td><td>322</td><td>6.0E-103</td><td></td><td></td></tr>
<tr><td></td><td></td><td>AT1G78390.1 | Symbols: ATNCED9, NCED9 | NINE-CIS-EPOXYCAROTENOID ...</td><td>320</td><td>3.0E-101</td><td></td><td></td></tr>
<tr><td></td><td></td><td>AT3G14440.1 | Symbols: SIS7, ATNCED3, NCED3, STO1 | SALT TOLERANT...</td><td>310</td><td>4.0E-98</td><td></td><td></td></tr>
</table>

Primer design for PCR cloning of *C. album* sequences (Supplementary Table 1)

Sanger sequencing of *C. album* sequences and verification via BLAST analysis (Supplementary Figure 6) and phylogenetic trees (Figure 6A)

Primer design (Supplementary Table 2) for *C. album* seed RT-qPCR

Figure S4 continued next page…

E BLAST analysis of CYP707A genes to identify *Chenopodium quinoa* sequences as templates for primer design

Arabidopsis thaliana gene identifier	Gene	Protein length (aa)	BlastN vs predicted (Phytozome website)	Score	E-value	BlastX vs Chenopodium quinoa transcripts (Phytozome website)	Score	E-value
AT4G19230	CYP707A1	484	jgi:C_Quinoa_Scaffold_3973_392 Cquinoa|392|v1.0	233	1.0E-58	jgi:36319720 C_quinoa_v1_0|392|AUR62030408-RA|AUR62030408-RA|CYP7...	684	0.0E+00
			jgi:C_Quinoa_Scaffold_1280_392 Cquinoa|392|v1.0	224	5.0E-56	jgi:36294192 C_quinoa_v1_0|392|AUR62010485-RA|AUR62010485-RA|CYP7...	665	0.0E+00
			jgi:C_Quinoa_Scaffold_2493_392 Cquinoa|392|v1.0	126	4.0E-26	jgi:36321619 C_quinoa_v1_0|392|AUR62009542-RA|AUR62009542-RA|CYP7...	533	0.0E+00
						jgi:36292451 C_quinoa_v1_0|392|AUR62003877-RA|AUR62003877-RA|CYP7...	392	2.0E-121
AT2G29090	CYP707A2	482	jgi:C_Quinoa_Scaffold_2716_392 Cquinoa|392|v1.0	187	1.0E-44	jgi:36321619 C_quinoa_v1_0|392|AUR62009542-RA|AUR62009542-RA|CYP7...	618	0.0E+00
			jgi:C_Quinoa_Scaffold_2493_392 Cquinoa|392|v1.0	159	2.0E-36	jgi:36319720 C_quinoa_v1_0|392|AUR62030408-RA|AUR62030408-RA|CYP7...	529	0.0E+00
			jgi:C_Quinoa_Scaffold_4480_392 Cquinoa|392|v1.0	96	7.0E-17	jgi:36294192 C_quinoa_v1_0|392|AUR62010485-RA|AUR62010485-RA|CYP7...	523	0.0E+00
						jgi:36292451 C_quinoa_v1_0|392|AUR62003877-RA|AUR62003877-RA|CYP7...	344	8.0E-104
AT5G45340	CYP707A3	463	jgi:C_Quinoa_Scaffold_3973_392 Cquinoa|392|v1.0	211	1.0E-51	jgi:36319720 C_quinoa_v1_0|392|AUR62030408-RA|AUR62030408-RA|CYP7...	716	0.0E+00
			jgi:C_Quinoa_Scaffold_1280_392 Cquinoa|392|v1.0	201	6.0E-49	jgi:36294192 C_quinoa_v1_0|392|AUR62010485-RA|AUR62010485-RA|CYP7...	699	0.0E+00
			jgi:C_Quinoa_Scaffold_2493_392 Cquinoa|392|v1.0	115	7.0E-23	jgi:36321619 C_quinoa_v1_0|392|AUR62009542-RA|AUR62009542-RA|CYP7...	550	0.0E+00
						jgi:36292451 C_quinoa_v1_0|392|AUR62003877-RA|AUR62003877-RA|CYP7...	392	1.0E-121
AT3G19270	CYP707A4	443	jgi:C_Quinoa_Scaffold_2493_392 Cquinoa|392|v1.0	94	2.0E-16	jgi:36319720 C_quinoa_v1_0|392|AUR62030408-RA|AUR62030408-RA|CYP7...	507	7.0E-178
			jgi:C_Quinoa_Scaffold_2716_392 Cquinoa|392|v1.0	90	2.0E-15	jgi:36321619 C_quinoa_v1_0|392|AUR62009542-RA|AUR62009542-RA|CYP7...	495	1.0E-173
			jgi:C_Quinoa_Scaffold_3973_392 Cquinoa|392|v1.0	86	2.0E-14	jgi:36294192 C_quinoa_v1_0|392|AUR62010485-RA|AUR62010485-RA|CYP7...	490	2.0E-171
						jgi:36292451 C_quinoa_v1_0|392|AUR62003877-RA|AUR62003877-RA|CYP7...	448	3.0E-145
						jgi:36289290 C_quinoa_v1_0|392|AUR62001933-RA|AUR62001933-RA|CYP7...	321	1.0E-107

plus direct search for *C. quinoa* sequences (Phytozome)

Identified potential *C. quinoa* CYP707A sequences

Verification of *C. quinoia* CYP707A genes and transcript sequences

Primer design for PCR cloning of *C. album* sequences (Supplementary Table 1)

Sanger sequencing of *C. album* sequences and verification via BLAST analysis (Supplementary Figure 6) and phylogenetic trees (Figure 6B)

Primer design (Supplementary Table 2) for *C. album* seed RT-qPCR

Phytozome Chenopodium quinoia gene ID	Phytozome Chenopodium quinoia transcript	BLAST back to TAIR (BlastX using Chenopodium quinoa phytozome cds)	Score	E-value	DEG in Chenopodium quinoa seed transcriptome	Subgroup and name in phylogeny (Figure 7B)
AUR62030408	AUR62030408-RA	AT4G19230.1 | Symbols: CYP707A1 | "cytochrome P450, family 707, s...	716	0.0E+00	yes (Figure S5)	1/3 - CqCYP707A1-like
		AT5G45340.1 | Symbols: CYP707A3 | "cytochrome P450, family 707, s...	705	0.0E+00		
		AT4G19230.2 | Symbols: CYP707A1 | "cytochrome P450, family 707, s...	673	0.0E+00		
		AT5G45340.2 | Symbols: CYP707A3 | "cytochrome P450, family 707, s...	658	0.0E+00		
		AT2G29090.2 | Symbols: CYP707A2 | "cytochrome P450, family 707, s...	532	0.0E+00		
		AT2G29090.1 | Symbols: CYP707A2 | "cytochrome P450, family 707, s...	532	0.0E+00		
		AT3G19270.2 | Symbols: CYP707A4 | "cytochrome P450, family 707, s...	526	0.0E+00		
		AT3G19270.1 | Symbols: CYP707A4 | "cytochrome P450, family 707, s...	498	9.0E-175		
AUR62010485	AUR62010485-RA	AT4G19230.1 | Symbols: CYP707A1 | "cytochrome P450, family 707, s...	708	0.0E+00	no	1/3 - CqCYP707A1-like
		AT5G45340.1 | Symbols: CYP707A3 | "cytochrome P450, family 707, s...	699	0.0E+00		
		AT4G19230.2 | Symbols: CYP707A1 | "cytochrome P450, family 707, s...	665	0.0E+00		
		AT5G45340.2 | Symbols: CYP707A3 | "cytochrome P450, family 707, s...	652	0.0E+00		
		AT2G29090.2 | Symbols: CYP707A2 | "cytochrome P450, family 707, s...	533	0.0E+00		
		AT2G29090.1 | Symbols: CYP707A2 | "cytochrome P450, family 707, s...	533	0.0E+00		
		AT3G19270.2 | Symbols: CYP707A4 | "cytochrome P450, family 707, s...	519	0.0E+00		
		AT3G19270.1 | Symbols: CYP707A4 | "cytochrome P450, family 707, s...	491	3.0E-172		
AUR62009542	AUR62009542-RA	AT2G29090.2 | Symbols: CYP707A2 | "cytochrome P450, family 707, s...	630	0.0E+00	yes (Figure S5)	2 - CqCYP707A2-like
		AT2G29090.1 | Symbols: CYP707A2 | "cytochrome P450, family 707, s...	630	0.0E+00		
		AT4G19230.1 | Symbols: CYP707A1 | "cytochrome P450, family 707, s...	565	0.0E+00		
		AT2G29090.3 | Symbols: CYP707A2 | "cytochrome P450, family 707, s...	552	0.0E+00		
		AT5G45340.1 | Symbols: CYP707A3 | "cytochrome P450, family 707, s...	550	0.0E+00		
		AT4G19230.2 | Symbols: CYP707A1 | "cytochrome P450, family 707, s...	533	0.0E+00		
		AT3G19270.2 | Symbols: CYP707A4 | "cytochrome P450, family 707, s...	527	0.0E+00		
		AT5G45340.2 | Symbols: CYP707A3 | "cytochrome P450, family 707, s...	518	0.0E+00		
		AT3G19270.1 | Symbols: CYP707A4 | "cytochrome P450, family 707, s...	496	1.0E-174		
AUR62001756	AUR62001756-RA	AT2G29090.4 | Symbols: CYP707A2 | "cytochrome P450, family 707, s...	286	6.0E-95	yes (Figure S5)	2 - CqCYP707A2
		AT2G29090.2 | Symbols: CYP707A2 | "cytochrome P450, family 707, s...	286	3.0E-93		
		AT2G29090.1 | Symbols: CYP707A2 | "cytochrome P450, family 707, s...	286	3.0E-93		
		AT2G29090.3 | Symbols: CYP707A2 | "cytochrome P450, family 707, s...	275	8.0E-90		
		AT4G19230.1 | Symbols: CYP707A1 | "cytochrome P450, family 707, s...	246	1.0E-77		
		AT4G19230.2 | Symbols: CYP707A1 | "cytochrome P450, family 707, s...	246	2.0E-77		
		AT5G45340.1 | Symbols: CYP707A3 | "cytochrome P450, family 707, s...	241	8.0E-76		
		AT5G45340.2 | Symbols: CYP707A3 | "cytochrome P450, family 707, s...	238	1.0E-74		
		AT3G19270.1 | Symbols: CYP707A4 | "cytochrome P450, family 707, s...	234	2.0E-73		
		AT3G19270.2 | Symbols: CYP707A4 | "cytochrome P450, family 707, s...	234	6.0E-73		
		AT5G14400.2 | Symbols: CYP724A1 | "cytochrome P450, family 724, s...	139	2.0E-37		
AUR62001933	AUR62001933-RA	AT3G19270.1 | Symbols: CYP707A4 | "cytochrome P450, family 707, s...	322	1.0E-108	yes (Figure S5)	4 - CqCYP707A4-like
		AT3G19270.2 | Symbols: CYP707A4 | "cytochrome P450, family 707, s...	321	4.0E-108		
		AT4G19230.1 | Symbols: CYP707A1 | "cytochrome P450, family 707, s...	279	1.0E-91		
		AT4G19230.2 | Symbols: CYP707A1 | "cytochrome P450, family 707, s...	279	2.0E-91		
		AT5G45340.2 | Symbols: CYP707A3 | "cytochrome P450, family 707, s...	276	5.0E-91		
		AT5G45340.1 | Symbols: CYP707A3 | "cytochrome P450, family 707, s...	277	6.0E-91		
		AT2G29090.3 | Symbols: CYP707A2 | "cytochrome P450, family 707, s...	234	5.0E-75		
		AT2G29090.2 | Symbols: CYP707A2 | "cytochrome P450, family 707, s...	233	1.0E-73		
		AT2G29090.1 | Symbols: CYP707A2 | "cytochrome P450, family 707, s...	233	1.0E-73		
		AT2G29090.4 | Symbols: CYP707A2 | "cytochrome P450, family 707, s...	182	2.0E-55		
AUR62003877	AUR62003877-RA	AT3G19270.1 | Symbols: CYP707A4 | "cytochrome P450, family 707, s...	429	4.0E-138	no	4 - CqCYP707A4-like
		AT3G19270.2 | Symbols: CYP707A4 | "cytochrome P450, family 707, s...	429	8.0E-138		
		AT3G09040.4 | Symbols: MEF12 | mitochondrial RNA editing factor 1...	412	9.0E-125		
		AT3G09040.1 | Symbols: MEF12 | mitochondrial RNA editing factor 1...	412	9.0E-125		
		AT3G09040.3 | Symbols: MEF12 | mitochondrial RNA editing factor 1...	413	1.0E-124		
		AT3G09040.2 | Symbols: MEF12 | mitochondrial RNA editing factor 1...	413	1.0E-124		
		AT1G16480.1 | Symbols: no symbol available | no full name availab...	394	6.0E-119		
		AT4G19230.2 | Symbols: CYP707A1 | "cytochrome P450, family 707, s...	377	5.0E-118		

Supplementary Figure 4 | BLAST analysis of *Chenopodium quinoa* GA and ABA metabolism gene transcripts. To identify *Chenopodium* sequences the *C. quinoa* genome was mined and BLAST analyses conducted with the *A. thaliana* and *C. quinoa* transcript sequences via TAIR and Phytozome as presented in the workflow and tables. For further details and references see the main text.

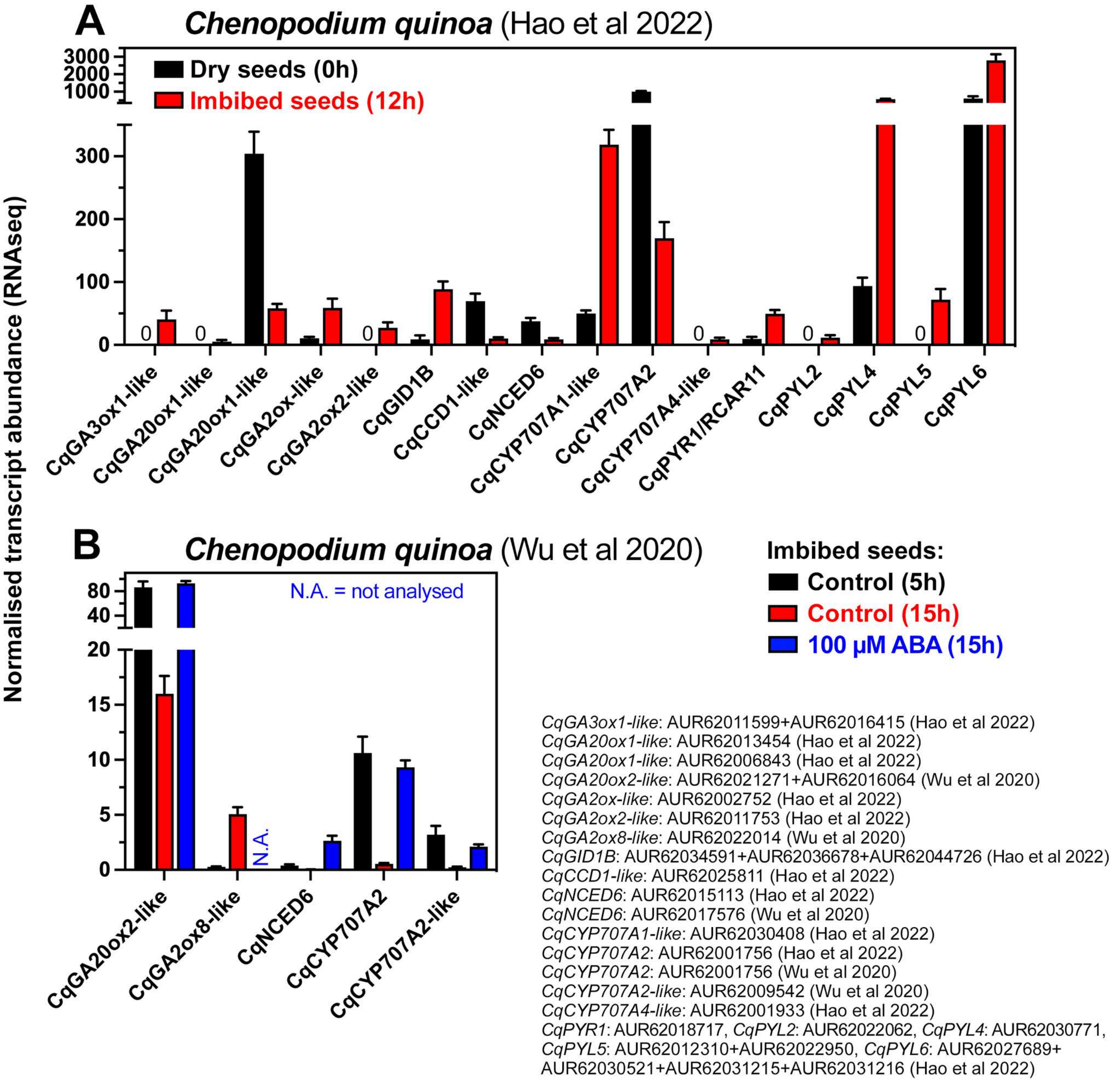

Supplementary Figure 5 | Transcript expression patterns of GA and ABA metabolism genes during *Chenopodium quinoa* seed germination. Differentially expressed genes from the published transcriptomes of Hao et al. (2022) and Wu et al. (2020) are presented. For further details and the references see the main text. Presented mean ± SEM values are the cumulative transcript abundances for the corresponding *C. quinoia* genes listed in the table. See Figures 5, 6, S4 and S7 for BLAST analyses and phylogenies of the *C. quinoia* sequences.

A BLAST analysis against *Chenopodium quinoa* of *Chenopodium album* sequences used as templates for RT-qPCR primer design

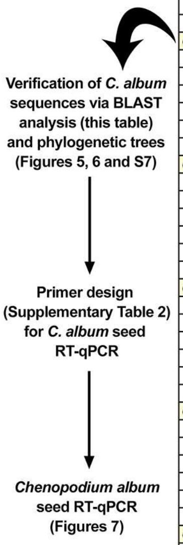

C. album gene name (sequence name)	Genebank accession number	Length (bp)*	BLASTX vs Phytozome (*Chenopodium album* versus *Chenopodium quinoa*)	Score	E-value	RT-qPCR results
CalGA20ox1 (EL36)	OQ360684	650	jgi:36319174 C_quinoa_v1_0|392|AUR62004269-RA|AUR62004269-RA|GA20ox	227	1.0E-74	none
			jgi:36311734 C_quinoa_v1_0|392|AUR62033388-RA|AUR62033388-RA|GA20ox	226	6.0E-73	
			jgi:36318333 C_quinoa_v1_0|392|AUR62021271-RA|AUR62021271-RA|GA20ox	182	8.0E-56	
			jgi:36288619 C_quinoa_v1_0|392|AUR62016064-RA|AUR62016064-RA|GA20ox	179	1.0E-54	
			jgi:36292046 C_quinoa_v1_0|392|AUR62013454-RA|AUR62013454-RA|GA20ox	142	1.0E-40	
			jgi:36285550 C_quinoa_v1_0|392|AUR62007685-RA|AUR62007685-RA|GA20ox	141	2.0E-40	
CalGA20ox2 (EL39)	OQ351935	374	jgi:36288619 C_quinoa_v1_0|392|AUR62016064-RA|AUR62016064-RA|GA20ox	196	2.0E-62	Figure 7
			jgi:36318333 C_quinoa_v1_0|392|AUR62021271-RA|AUR62021271-RA|GA20ox	193	1.0E-61	
			jgi:36319174 C_quinoa_v1_0|392|AUR62004269-RA|AUR62004269-RA|GA20ox	118	2.0E-33	
			jgi:36311734 C_quinoa_v1_0|392|AUR62033388-RA|AUR62033388-RA|GA20ox	120	2.0E-33	
			jgi:36302756 C_quinoa_v1_0|392|AUR62006843-RA|AUR62006843-RA|GA20ox	96	1.0E-24	
			jgi:36298246 C_quinoa_v1_0|392|AUR62000497-RA|AUR62000497-RA|GA20ox	96	2.0E-24	
CalGA20ox3 (EL37)	OQ351936	311	jgi:36292046 C_quinoa_v1_0|392|AUR62013454-RA|AUR62013454-RA|GA20ox	217	3.0E-69	Figure 7
			jgi:36285550 C_quinoa_v1_0|392|AUR62007685-RA|AUR62007685-RA|GA20ox	147	1.0E-42	
			jgi:36311734 C_quinoa_v1_0|392|AUR62033388-RA|AUR62033388-RA|GA20ox	92	2.0E-21	
			jgi:36319174 C_quinoa_v1_0|392|AUR62004269-RA|AUR62004269-RA|GA20ox	91	2.0E-21	
			jgi:36302756 C_quinoa_v1_0|392|AUR62006843-RA|AUR62006843-RA|GA20ox	90	5.0E-21	
			jgi:36298246 C_quinoa_v1_0|392|AUR62000497-RA|AUR62000497-RA|GA20ox	87	5.0E-20	
CalGA3ox1/2 (EL51)	OQ360685	654	jgi:36290230 C_quinoa_v1_0|392|AUR62016415-RA|AUR62016415-RA|LE: GA3c	196	7.0E-64	Figure 7
			jgi:36308453 C_quinoa_v1_0|392|AUR62011599-RA|AUR62011599-RA|LE: GA3c	193	5.0E-60	
			jgi:36305840 C_quinoa_v1_0|392|AUR62032368-RA|AUR62032368-RA|LE: GA3c	119	9.0E-32	
			jgi:36284702 C_quinoa_v1_0|392|AUR62024851-RA|AUR62024851-RA|LE: GA3c	117	5.0E-31	
			jgi:36294240 C_quinoa_v1_0|392|AUR62038695-RA|AUR62038695-RA|GA3ox...	110	3.0E-30	
			jgi:36307459 C_quinoa_v1_0|392|AUR62032673-RA|AUR62032673-RA|GA3ox...	112	3.0E-30	
CalGA3ox3/4 (EL12)	OQ351937	224	jgi:36320364 C_quinoa_v1_0|392|AUR62032075-RA|AUR62032075-RA|GA3ox...	116	5.0E-35	Figure 7
			jgi:36307459 C_quinoa_v1_0|392|AUR62032673-RA|AUR62032673-RA|GA3ox...	111	4.0E-32	
			jgi:36294240 C_quinoa_v1_0|392|AUR62038695-RA|AUR62038695-RA|GA3ox...	92	2.0E-25	
			jgi:36294241 C_quinoa_v1_0|392|AUR62038693-RA|AUR62038693-RA|LE: GA3c	94	1.0E-24	
			jgi:36305840 C_quinoa_v1_0|392|AUR62032368-RA|AUR62032368-RA|LE: GA3c	92	7.0E-24	
			jgi:36294257 C_quinoa_v1_0|392|AUR62038694-RA|AUR62038694-RA|ACO2...	88	9.0E-23	
CalGA3ox1/4 (EL13)	OQ351938	184	jgi:36320333 C_quinoa_v1_0|392|AUR62032076-RA|AUR62032076-RA|LE: GA3c	126	7.0E-39	Figure 7
			jgi:36284702 C_quinoa_v1_0|392|AUR62024851-RA|AUR62024851-RA|LE: GA3c	104	1.0E-28	
			jgi:36305840 C_quinoa_v1_0|392|AUR62032368-RA|AUR62032368-RA|LE: GA3c	103	2.0E-28	
			jgi:36294241 C_quinoa_v1_0|392|AUR62038693-RA|AUR62038693-RA|LE: GA3c	102	7.0E-28	
			jgi:36294257 C_quinoa_v1_0|392|AUR62038694-RA|AUR62038694-RA|ACO2...	79	2.0E-19	
			jgi:36301471 C_quinoa_v1_0|392|AUR62035812-RA|AUR62035812-RA|SRG1...	70	5.0E-16	
CalGA2ox2 (EL45)	OQ360686	180	jgi:36301611 C_quinoa_v1_0|392|AUR62002752-RA|AUR62002752-RA|GA2ox...	100	4.0E-27	Figure 7
			jgi:36304895 C_quinoa_v1_0|392|AUR62030199-RA|AUR62030199-RA|GA2ox...	93	2.0E-25	
			jgi:36310380 C_quinoa_v1_0|392|AUR62024597-RA|AUR62024597-RA|GA2ox...	44	7.0E-07	
			jgi:36311284 C_quinoa_v1_0|392|AUR62011753-RA|AUR62011753-RA|GA2ox...	44	2.0E-06	
CalNCED3 (EL02)	OQ351932	1053	jgi:36303079 C_quinoa_v1_0|392|AUR62037951-RA|AUR62037951-RA|NCED...	425	7.0E-146	Figure 7
			jgi:36306192 C_quinoa_v1_0|392|AUR62043087-RA|AUR62043087-RA|NCED...	420	6.0E-144	
			jgi:36304873 C_quinoa_v1_0|392|AUR62030185-RA|AUR62030185-RA|NCED...	318	3.0E-140	
			jgi:36301918 C_quinoa_v1_0|392|AUR62002735-RA|AUR62002735-RA|NCED...	281	8.0E-136	
			jgi:36318712 C_quinoa_v1_0|392|AUR62015113-RA|AUR62015113-RA|NCED...	266	9.0E-109	
			jgi:36291573 C_quinoa_v1_0|392|AUR62017576-RA|AUR62017576-RA|NCED...	266	1.0E-86	
			jgi:36292536 C_quinoa_v1_0|392|AUR62003579-RA|AUR62003579-RA|NCED...	223	1.0E-68	
CalNCED5 (EL49)	OQ360683	981	jgi:36304873 C_quinoa_v1_0|392|AUR62030185-RA|AUR62030185-RA|NCED...	621	0.0E+00	Figure 7
			jgi:36301918 C_quinoa_v1_0|392|AUR62002735-RA|AUR62002735-RA|NCED...	613	0.0E+00	
			jgi:36292536 C_quinoa_v1_0|392|AUR62003579-RA|AUR62003579-RA|NCED...	506	1.0E-179	
			jgi:36306192 C_quinoa_v1_0|392|AUR62043087-RA|AUR62043087-RA|NCED...	500	8.0E-176	
			jgi:36303079 C_quinoa_v1_0|392|AUR62037951-RA|AUR62037951-RA|NCED...	492	2.0E-172	
			jgi:36318712 C_quinoa_v1_0|392|AUR62015113-RA|AUR62015113-RA|NCED...	411	3.0E-142	
CalNCED6A (EL07)	OQ351933	821	jgi:36291573 C_quinoa_v1_0|392|AUR62017576-RA|AUR62017576-RA|NCED...	449	7.0E-160	Figure 7
			jgi:36318712 C_quinoa_v1_0|392|AUR62015113-RA|AUR62015113-RA|NCED...	439	3.0E-154	
			jgi:36304873 C_quinoa_v1_0|392|AUR62030185-RA|AUR62030185-RA|NCED...	293	3.0E-97	
			jgi:36306192 C_quinoa_v1_0|392|AUR62043087-RA|AUR62043087-RA|NCED...	292	2.0E-95	
			jgi:36303079 C_quinoa_v1_0|392|AUR62037951-RA|AUR62037951-RA|NCED...	292	2.0E-95	
			jgi:36301918 C_quinoa_v1_0|392|AUR62002735-RA|AUR62002735-RA|NCED...	261	9.0E-84	
CalNCED6B (EL16)	OQ351934	307	jgi:36291595 C_quinoa_v1_0|392|AUR62017575-RA|AUR62017575-RA|NCED...	205	5.0E-70	Figure 7
			jgi:36318712 C_quinoa_v1_0|392|AUR62015113-RA|AUR62015113-RA|NCED...	91	1.0E-22	
			jgi:36301918 C_quinoa_v1_0|392|AUR62002735-RA|AUR62002735-RA|NCED...	74	2.0E-16	
			jgi:36304873 C_quinoa_v1_0|392|AUR62030185-RA|AUR62030185-RA|NCED...	72	6.0E-16	
			jgi:36292536 C_quinoa_v1_0|392|AUR62003579-RA|AUR62003579-RA|NCED...	70	3.0E-15	
			jgi:36303079 C_quinoa_v1_0|392|AUR62037951-RA|AUR62037951-RA|NCED...	68	2.0E-14	
CalCYP707A1/3 (EL61)	OQ351929	411	jgi:36319720 C_quinoa_v1_0|392|AUR62030408-RA|AUR62030408-RA|CYP707A	135	2.0E-38	Figure 7
			jgi:36321619 C_quinoa_v1_0|392|AUR62009542-RA|AUR62009542-RA|CYP707A	115	4.0E-31	
			jgi:36294192 C_quinoa_v1_0|392|AUR62010485-RA|AUR62010485-RA|CYP707A	111	1.0E-29	
			jgi:36314201 C_quinoa_v1_0|392|AUR62001756-RA|AUR62001756-RA|CYP707A	88	3.0E-21	
			jgi:36292451 C_quinoa_v1_0|392|AUR62003877-RA|AUR62003877-RA|CYP707A	78	2.0E-17	
			jgi:36310554 C_quinoa_v1_0|392|AUR62019994-RA|AUR62019994-RA|KAO1...	68	5.0E-14	
CalCYP707A2 (EL65)	OQ351930	465	jgi:36321619 C_quinoa_v1_0|392|AUR62009542-RA|AUR62009542-RA|CYP707A	247	1.0E-81	Figure 7
			jgi:36319720 C_quinoa_v1_0|392|AUR62030408-RA|AUR62030408-RA|CYP707A	140	4.0E-40	
			jgi:36294192 C_quinoa_v1_0|392|AUR62010485-RA|AUR62010485-RA|CYP707A	126	7.0E-35	
			jgi:36292451 C_quinoa_v1_0|392|AUR62003877-RA|AUR62003877-RA|CYP707A	114	1.0E-29	
			jgi:36314201 C_quinoa_v1_0|392|AUR62001756-RA|AUR62001756-RA|CYP707A	97	2.0E-24	
			jgi:36289290 C_quinoa_v1_0|392|AUR62001933-RA|AUR62001933-RA|CYP707A	89	6.0E-22	
CalCYP707A4 (EL67)	OQ351931	511	jgi:36289290 C_quinoa_v1_0|392|AUR62001933-RA|AUR62001933-RA|CYP707A	340	2.0E-120	Figure 7
			jgi:36292451 C_quinoa_v1_0|392|AUR62003877-RA|AUR62003877-RA|CYP707A	349	5.0E-113	
			jgi:36319720 C_quinoa_v1_0|392|AUR62030408-RA|AUR62030408-RA|CYP707A	146	6.0E-42	
			jgi:36294192 C_quinoa_v1_0|392|AUR62010485-RA|AUR62010485-RA|CYP707A	137	9.0E-39	
			jgi:36321619 C_quinoa_v1_0|392|AUR62009542-RA|AUR62009542-RA|CYP707A	127	4.0E-35	
			jgi:36293993 C_quinoa_v1_0|392|AUR62010642-RA|AUR62010642-RA|CYP8...	83	8.0E-19	

* Length of deposited *C. album* sequence in Genebank

Figure S6 continued next page…

B BLAST analysis against *Arabidopsis thaliana* of *Chenopodium album* sequences used as templates for RT-qPCR primer design

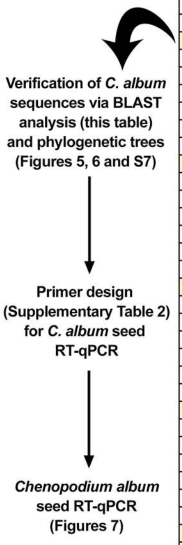

<table>
<tr><th>C. album gene name (sequence name)</th><th>Genebank accession number</th><th>Length (bp)*</th><th>BLASTX vs TAIR (Chenopodium album versus Arabidopsis thaliana)</th><th>Score</th><th>E-value</th><th>RT-qPCR results</th></tr>
<tr><td></td><td></td><td></td><td></td><td></td><td></td><td></td></tr>
<tr><td>CalGA20ox1 (EL36)</td><td>OQ360684</td><td>650</td><td>AT4G25420.2 | Symbols: ATGA20OX1, AT2301, GA5, GA20OX1 | ARABIDOP...</td><td>197</td><td>2.0E-62</td><td>none</td></tr>
<tr><td></td><td></td><td></td><td>AT5G51810.2 | Symbols: AT2353, ATGA20OX2, GA20OX2 | gibberellin 2...</td><td>194</td><td>2.0E-61</td><td></td></tr>
<tr><td></td><td></td><td></td><td>AT4G25420.1 | Symbols: ATGA20OX1, AT2301, GA5, GA20OX1 | ARABIDOP...</td><td>196</td><td>3.0E-61</td><td></td></tr>
<tr><td></td><td></td><td></td><td>AT5G51810.1 | Symbols: AT2353, ATGA20OX2, GA20OX2 | gibberellin 2...</td><td>192</td><td>1.0E-59</td><td></td></tr>
<tr><td></td><td></td><td></td><td>AT5G07200.1 | Symbols: ATGA20OX3, YAP169, GA20OX3 | gibberellin 2...</td><td>180</td><td>6.0E-55</td><td></td></tr>
<tr><td></td><td></td><td></td><td>AT1G60980.1 | Symbols: GA20OX4, ATGA20OX4 | gibberellin 20-oxidas...</td><td>169</td><td>8.0E-51</td><td></td></tr>
<tr><td></td><td></td><td></td><td></td><td></td><td></td><td></td></tr>
<tr><td>CalGA20ox2 (EL39)</td><td>OQ351935</td><td>374</td><td>AT5G51810.2 | Symbols: AT2353, ATGA20OX2, GA20OX2 | gibberellin 2...</td><td>137</td><td>1.0E-40</td><td>Figure 7</td></tr>
<tr><td></td><td></td><td></td><td>AT5G51810.1 | Symbols: AT2353, ATGA20OX2, GA20OX2 | gibberellin 2...</td><td>137</td><td>6.0E-40</td><td></td></tr>
<tr><td></td><td></td><td></td><td>AT4G25420.2 | Symbols: ATGA20OX1, AT2301, GA5, GA20OX1 | ARABIDOP...</td><td>123</td><td>5.0E-35</td><td></td></tr>
<tr><td></td><td></td><td></td><td>AT4G25420.1 | Symbols: ATGA20OX1, AT2301, GA5, GA20OX1 | ARABIDOP...</td><td>123</td><td>2.0E-34</td><td></td></tr>
<tr><td></td><td></td><td></td><td>AT5G07200.1 | Symbols: ATGA20OX3, YAP169, GA20OX3 | gibberellin 2...</td><td>113</td><td>1.0E-30</td><td></td></tr>
<tr><td></td><td></td><td></td><td>AT1G60980.1 | Symbols: GA20OX4, ATGA20OX4 | gibberellin 20-oxidas...</td><td>112</td><td>2.0E-30</td><td></td></tr>
<tr><td></td><td></td><td></td><td></td><td></td><td></td><td></td></tr>
<tr><td>CalGA20ox3 (EL37)</td><td>OQ351936</td><td>311</td><td>AT4G25420.2 | Symbols: ATGA20OX1, AT2301, GA5, GA20OX1 | ARABIDOP...</td><td>96</td><td>5.0E-23</td><td>Figure 7</td></tr>
<tr><td></td><td></td><td></td><td>AT4G25420.1 | Symbols: ATGA20OX1, AT2301, GA5, GA20OX1 | ARABIDOP...</td><td>96</td><td>1.0E-22</td><td></td></tr>
<tr><td></td><td></td><td></td><td>AT5G51810.2 | Symbols: AT2353, ATGA20OX2, GA20OX2 | gibberellin 2...</td><td>90</td><td>4.0E-21</td><td></td></tr>
<tr><td></td><td></td><td></td><td>AT5G51810.1 | Symbols: AT2353, ATGA20OX2, GA20OX2 | gibberellin 2...</td><td>90</td><td>1.0E-20</td><td></td></tr>
<tr><td></td><td></td><td></td><td>AT5G07200.1 | Symbols: ATGA20OX3, YAP169, GA20OX3 | gibberellin 2...</td><td>78</td><td>2.0E-16</td><td></td></tr>
<tr><td></td><td></td><td></td><td>AT1G60980.1 | Symbols: GA20OX4, ATGA20OX4 | gibberellin 20-oxidas...</td><td>76</td><td>2.0E-15</td><td></td></tr>
<tr><td></td><td></td><td></td><td></td><td></td><td></td><td></td></tr>
<tr><td>CalGA3ox1/2 (EL51)</td><td>OQ360685</td><td>654</td><td>AT1G80340.1 | Symbols: GA4H, GA3OX2, ATGA3OX2 | ARABIDOPSIS THALI...</td><td>123</td><td>2.0E-33</td><td>Figure 7</td></tr>
<tr><td></td><td></td><td></td><td>AT1G15550.1 | Symbols: GA4, ATGA3OX1, GA3OX1 | ARABIDOPSIS THALIA...</td><td>117</td><td>3.0E-31</td><td></td></tr>
<tr><td></td><td></td><td></td><td>AT1G80330.1 | Symbols: GA3OX4, ATGA3OX4 | gibberellin 3-oxidase 4...</td><td>110</td><td>2.0E-28</td><td></td></tr>
<tr><td></td><td></td><td></td><td>AT4G21690.1 | Symbols: ATGA3OX3, GA3OX3 | ARABIDOPSIS THALIANA GI...</td><td>106</td><td>5.0E-27</td><td></td></tr>
<tr><td></td><td></td><td></td><td>AT1G78550.1 | Symbols: no symbol available | no full name availab...</td><td>61</td><td>7.0E-11</td><td></td></tr>
<tr><td></td><td></td><td></td><td>AT1G17010.1 | Symbols: no symbol available | no full name availab...</td><td>60</td><td>3.0E-10</td><td></td></tr>
<tr><td></td><td></td><td></td><td></td><td></td><td></td><td></td></tr>
<tr><td>CalGA3ox3/4 (EL12)</td><td>OQ351937</td><td>224</td><td>AT1G80330.1 | Symbols: GA3OX4, ATGA3OX4 | gibberellin 3-oxidase 4...</td><td>52</td><td>3.0E-09</td><td>Figure 7</td></tr>
<tr><td></td><td></td><td></td><td>AT1G80340.1 | Symbols: GA4H, GA3OX2, ATGA3OX2 | ARABIDOPSIS THALI...</td><td>45</td><td>7.0E-07</td><td></td></tr>
<tr><td></td><td></td><td></td><td>AT4G21690.1 | Symbols: ATGA3OX3, GA3OX3 | ARABIDOPSIS THALIANA GI...</td><td>44</td><td>1.0E-06</td><td></td></tr>
<tr><td></td><td></td><td></td><td>AT1G15550.1 | Symbols: GA4, ATGA3OX1, GA3OX1 | ARABIDOPSIS THALIA...</td><td>44</td><td>2.0E-06</td><td></td></tr>
<tr><td></td><td></td><td></td><td>AT3G19010.2 | Symbols: no symbol available | no full name availab...</td><td>37</td><td>4.0E-04</td><td></td></tr>
<tr><td></td><td></td><td></td><td>AT2G34555.1 | Symbols: GA2OX3, ATGA2OX3 | gibberellin 2-oxidase 3...</td><td>37</td><td>6.0E-04</td><td></td></tr>
<tr><td></td><td></td><td></td><td></td><td></td><td></td><td></td></tr>
<tr><td>CalGA3ox1/4 (EL13)</td><td>OQ351938</td><td>184</td><td>AT1G80330.1 | Symbols: GA3OX4, ATGA3OX4 | gibberellin 3-oxidase 4...</td><td>73</td><td>4.0E-17</td><td>Figure 7</td></tr>
<tr><td></td><td></td><td></td><td>AT4G25310.1 | Symbols: no symbol available | no full name availab...</td><td>72</td><td>7.0E-17</td><td></td></tr>
<tr><td></td><td></td><td></td><td>AT1G17020.1 | Symbols: ATSRG1, SRG1 | SENESCENCE-RELATED GENE 1, ...</td><td>71</td><td>4.0E-16</td><td></td></tr>
<tr><td></td><td></td><td></td><td>AT4G21200.1 | Symbols: ATGA2OX8, GA2OX8 | gibberellin 2-oxidase 8...</td><td>70</td><td>6.0E-16</td><td></td></tr>
<tr><td></td><td></td><td></td><td>AT1G15550.1 | Symbols: GA4, ATGA3OX1, GA3OX1 | ARABIDOPSIS THALIA...</td><td>70</td><td>7.0E-16</td><td></td></tr>
<tr><td></td><td></td><td></td><td>AT4G25300.2 | Symbols: no symbol available | no full name availab...</td><td>69</td><td>9.0E-16</td><td></td></tr>
<tr><td></td><td></td><td></td><td></td><td></td><td></td><td></td></tr>
<tr><td>CalGA2ox2 (EL45)</td><td>OQ360686</td><td>180</td><td>AT1G30040.2 | Symbols: ATGA2OX2, GA2OX2 | gibberellin 2-oxidase, ...</td><td>57</td><td>3.0E-11</td><td>Figure 7</td></tr>
<tr><td></td><td></td><td></td><td>AT1G30040.1 | Symbols: ATGA2OX2, GA2OX2 | gibberellin 2-oxidase, ...</td><td>57</td><td>3.0E-11</td><td></td></tr>
<tr><td></td><td></td><td></td><td>AT2G34555.1 | Symbols: GA2OX3, ATGA2OX3 | gibberellin 2-oxidase 3...</td><td>52</td><td>9.0E-10</td><td></td></tr>
<tr><td></td><td></td><td></td><td>AT1G78440.1 | Symbols: GA2OX1, ATGA2OX1 | gibberellin 2-oxidase 1...</td><td>52</td><td>1.0E-09</td><td></td></tr>
<tr><td></td><td></td><td></td><td>AT1G02400.2 | Symbols: ATGA2OX6, DTA1, ATGA2OX4, GA2OX6 | Arabido...</td><td>51</td><td>3.0E-09</td><td></td></tr>
<tr><td></td><td></td><td></td><td>AT1G02400.1 | Symbols: ATGA2OX6, DTA1, ATGA2OX4, GA2OX6 | Arabido...</td><td>51</td><td>3.0E-09</td><td></td></tr>
<tr><td></td><td></td><td></td><td></td><td></td><td></td><td></td></tr>
<tr><td>CalNCED3 (EL02)</td><td>OQ351932</td><td>1053</td><td>AT1G30100.1 | Symbols: NCED5, ATNCED5 | nine-cis-epoxycarotenoid ...</td><td>325</td><td>8.0E-154</td><td>Figure 7</td></tr>
<tr><td></td><td></td><td></td><td>AT3G14440.1 | Symbols: SIS7, ATNCED3, NCED3, STO1 | SALT TOLERANT...</td><td>334</td><td>1.0E-151</td><td></td></tr>
<tr><td></td><td></td><td></td><td>AT1G78390.1 | Symbols: ATNCED9, NCED9 | NINE-CIS-EPOXYCAROTENOID ...</td><td>325</td><td>3.0E-147</td><td></td></tr>
<tr><td></td><td></td><td></td><td>AT4G18350.1 | Symbols: ATNCED2, NCED2 | NINE-CIS-EPOXYCAROTENOID ...</td><td>320</td><td>4.0E-143</td><td></td></tr>
<tr><td></td><td></td><td></td><td>AT3G24220.1 | Symbols: NCED6, ATNCED6 | nine-cis-epoxycarotenoid ...</td><td>280</td><td>2.0E-112</td><td></td></tr>
<tr><td></td><td></td><td></td><td>AT4G19170.1 | Symbols: NCED4, CCD4 | carotenoid cleavage dioxygen...</td><td>169</td><td>6.0E-64</td><td></td></tr>
<tr><td></td><td></td><td></td><td>AT3G63520.1 | Symbols: ATNCED1, ATCCD1, CCD1, NCED1 | carotenoid ...</td><td>157</td><td>7.0E-54</td><td></td></tr>
<tr><td></td><td></td><td></td><td></td><td></td><td></td><td></td></tr>
<tr><td>CalNCED5 (EL49)</td><td>OQ360683</td><td>981</td><td>AT3G14440.1 | Symbols: SIS7, ATNCED3, NCED3, STO1 | SALT TOLERANT...</td><td>523</td><td>0.0E+00</td><td>Figure 7</td></tr>
<tr><td></td><td></td><td></td><td>AT1G30100.1 | Symbols: NCED5, ATNCED5 | nine-cis-epoxycarotenoid ...</td><td>512</td><td>2.0E-180</td><td></td></tr>
<tr><td></td><td></td><td></td><td>AT1G78390.1 | Symbols: ATNCED9, NCED9 | NINE-CIS-EPOXYCAROTENOID ...</td><td>513</td><td>4.0E-180</td><td></td></tr>
<tr><td></td><td></td><td></td><td>AT4G18350.1 | Symbols: ATNCED2, NCED2 | NINE-CIS-EPOXYCAROTENOID ...</td><td>504</td><td>1.0E-177</td><td></td></tr>
<tr><td></td><td></td><td></td><td>AT3G24220.1 | Symbols: NCED6, ATNCED6 | nine-cis-epoxycarotenoid ...</td><td>415</td><td>2.0E-142</td><td></td></tr>
<tr><td></td><td></td><td></td><td>AT3G63520.1 | Symbols: ATNCED1, ATCCD1, CCD1, NCED1 | carotenoid ...</td><td>224</td><td>5.0E-69</td><td></td></tr>
<tr><td></td><td></td><td></td><td></td><td></td><td></td><td></td></tr>
<tr><td>CalNCED6A (EL07)</td><td>OQ351933</td><td>821</td><td>AT3G24220.1 | Symbols: NCED6, ATNCED6 | nine-cis-epoxycarotenoid ...</td><td>322</td><td>4.0E-107</td><td>Figure 7</td></tr>
<tr><td></td><td></td><td></td><td>AT3G14440.1 | Symbols: SIS7, ATNCED3, NCED3, STO1 | SALT TOLERANT...</td><td>310</td><td>4.0E-102</td><td></td></tr>
<tr><td></td><td></td><td></td><td>AT1G78390.1 | Symbols: ATNCED9, NCED9 | NINE-CIS-EPOXYCAROTENOID ...</td><td>303</td><td>5.0E-99</td><td></td></tr>
<tr><td></td><td></td><td></td><td>AT4G18350.1 | Symbols: ATNCED2, NCED2 | NINE-CIS-EPOXYCAROTENOID ...</td><td>298</td><td>1.0E-97</td><td></td></tr>
<tr><td></td><td></td><td></td><td>AT1G30100.1 | Symbols: NCED5, ATNCED5 | nine-cis-epoxycarotenoid ...</td><td>284</td><td>4.0E-92</td><td></td></tr>
<tr><td></td><td></td><td></td><td>AT3G63520.1 | Symbols: ATNCED1, ATCCD1, CCD1, NCED1 | carotenoid ...</td><td>164</td><td>6.0E-47</td><td></td></tr>
<tr><td></td><td></td><td></td><td></td><td></td><td></td><td></td></tr>
<tr><td>CalNCED6B (EL16)</td><td>OQ351934</td><td>307</td><td>AT3G24220.1 | Symbols: NCED6, ATNCED6 | nine-cis-epoxycarotenoid ...</td><td>73</td><td>3.0E-16</td><td>Figure 7</td></tr>
<tr><td></td><td></td><td></td><td>AT4G18350.1 | Symbols: ATNCED2, NCED2 | NINE-CIS-EPOXYCAROTENOID ...</td><td>69</td><td>1.0E-14</td><td></td></tr>
<tr><td></td><td></td><td></td><td>AT1G78390.1 | Symbols: ATNCED9, NCED9 | NINE-CIS-EPOXYCAROTENOID ...</td><td>68</td><td>1.0E-14</td><td></td></tr>
<tr><td></td><td></td><td></td><td>AT1G30100.1 | Symbols: NCED5, ATNCED5 | nine-cis-epoxycarotenoid ...</td><td>66</td><td>1.0E-13</td><td></td></tr>
<tr><td></td><td></td><td></td><td>AT3G14440.1 | Symbols: SIS7, ATNCED3, NCED3, STO1 | SALT TOLERANT...</td><td>58</td><td>5.0E-11</td><td></td></tr>
<tr><td></td><td></td><td></td><td>AT4G19170.1 | Symbols: NCED4, CCD4 | carotenoid cleavage dioxygen...</td><td>46</td><td>7.0E-07</td><td></td></tr>
<tr><td></td><td></td><td></td><td></td><td></td><td></td><td></td></tr>
<tr><td>CalCYP707A1/3 (EL61)</td><td>OQ351929</td><td>411</td><td>AT5G45340.2 | Symbols: CYP707A3 | "cytochrome P450, family 707, s...</td><td>125</td><td>9.0E-35</td><td>Figure 7</td></tr>
<tr><td></td><td></td><td></td><td>AT5G45340.1 | Symbols: CYP707A3 | "cytochrome P450, family 707, s...</td><td>125</td><td>1.0E-34</td><td></td></tr>
<tr><td></td><td></td><td></td><td>AT4G19230.1 | Symbols: CYP707A1 | "cytochrome P450, family 707, s...</td><td>121</td><td>3.0E-33</td><td></td></tr>
<tr><td></td><td></td><td></td><td>AT2G29090.3 | Symbols: CYP707A2 | "cytochrome P450, family 707, s...</td><td>120</td><td>4.0E-33</td><td></td></tr>
<tr><td></td><td></td><td></td><td>AT4G19230.2 | Symbols: CYP707A1 | "cytochrome P450, family 707, s...</td><td>120</td><td>1.0E-32</td><td></td></tr>
<tr><td></td><td></td><td></td><td>AT2G29090.2 | Symbols: CYP707A2 | "cytochrome P450, family 707, s...</td><td>119</td><td>3.0E-32</td><td></td></tr>
<tr><td></td><td></td><td></td><td></td><td></td><td></td><td></td></tr>
<tr><td>CalCYP707A2 (EL65)</td><td>OQ351930</td><td>465</td><td>AT2G29090.3 | Symbols: CYP707A2 | "cytochrome P450, family 707, s...</td><td>158</td><td>2.0E-47</td><td>Figure 7</td></tr>
<tr><td></td><td></td><td></td><td>AT2G29090.2 | Symbols: CYP707A2 | "cytochrome P450, family 707, s...</td><td>158</td><td>2.0E-46</td><td></td></tr>
<tr><td></td><td></td><td></td><td>AT2G29090.1 | Symbols: CYP707A2 | "cytochrome P450, family 707, s...</td><td>158</td><td>2.0E-46</td><td></td></tr>
<tr><td></td><td></td><td></td><td>AT4G19230.1 | Symbols: CYP707A1 | "cytochrome P450, family 707, s...</td><td>151</td><td>5.0E-44</td><td></td></tr>
<tr><td></td><td></td><td></td><td>AT4G19230.2 | Symbols: CYP707A1 | "cytochrome P450, family 707, s...</td><td>151</td><td>5.0E-44</td><td></td></tr>
<tr><td></td><td></td><td></td><td>AT5G45340.2 | Symbols: CYP707A3 | "cytochrome P450, family 707, s...</td><td>146</td><td>2.0E-42</td><td></td></tr>
<tr><td></td><td></td><td></td><td></td><td></td><td></td><td></td></tr>
<tr><td>CalCYP707A4 (EL67)</td><td>OQ351931</td><td>511</td><td>AT3G19270.1 | Symbols: CYP707A4 | "cytochrome P450, family 707, s...</td><td>200</td><td>6.0E-63</td><td>Figure 7</td></tr>
<tr><td></td><td></td><td></td><td>AT3G19270.2 | Symbols: CYP707A4 | "cytochrome P450, family 707, s...</td><td>200</td><td>1.0E-62</td><td></td></tr>
<tr><td></td><td></td><td></td><td>AT4G19230.1 | Symbols: CYP707A1 | "cytochrome P450, family 707, s...</td><td>163</td><td>2.0E-48</td><td></td></tr>
<tr><td></td><td></td><td></td><td>AT4G19230.2 | Symbols: CYP707A1 | "cytochrome P450, family 707, s...</td><td>163</td><td>2.0E-48</td><td></td></tr>
<tr><td></td><td></td><td></td><td>AT5G45340.2 | Symbols: CYP707A3 | "cytochrome P450, family 707, s...</td><td>161</td><td>7.0E-48</td><td></td></tr>
<tr><td></td><td></td><td></td><td>AT5G45340.1 | Symbols: CYP707A3 | "cytochrome P450, family 707, s...</td><td>161</td><td>9.0E-48</td><td></td></tr>
</table>

* Length of deposited *C. album* sequence in Genebank

Supplementary Figure 6 | BLAST analysis of identified *Chenopodium album* GA and ABA metabolism gene sequences against *Chenopodium quinoa* **(A)** and *Arabidopsis thaliana* **(B)**. PCR cloned *C. album* sequences EL36 (*CalGA20ox1*), EL39 (*CalGA20ox2*), EL37 (*CalGA20ox3*), EL51 (*CalGA3ox1/2*), EL12 (*CalGA3ox3/4*), EL13 (*CalGA3ox1/4*), EL45 (*CalGA2ox2*), EL02 (*CalNCED3*), EL02 (*CalNCED3*), EL49 (*CalNCED5*), EL07 (*CalNCED6A*), EL16 (*CalNCED6B*), EL61 (*CalCYP707A1/3*), EL65 (*CalCYP707A2*), EL67 (*CalCYP707A4*) with *Chenopodium album* Genbank accession numbers (BankIt sequence submissions #2664064 and #2668656). Naming of *C. album* GA-oxidase sequences was according to their top BLAST results for *A. thaliana* (Figure 5 and Supplementary Figure 7) which is not necessarily related to their subgroup in the phylogenetic trees (Supplementary Figure 6B). Naming of *C. album* NCED and CYP707A sequences was according to the subgroup in the phylogentic trees (Figure 6) and their top BLAST results for *A. thaliana* (Supplementary Figure 6B). Note that *CalNCED6B* and its putative *C. quinoa* ortholog AUR62017575 are both short sequences which are according to BLAST analyses NCED6 homologs (Supplementary Figures 4D and 6). For further details and references see the main text.

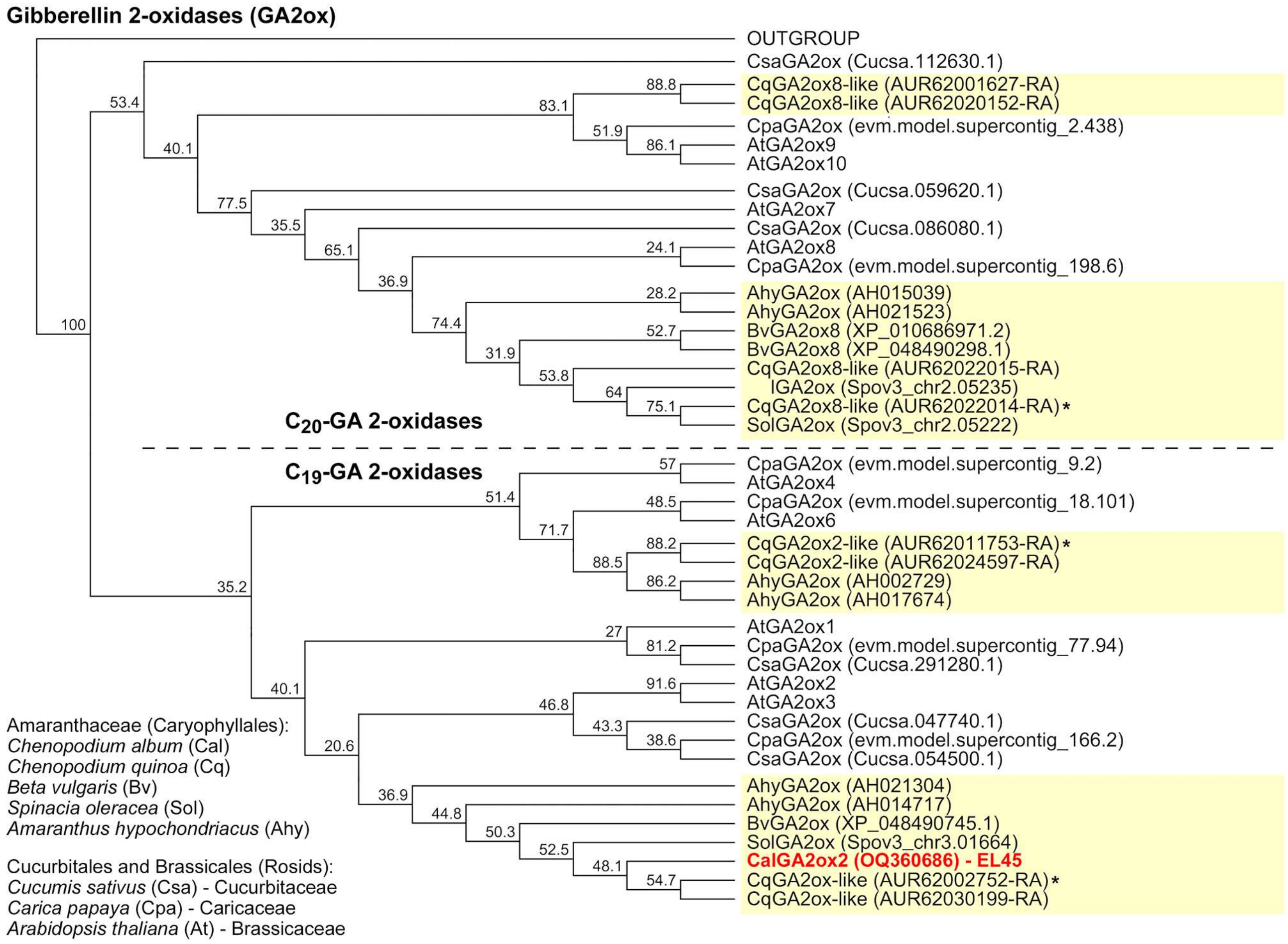

Supplementary Figure 7 | Phylogenetic tree of the predicted amino acid sequences of GA 2-oxidases (GA2ox). Known and putative amino acid NCED **(A)** and CYP707A **(B)** sequences of *Chenopodium quinoa*, *Chenopodium album* and other Amaranthaceae plus selected Brassicales and Cucurbitales species (as indicated) were aligned using ClustalW and Neighbor-Joining trees were built as described in methods. *Chenopodium quinoa* (Supplementary Figure 4) and the *C. album* (Supplementary Figure 6) sequence (*CalGA2ox2* - EL45) representing the C_{19}-GA 2-oxidase and C_{20}-GA 2-oxidase subgroups were identified. An * indicates identified DEGs during *C. quinoa* seed germination (Supplementary Figure 5).

Supplementary Table 1 | Primers used for PCR cloning of *Chenopodium album* sequences.

Name of amplified *C. album* sequence	Primer type	Primer sequence (5'-3')	Genbank acession number [b]
**EL23** (*PUB33-like*) [a]	Forward	GCTCATATCCCGGCACAAGA	OQ360687
	Reverse	TAAGCCTCCTCCTTAGCGGT	
**EL25** (*ACT7-like*) [a]	Forward	GTCATGGTTGGTATGGGCCA	OQ360688
	Reverse	GTGAAGGCTGGAAGAGGACC	
**EL27** (*CDC27B*) [a]	Forward	TTTTCAGCGAGCTGTGCAAC	OQ360689
	Reverse	GCAACTTTTCAATGGCAGCC	
**EL02**	Forward	CTTGACTCTCCAAAGAAAGCTTCA	OQ351932
	Reverse	AAGCTTGACTGTCGCAACCA	
**EL49**	Forward	ACAAGCTCTCCCTGTAAGCG	OQ360683
	Reverse	TCGCCCTAGTAAGTTCCGGT	
**EL07**	Forward	TGCACCGGTTCAGGAACATC	OQ351933
	Reverse	CCTCATTTTCCTCCCATGCATTC	
**EL16**	Forward	GGAGCCGTGGCCTAAATGTA	OQ351934
	Reverse	AGGAACCCTTGAAGGCATGC	
**EL61**	Forward	AGAGAATCCCTCACCGACGA	OQ351929
	Reverse	TTGCCAGTTCATTGCCAGGA	
**EL65**	Forward	GCCCCTAAATTTCCCCGGAA	OQ351930
	Reverse	CTGCGCAATGGTGAATGCTT	
**EL67**	Forward	TACTTTGGATTCGTGGGCCG	OQ351931
	Reverse	TGCTGGCCATCCTTAAGCTC	
**EL51**	Forward	CGTGCTCAGTTGAGTCAGGT	OQ360685
	Reverse	GGTGGGTGGCATCAATGAGA	
**EL12**	Forward	CCTGAATCGCATACATGGGC	OQ351937
	Reverse	TGGGGCTCGTAAAACCTTCA	
**EL13**	Forward	ACTGCCTTCCCTCTGAGACA	OQ351938
	Reverse	GCAAGCAATCATGAACCGCA	
**EL36**	Forward	CGGGTTCGATTCGATGCAAC	OQ360684
	Reverse	CTAGGTCGAACAGCTTGCCA	
**EL37**	Forward	AGAAACTCGACGCCCCAATT	OQ351936
	Reverse	GCATCATTGCGAGGTTCGAC	
**EL39**	Forward	AAGCCTCATTGACCCGGAAG	OQ351935
	Reverse	CACTTTGTCCTTGCTTGGGC	
**EL45**	Forward	ACAGCTGGAAGACGAAGCTC	OQ360686
	Reverse	TCCTTCCGCCATTGTTTCCA	

[a] Reference gene. [b] *Chenopodium album* BankIt sequence submissions #2664064 and #2668656.

Supplementary Table 2 | Primer sequences used for the *Chenopodium album* RT-qPCR analysis.

***C. album* gene name (sequence name)**	**Primer type**	**Primer sequence (5'-3')**	**Amplicon length (bp)** [b]
***CalPUB33-like* (EL23)** [a]	Forward	TGCTTTCTGCCACATCTGGT	142
	Reverse	ACACAGTCCAAGGATGCAGG	
***CalACT7-like* (EL25)** [a]	Forward	GTCTCGTGGATACCTGCAGC	168
	Reverse	AGCTGAGAGATTCCGTTGCC	
***CalCDC27B* (EL27)** [a]	Forward	CATGTATGATAAGGCCATGTTGCAT	117
	Reverse	GCAAGAAGTTACCTTTATGGTGGC	
***CalNCED3* (EL02)**	Forward	CGTGATTGCGAAATCGTGCA	168
	Reverse	GCGCATCCTAAGGTAGACCC	
***CalNCED5* (EL49)**	Forward	GTGATGGGATGGTCCATGCA	153
	Reverse	CAATCCCCGAATGTCCGTGA	
***CalNCED6A* (EL07)**	Forward	GGAGTGGCTAATGCTGGTGT	159
	Reverse	GTGTGCAATCAACGGGCAAT	
***CalNCED6B* (EL16)**	Forward	ACAAAGCATGGTTCCCCACC	108
	Reverse	GGAGCCGTGGCCTAAATGTA	
***CalCYP707A1/3* (EL61)**	Forward	GGGTACCTAATTCCTAAAGGGTGG	97
	Reverse	AAGGGTCAAACTTCTCAGGCTC	
***CalCYP707A2* (EL65)**	Forward	AAGGAAGCTTCTAAATGGGGACAT	194
	Reverse	GCAGTAGTATCATGAGCAGCGA	
***CalCYP707A4* (EL67)**	Forward	GGCAACAAAGGACCTTCTTGGA	117
	Reverse	GCTGTATCTTGAGCAGCAAACAGAA	
***CalGA3ox1/2* (EL51)**	Forward	TGGTGTTACGGGTTATGGCC	114
	Reverse	GGCCAAAGTTGACGAGCATG	
***CalGA3ox3/4* (EL12)**	Forward	TTGCAATCCCGTGGTTTGTG	131
	Reverse	TGCCAATGAGAAGTCGCTGT	
***CalGA3ox1/4* (EL13)**	Forward	CTCATATCCACCGTGCCCTG	108
	Reverse	TGGAGGCCACTTACATTGCC	
***CalGA20ox3* (EL37)**	Forward	ACGTTGTCTTTCCCTCACCG	72
	Reverse	GCCTAAAGCCGAGGTGAAGT	
***CalGA20ox2* (EL39)**	Forward	CTAGTGGGTGAGGCTTGCC	81
	Reverse	GGCATCCCTAACCAACTCGG	
***CalGA2ox2* (EL45)**	Forward	CGTAATGGCGACGTTGGTTG	139
	Reverse	ACAGAGGAAAACAGAGTAGAGCG	

[a] Reference gene. [b] Amplicon length based on *C. album* sequences verified by gel electrophoresis.
